# Studies of Glyoxalase 1-Linked Multidrug Resistance Reveal Glycolysis-Derived Reactive Metabolite, Methylglyoxal, Is a Common Contributor in Cancer Chemotherapy Targeting the Spliceosome

**DOI:** 10.3389/fonc.2021.748698

**Published:** 2021-11-01

**Authors:** Muhanad Alhujaily, Hafsa Abbas, Mingzhan Xue, Alberto de la Fuente, Naila Rabbani, Paul J. Thornalley

**Affiliations:** ^1^ College of Applied Medical Sciences, University of Bisha, Bisha, Saudi Arabia; ^2^ Clinical Sciences Research Laboratories, Warwick Medical School, University of Warwick, University Hospital, Coventry, United Kingdom; ^3^ Diabetes Research Center, Qatar Biomedical Research Institute, Hamad Bin Khalifa University, Qatar Foundation, Doha, Qatar; ^4^ Department of Basic Medical Science, College of Medicine, QU Health, Qatar University, Doha, Qatar; ^5^ Biomedical & Pharmaceutical Research Unit, QU Health, Qatar University, Doha, Qatar

**Keywords:** methylglyoxal, glyoxalase, cancer chemotherapy, multidrug resistance, proteomics

## Abstract

**Background:**

Tumor glycolysis is a target for cancer chemotherapy. Methylglyoxal (MG) is a reactive metabolite formed mainly as a by-product in anaerobic glycolysis, metabolized by glyoxalase 1 (Glo1) of the glyoxalase system. We investigated the role of MG and Glo1 in cancer chemotherapy related in multidrug resistance (MDR).

**Methods:**

Human Glo1 was overexpressed in HEK293 cells and the effect on anticancer drug potency, drug-induced increase in MG and mechanism of cytotoxicity characterized. Drug-induced increased MG and the mechanisms driving it were investigated and the proteomic response to MG-induced cytotoxicity explored by high mass resolution proteomics of cytoplasmic and other subcellular protein extracts. Glo1 expression data of 1,040 human tumor cell lines and 7,489 tumors were examined for functional correlates and impact of cancer patient survival.

**Results:**

Overexpression of Glo1 decreased cytotoxicity of antitumor drugs, impairing antiproliferative activity of alkylating agents, topoisomerase inhibitors, antitubulins, and antimetabolites. Antitumor drugs increased MG to cytotoxic levels which contributed to the cytotoxic, antiproliferative mechanism of action, consistent with Glo1-mediated MDR. This was linked to off-target effects of drugs on glycolysis and was potentiated in hypoxia. MG activated the intrinsic pathway of apoptosis, with decrease of mitochondrial and spliceosomal proteins. Spliceosomal proteins were targets of MG modification. Spliceosomal gene expression correlated positively with Glo1 in human tumor cell lines and tumors. In clinical chemotherapy of breast cancer, increased expression of Glo1 was associated with decreased patient survival, with hazard ratio (HR) = 1.82 (logrank *p* < 0.001, *n* = 683) where upper quartile survival of patients was decreased by 64% with high Glo1 expression.

**Conclusions:**

We conclude that MG-mediated cytotoxicity contributes to the cancer chemotherapeutic response and targets the spliceosome. High expression of Glo1 contributes to multidrug resistance by shielding the spliceosome from MG modification and decreasing survival in the chemotherapy of breast cancer. Adjunct chemotherapy with Glo1 inhibitor may improve treatment outcomes.

## Introduction

Cancer is increasing in incidence and mortality worldwide (1). Although improvements have been made in cancer chemotherapy, drug resistance and the resulting decline in effectiveness of drug treatment are considered to contribute to 90% of cancer-related deaths (2). Countering drug resistance effectively is limited by its multifactorial mechanisms which may change in relative importance as tumor stage advances. A common feature of resistance to antiproliferative, cytotoxic antitumor drugs is a cytoprotective response (3). Tumor cell metabolism, including glycolysis, is an influential factor in efficacy of cytotoxic antitumor drug (4), influencing response and development of resistance (5, 6). Formation and metabolism of the glycolysis-linked reactive metabolite, methylglyoxal (MG), has a historical link to the glyoxalase pathway in tumor metabolism, although its role in cancer treatment and multidrug resistance (MDR) is not fully understood (7).

**Figure 1:**
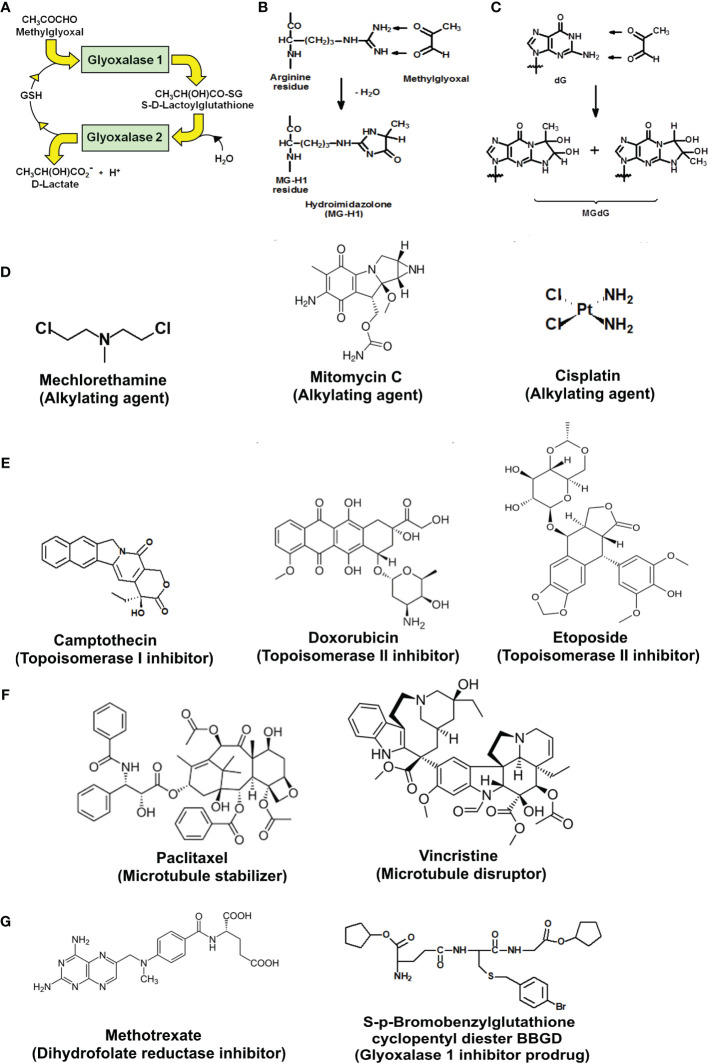
Metabolism of methylglyoxal by the glyoxalase pathway, major glycation reactions of methylglyoxal and clinical and investigational antitumor drugs. **(A)** Metabolism of methylglyoxal by the glyoxalase pathway. **(B)** Glycation of arginine residues by methylglyoxal from hydroimidazolone, MG-H1. **(C)** Glycation of guanyl moieties of DNA by methylglyoxal from isomeric imidazopurinones, MGdG. **(D–G)** Molecular structures of antitumor drugs. **(D)** Alkylating agents: mechlorethamine, mitomycin C, and cisplatin. **(E)** Topoisomerase inhibitors: camptothecin, doxorubicin, and etoposide. **(F)** Antitubulins: paclitaxel and vincristine. **(G)** Antimetabolites: methotrexate and glyoxalase 1 inhibitor prodrug, *S*-*p*-bromobenzylglutathione cyclopentyl diester (BBGD).

The glyoxalase system catalyzes the metabolism of the reactive dicarbonyl metabolite, methylglyoxal (MG), to D-lactate. Glyoxalase 1 (Glo1) catalyzes the glutathione (GSH)-dependent metabolism of MG to S-D-lactoylglutathione. Glyoxalase 2 (Glo2) catalyzes the hydrolysis of S-D-lactoylglutathione to D-lactate, reforming GSH consumed in the Glo1-catalyzed step (**Figure 1A**). MG is formed spontaneously by trace-level degradation of triosephosphate glycolytic intermediates, glyceraldehyde-3-phosphate (GA3P) and dihydroxyacetonephosphate (DHAP), and modifies proteins and DNA to form arginine-derived hydroimidazolone, *N*_δ_-(5-hydro-5-methyl-4-imidazolon-2-yl)ornithine (MG-H1), and deoxyguanosine-derived imidazopurinone, 3-(2′-deoxyribosyl)-6,7-dihydro-6,7-dihydroxy-6/7-methylimidazo-[2,3-b]purine-9(8)one (MGdG) as major adducts (**Figures 1B, C**). Glo1 suppresses the steady-state cellular concentration of MG and thereby the steady-state levels of MG-derived protein and DNA adducts to low, tolerable levels. In previous studies, it has been shown that inhibition of Glo1 in tumor cells *in vitro* leads to the accumulation of MG, increasing protein and DNA modification (8, 9) and apoptosis (10–12). Treatment of human tumor cells with cell permeable Glo1 inhibitor pro-drug, *S*-*p*-bromobenzylglutathione cyclopentyl diester (BBGD)—which delivers the Glo1 competitive inhibitor, *S*-*p*-bromobenzylglutathione (*K*_i_ = 160 nM) into cells, increased the cellular concentration of MG, protein, and DNA adducts and induced apoptosis (8, 9).

In this study, we explore the effect of overexpression of Glo1 in the antiproliferative activity of clinical antitumor agents, the contribution of MG-induced cytotoxicity in cancer chemotherapy and mechanism of MG increase and cytotoxic response, and Glo1 expression as a risk predictor on cancer chemotherapy outcomes in clinical breast cancer.

## Methods

### Antibodies, Anticancer Drugs, and Other Reagents

Rat monoclonal anti-Glo1 antibody (SAB4200193) and anti-Rat IgG-biotin conjugate (B7139) were from Sigma-Aldrich (Poole, Dorset, UK). Human cytochrome *c* ELISA kit (QIA74) was from Oncogene Research Products (San Diego, CA, USA). Mechlorethamine (C2942) was from Cambridge Bioscience Ltd. (Cambridge, UK). Mitomycin C (M4287, from *Streptomyces caespitosus*), cisplatin (479306), *S*-(+)-camptothecin (C9911), doxorubicin (D1515), etoposide (E1383), paclitaxel (T7402), vincristine (V8879), and methotrexate (06563) were from Sigma-Aldrich. *S*-*p*-Bromobenzylglutathione cyclopentyl diester was prepared in-house (8). D-Lactic dehydrogenase (L9636), pepsin (EC 3.4.23.1; porcine stomach mucosa; P6887), pronase E (EC 3.4.24.31, type XIV, from *Streptomyces griseus*; P5147), prolidase (EC 3.4.13.9; porcine kidney; P6675), and leucine aminopeptidase (EC 3.4.11.2, type VI, porcine kidney, L9776) were from Sigma-Aldrich. Geneticin (700 µg G-418) was from Fisher Scientific (Loughborough, UK). The HEK293 cell line (CRL-1573) was ATCC (Virginia, USA), and vectors pIRES2-GLO1-EGFP and pIRES2-EGFP were prepared in-house (13).

### Culture of HEK293 Cells *In Vitro*

The HEK293 cell line, seeding density 2 × 10^4^ cells cm^−2^, was cultured in Dulbecco’s modified Eagle’s medium (DMEM) containing phenol red, L-glutamine, and 4,500 mg/l glucose, supplemented with 10% fetal bovine serum (FBS), 100 U penicillin, and 0.1 mg/ml streptomycin. pIRES2-GLO1-EGFP plasmid (Glo1+ vector) and pIRES2-EGFP plasmid (empty vector) were prepared as described (13). HEK293 cells stably transfected with Glo1 expression plasmid pIRES2-GLO1-EGFP and empty vector pIRE2-EGFP using Lipofectamine 2000 according to the manufacturer’s instructions (plasmid DNA: Lipofectamine 2000, 1:4). After 48 h, cells were subcultured with G-418 disulfate supplement. Transfected colonies with GFP fluorescence were selected using a cloning disc (3.2 mm) and glass cylinder selector (8 mm, 150 µl) and cultured further with G-418 disulfate-containing medium. Assessment of Glo1 activity and protein, as described (14, 15), indicated a four- to fivefold increase in Glo1 activity and protein, as previously reported (16). HEK293 cells stably tranfected with empty and GLO1+ vectors were incubated for 2 days with and without the anticancer drugs at the concentrations indicated—diluted from 100 mM stock solution in dimethylsulfoxide (DMSO), except dimethylformamide (DMF) for cisplatin. The effect on cell growth was assessed by viable cell number counts, using the Trypan blue exclusion method and median growth inhibitory concentrations GC_50_ deduced. For BBGD, cultures were also performed under an atmosphere of 3% oxygen as a model of hypoxia. HEK293 cells were incubated with 15–400 µM MG for 48 h to determine the GC_50_ of MG and with 131 µM MG for 6, 12, and 24 h with medium replacement and continued culture for 48 h to determine the period of exposure to MG required for growth arrest and cytotoxicity. Cellular MG concentration and flux of formation of D-lactate, a surrogate measure of flux of formation of MG, was assayed as described (17, 18).

### Preparation of Cellular Protein Extracts for Proteomics

Protein extracts of total cell cytoplasm, nuclei, mitochondrial matrix, and intermembrane space and mitochondrial membranes were prepared from HEK293 cells (3 × 10^6^) incubated with and without 131 µM MG for 6 h by adaptation of protocols for subcellular fraction proteomics (19). Protein abundances were analyzed by label-free high-resolution Orbitrap mass spectrometry of tryptic digests, as described (20).

For cytoplasmic extracts, cell pellets were suspended in 10 mM sodium phosphate buffer, pH 7.0, sonicated on ice (110 W, 30 s, 4°C), and membranes sedimented by centrifugation (20,000×*g*, 30 min, 4°C). An aliquot of protein (300 µg) was washed with argon-purged water by ultradiafiltration over a 10-kDa cutoff membrane microspin filter (14,000×*g*, 20 min, 4°C); 4 cycles of concentration to 50 µl and 10-fold dilution with water. An aliquot of washed protein (100 µg) was used in proteomics analysis.

For the nuclear extract, cells were washed in PBS, resuspended in buffer (0.3 M sucrose, 10 mM MgCl_2_, 50 mM Tris-HCl, pH 7.8, and 0.1 mM phenylmethylsulphonyl fluoride (PMSF); 1 ml, 4°C), homogenized in a Teflon-glass homogenizer, and centrifuged (1,000×*g*, 10 min, 4°C). The pellet was suspended in further buffer without PMSF (0.1 ml) and layered onto a “sucrose cushion” of 2 M sucrose in the same buffer; 1 ml. After centrifugation (16,000×*g*, 20 min, 4°C), the supernatant was discarded, the pellet of purified nuclei washed twice in PBS, resuspended in buffer (20 mM HEPES, pH 7.9, 1.5 mM MgCl_2_, 0.5 M NaCl, 0.2 mM EDTA, and 20% glycerol; 25 µl) and incubated for 30 min with gentle rocking at 4°C. Nuclei were then lysed with 10 passages through an 18-gauge needle, the lysate centrifuged (9,000×*g*, 30 min 4°C), and the supernatant used for proteomics analysis.

For mitochondrial protein extracts, cell pellets were resuspended in isotonic buffer (25 M sucrose, 5 mM Tris-HCl, pH 7.5, and 0.1 mM PMSF; 1 ml) and disrupted in a glass-Teflon homogenizer. Unbroken cells and nuclei were sedimented by centrifugation (600×*g*, 15 min, 4°C) and supernatants centrifuged again (10,000×*g*, 25 min, 4°C) to collect the pellet of mitochondria. The mitochondrial pellet was washed once with isotonic buffer containing 1 mM EDTA, pH 7.5; 1 ml. For the mitochondrial matrix and intermembrane space protein extract, samples were suspended in lysis buffer (1 mM dithiothreitol (DTT) and 10 mM HEPES pH 7.4; 200 µl), incubated at 4°C for 30 min, and then sonicated (110 W, 3 × 30 s, 4°C) and centrifuged (1 h, 120,000×*g*, 4°C). The supernatant was transferred to a 3-kDa cutoff filter and washed by ultradiafiltration at 4°C, concentrating finally to 25 μl and the washed protein used in proteomics analysis. For the mitochondrial membrane protein extract, the membrane pellet was treated with membrane extraction buffer (20 mM Tris-HCl, pH 7.4; 0.4 M NaCl, 15% glycerol, 1 mM DTT, and 1.5% Triton-X-100; 0.2 ml) and samples shaken gently at 4°C for 1 h. Samples were then centrifuged (20,000×*g*, 4°C, 1 h). The supernatant was retained and protein washed by ultradiafiltration in 1.5% Triton-X100. Protein content was quantified with EZQ™ method and Triton-X100 removed by extraction with ethylacetate (21) before proteomics analysis.

### Proteomics Analysis

Protein extracts (100 µg) were reduced by treatment with DTT (6 μl, 6 mM) in the dark for 30 min at 37°C, and then alkylated by treatment with iodoacetamide (5.9 µl, 10.8 mM). Residual iodoacetamide was quenched by further addition of DTT (5.9 μl, 6 mM). Lys-C protease (1 mg/ml, 5 µl) in 500 mM ammonium bicarbonate, pH 8.0, was added, and samples were incubated for 1 h at 37°C. TPCK-treated trypsin (1 mg/ml, 5 µl) in 1 mM calcium chloride/500 mM ammonium bicarbonate, pH 8.0, was then added, and samples were incubated at 37°C for 5 h. An aliquot of 10% trifluoracetic acid in water (5 µl) was added to stop the reaction. Samples were then lyophilized to dryness and resuspended in 0.1% formic acid in water. Processed cell lysate samples were submitted for a label-free proteomic quantitation analysis by reversed phase nanoflow liquid chromatography-mass spectrometry-Orbitrap Fusion mass spectrometer equipped with a microspray source operating in positive ion mode, as previously described (18).

For the peptide ion data search, Mascot (Matrix Science, version 2.5.0) was used against a *Homo sapiens* database (http://www.uniprot.org/), with product ions and precursor error mass ±5 ppm and ±0.8 Da and allowance made for six trypsin-missed cleavages, carbamidomethylated cysteine, MG-H1, and methionine sulfoxide residues. Only fully tryptic peptide matches were included. Scaffold (version Scaffold 4.3.2, Proteome Software Inc.) was used to validate MS/MS peptide fragmentation and protein identification (false discovery rate (FDR) <5%) and ≥2 unique peptides, with detection probabilities assigned using protein prophet algorithm (22). Label-free quantitation of protein abundances were determined in three independent biological replicate samples using Progenesis QI for proteomics 2.0 software (Nonlinear Dynamics, Newcastle, UK). For the pathway enrichment analysis in transcriptomic and proteomic datasets, pathway analysis was performed using Database for Annotation, Visualization and Integrated Discovery v6.8 (https://david.ncifcrf.gov/) (23). KEGG and REACTOME analyses were used for pathway enrichment analysis and INTERPRO analysis for enrichment of protein domains. Pathway and domain enrichment were considered significant with FDR <0.05.

### Cancer Bioinformatics Database CCLE

Data on gene copy number and expression (RNA-seq) in human tumor cell lines were extracted from the Cancer Cell Line Encyclopedia (CCLE) database (https://portals.broadinstitute.org/ccle) for 1,040 human tumor cell lines. Correlation analysis was performed using the R program and Pearson correlation analysis performed on mRNA copy number expressed as reads per kilobase million (RPKM). Data for 10,758 genes were available and a Bonferroni correction applied on outcomes. A Protein Interaction Network was prepared for 340 genes with strong positive correlation with Glo1 expression. The gene list was submitted to STRING (https://string-db.org/) and a network among them prepared based on “experiments” and “databases” as active sources with the highest confidence level (interaction score >0.9). This resulted in a network with 1,617 edges and 322 nodes (18 identifiers could not be mapped) with an average node degree of 10. The network was clustered using the Markov Clustering Algorithm with inflation parameter set to three and the nodes were colored according to the enriched KEGG pathways indicated.

### Kaplan-Meier Survival Analysis Database

The Kaplan-Meier plotter analysis tool (http://kmplot.com/analysis/) was used for the analysis of the impact of Glo1 and Glo2 expression on overall survival of breast cancer patients treated with chemotherapy. Correlation analysis was also performed for expression of Glo1 with PPIL1, CDC5L, and TRADD in pan cancer database (7,489 multiple types of human tumor; RNA seq) and breast cancer (*n* = 4,939, gene chip). The output variables were number of patients, hazard ratio and upper quartile survival (months) for low/high gene expression cohorts, and Logrank *p*-value (24, 25). Chemotherapy received by patients in this dataset was doxorubicin, mitomycin C, and alkylating agents, cyclophosphamide and thiotepa.

### Statistical Analysis

Data are expressed as mean ± SD or SEM, as indicated. Statistical analyses were performed with SPSS, version 24. Normality of data distribution was assessed using Kolmogorov-Smirnov test. Comparisons between two groups were performed using two-tailed Student’s *t*-test for normally distributed data and Mann-Whitney *U* test for non-normally distributed data. Differences between multiple groups with one variable were determined using one-way ANOVA for normally distributed data and Kruskal-Wallis test for nonnormally distributed data. Correlation analysis used Pearson and Spearman methods for normally distributed and nonnormally distributed data, respectively.

## Results

### Overexpression of Glyoxalase 1 in HEK-293 Cells Decreases Antiproliferative Activity of Clinical Antitumor Drugs

**Figure 2:**
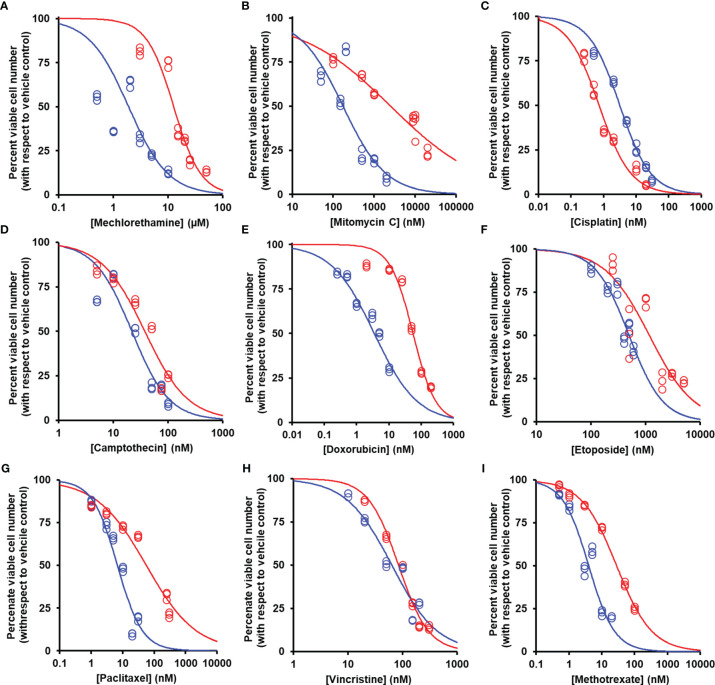
Suppression of antiperoxidative activity of clinical antitumor drugs by overexpression of glyoxalase. Color code for blue data points and curve: blue, empty vector transfected; red, Glo1+ vector transfected. **(A)** Mechlorethamine. GC_50_ = 1.90 ± 0.11 µM, *n* = 1.20 ± 0.09 (empty vector) and GC_50_ = 12.70 ± 0.90 µM, *n* = 1.75 ± 0.26 (Glo1+; sevenfold resistance). **(B)** Mitomycin C. GC_50_ = 174 ± 29 nM, *n* = 0.83 ± 0.12 (empty vector) and GC_50_ = 2,541 ± 360 nM, *n* = 0.39 ± 0.03 (Glo1+; 15-fold resistance). **(C)** Cisplatin. GC_50_ = 3.17 ± 0.35 µM, *n* = 0.92 ± 0.04 (empty vector) and GC_50_ = 0.73 ± 0.05 µM, *n* = 0.89 ± 0.07 (Glo1+; 0.2-fold resistance). **(D)** Camptothecin. GC_50_ = 21.8 ± 2.2 nM, *n* = 1.28 ± 0.15 (empty vector) and GC_50_ = 37.6 ± 3.3 nM, *n* = 1.12 ± 0.12 (Glo1+; twofold resistance). **(E)** Doxorubicin. GC_50_ = 3.54 ± 0.28 nM, *n* = 0.71 ± 0.05 (empty vector) and GC_50_ = 55.9 ± 3.4 nM, *n* = 1.24 ± 0.10 (Glo1+; 16-fold resistance). **(F)** Etoposide. GC_50_ = 500 ± 32 nM, *n* = 1.41 ± 0.19 (empty vector) and GC_50_ = 1,170 ± 169 nM, *n* = 1.06 ± 0.17 (Glo1+; twofold resistance). **(G)** Paclitaxel. GC_50_ = 6.8 ± 1.0 nM, *n* = 1.07 ± 0.17 (empty vector) and GC_50_ = 56.4 ± 7.2 nM, *n* = 0.55 ± 0.04 (Glo1+; eightfold resistance). **(H)** Vincristine. GC_50_ = 63.4 ± 5.2 nM, *n* = 1.04 ± 0.10 (empty vector) and GC_50_ = 83.7 ± 3.3 nM, *n* = 1.57 ± 0.10 (Glo1+; 1.3-fold resistance). **(I)** Methotrexate. GC_50_ = 4.02 ± 0.35 nM, *n* = 1.10 ± 0.10 (empty vector) and GC_50_ = 28.5 ± 0.9 nM, *n* = 0.81 ± 0.02 (Glo1+; sevenfold resistance). Data were fitted by nonlinear regression to the dose-response equation: Viable cell number (% of control) = 100 × GC_50_^n^/(GC_50_^n^ + [Drug]^n^), solving for GC_50_ and *n* (logistic regression coefficient) and plotting the outcome dose-response curves given (six drug concentrations in triplicate; *n* = 18). GC_50_ values are summarized (**Table 1**). Data for doxorubicin and paclitaxel and evidence of increased Glo1 protein in the Glo1+ cell line have been published previously (16).

**Figure 3:**
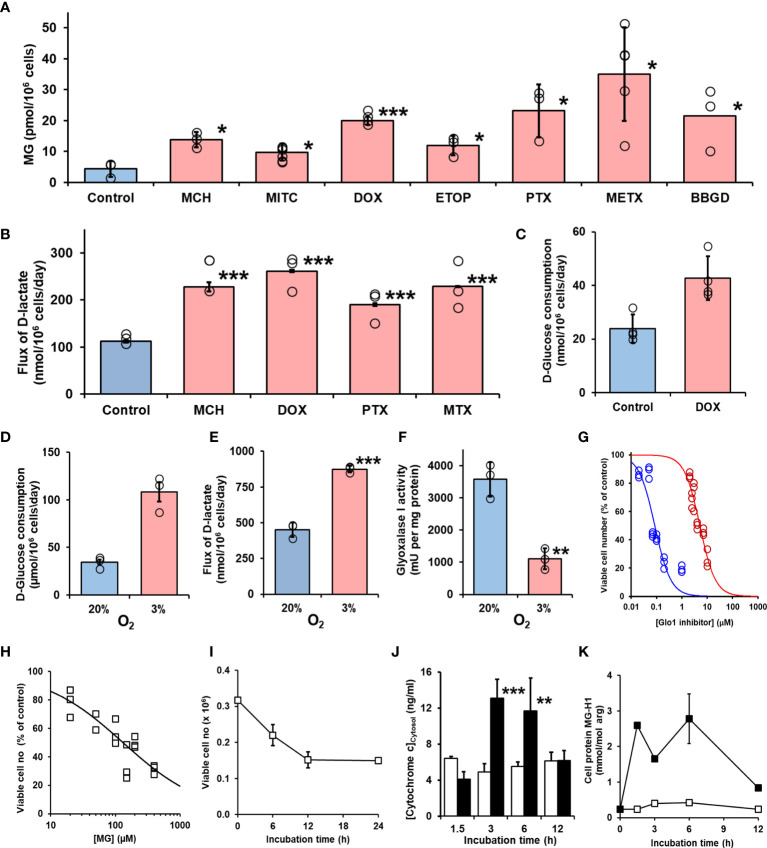
Induction of cellular dicarbonyl stress by antitumor drugs and mechanism methylglyoxal-induced cytotoxicity. **(A)** Increase in cellular MG in HEK293 cells treated with antitumor drugs for 3 h. Control (no drug added) and *ca.* 2 × GC_50_ concentration in nontransfected cells: mechlorethamine (MCH, 4.8 µM), mitomycin C (MITC, 316 nM), doxorubicin (DOX, 6.0 nM), etoposide (ETOP, 323 nM), paclitaxel (PTX, 21 nM), methotrexate (METX, 7.6 nM), and BBGD (7.4 µM). Data are mean ± SEM (*p* < 0.001; *one-way ANOVA*; *n* = 3 except *n* = 4, 5, and 6 for DOX, METX, and MITC, respectively). **(B)** Flux of formation of D-lactate (surrogate for flux of MG) and **(C)** flux of glucose consumption incubated *in vitro* with and without investigational agent and drugs indicated. Cells were incubated for 24 h with and without 2 × GC_50_ concentration. Data are mean ± SEM (*n* = 3). **(D–G)** Effect of hypoxia on formation and metabolism of methylglyoxal and antiproliferative activity of BBGD. **(D–F)**) Flux of glucose consumption and formation of D-lactate and Glo1 activity of HEK293 cells incubated for 72 h in 20% and 3% oxygen, models of normoxia and hypoxia, respectively. Data are mean ± SEM (*n* = 3). **(G)** Dose-response curve for BBGD. The GC_50_ values for BBGD under normoxic and hypoxic conditions were 5.12 ± 0.33 µM and 0.085 ± 0.010 μM, respectively (*N* = 18). **(H)** Dose-response curve for MG-treated HEK293 cells. GC_50_ 131 µM ± 19.1, with logistic regression coefficient *n* = 0.70 ± 0.05 (*N* = 18). **(I)** Effect of duration of exposure to MG on antiproliferative activity in HEK293 cells *in vitro*. Cells were incubated with 131 µM MG. Data are mean ± SD (*n* = 3). **(J)** Release of cytochrome c from mitochondrial in early-stage MG-induced toxicity. HEK293 cells were incubated with and without MG *in vitro* and cytosolic cytochrome c assayed by ELISA. Key: hollow bars, control; solid bars, +131 µM MG. Data are mean ± SD (n = 3). Significance: *p < 0.05, **p < 0.01, and ***p < 0.001 with respect to control (*t*-test). **(K)** Early-stage accumulation of glycated modified during MG-induced cytotoxicity of HEK293 cells *in vitro*s. Key: open symbols, control; filled symbols, +131 µM MG. Data are mean ± SD (n = 3).

We studied the effect of overexpression of Glo1 on the antiproliferative, cytotoxic activity of antitumor drugs: alkylating agents—mechlorethamine, mitomycin C, and cisplatin (**Figure 1D**); topoisomerase inhibitors—camptothecin, doxorubicin, and etoposide (**Figure 1E**); antitubulins—paclitaxel and vincristine (**Figure 1F**); and antimetabolite—methotrexate (**Figure 1G**). We found overexpression of Glo1 decreased the antiproliferative activity for most anticancer drugs. In order of increasing effect, MDR (fold increase in GC_50_) was vincristine, 1.3-fold; etoposide, 2-fold; mechlorethamine and methotrexate, 7-fold; paclitaxel, 8-fold; mitomycin C, 15-fold; and doxorubicin, 16-fold (**Figures 2A**–**I** and **Table 1**). MG is the major dicarbonyl endogenous substrate metabolized by Glo1 (26). This finding suggests that anticancer drugs may increase steady-state cellular concentrations of MG to toxic levels as part of their mechanism of action. To test this, we measured the cellular concentration of MG in HEK293 cells after treatment for 3 h with drugs for which Glo1 overexpression produced resistance. Cellular MG concentration was increased twofold with mitomycin C, threefold with mechlorethamine and etoposide, fivefold with doxorubicin and paclitaxel, and eightfold with methotrexate (**Figure 3A**). Increased MG concentration is often linked to inhibition of Glo1, as in the mechanism of action of BBGD (8) and/or increased formation of MG by increased glycolysis (18). BBGD increased the endogenous concentration of cellular MG by fivefold and also had cytotoxic, antiproliferative activity: GC50 = before 5.12 ± 0.33 µM (*n* = 18). None of the drugs studied were potent inhibitors of Glo1 over the concentration range studied (**Supplementary Figure S1**). Rather, the increase of MG may be related to increased flux of formation of MG, as reflected in increased flux of formation of D-lactate—as surrogate measure of flux of formation of MG (27). To explore this, we studied the effect of the 2 × GC_50_ concentration of anticancer drugs on the flux of formation of D-lactate. The flux of formation of D-lactate over the initial 24 h of drug treatment was increased *ca.* twofold by treatment with mechlorethamine, doxorubicin, paclitaxel, and methotrexate (**Figure 3B**). Increased D-lactate often reflects a proportionate increase in metabolism of glucose through glycolysis (18, 28). To test this, we measured the flux of consumption of glucose by HEK293 cells in the initial 24 h of treatment with doxorubicin and found that the flux of glucose consumption was indeed increased twofold, proportionate to increase of flux of D-lactate (**Figure 3C**). This suggests doxorubicin increased the cellular concentration of MG by increasing the flux of formation of MG as a consequence of the increased of flux of glucose metabolism through glycolysis.

**Table 1:** Median growth inhibitory concentration of anticancer drugs of HEK-293 cells *in vitro*—effect of overexpression of glyoxalase 1.

	Anticancer drug	GC_50_		
		Empty vector	Glo1+	Fold MDR
Alkylator	Mechlorethamine (µM)	1.90 ± 0.11	12.70 ± 0.90^***^	7
	Mitomycin C (nM)	174 ± 29	2541 ± 360^***^	15
	Cisplatin (µM)	3.17 ± 0.35	0.73 ± 0.05^***^	0.2
Topoisomerase inhibitor	Camptothecin (nM)	21.8 ± 2.2	37.6 ± 3.3^***^	2
	Doxorubicin (nM)	3.54 ± 0.28	55.9 ± 3.4^***^	16
	Etoposide (nM)	500 ± 32	1170 ± 169^***^	2
Antimicrotubule	Paclitaxel (nM)	6.8 ± 1.0	56.4 ± 7.2^***^	8
	Vincristine (nM)	63.4 ± 5.2	83.7 ± 3.3^***^	1.3
Antimetabolite	Methotrexate (nM)	4.02 ± 0.35	28.5 ± 0.9^***^	7

HEK293 cells with stable transfection by empty vector (Empty vector) or vector producing four- to fivefold increased expression of Glo1 (Glo1+) were incubated with and without treatment with anticancer drugs, six different concentrations in triplicate, for 48 h. The median growth inhibitory concentration of the drug, GC_50_, was deduced by nonlinear regression of viable cell number (V) on drug concentration for the dose-response equation, $V=100\times {\text{GC}}_{50}^{n}/({\text{GC}}_{50}^{n}+{\left[\text{Drug}\right]}^{n})$ , solving for GC_50_ and logistic regression coefficient (n). n values are omitted for brevity. Significance: ^***^p < 0.001 (Student’s t-test).

The major source of formation of MG is anaerobic glycolysis and the flux of glucose metabolism through glycolysis and hence formation of MG is increased in hypoxia. Expression of Glo1 is also downregulated in hypoxia inducible factor-1α (29). We explored the effect of low oxygen concentration, an atmosphere of 3% oxygen, compared with culture under normoxic atmosphere containing 20% oxygen on MG-related metabolism and potency of BBGD. In 3% oxygen atmosphere, the flux of glucose consumption and formation of D-lactate were increased twofold and the expression of Glo1 was decreased by 69%. The combined effect of increased MG formation and decreased Glo1 expression was associated with a concomitant increase in potency of the antiproliferative activity of BBGD of *ca.* 60-fold (**Figures 3D**–**G**).

### Mechanism of the Antiproliferative, Cytotoxic Activity of Methylglyoxal in HEK293 Cells *In Vitro*—Early-Stage Proteomic Response to MG-Induced Cytotoxicity

To explore the mechanism of cytotoxicity of MG, we initially characterized the potency and time course of commitment to the antiproliferative response in HEK293 cells *in vitro*. The GC_50_ of MG was 131 ± 19.1 µM (**Figure 3H**) and decrease of viability of HEK293 cells incubated with 131 µM MG reached maximum effect after exposure to MG for 12 h (**Figure 3I**). We studied cytochrome *c* increase in the cell cytosol as an indicator of activation of the mitochondrial, intrinsic pathway of apoptosis (30). The cytosolic concentration of cytochrome *c* was increased from 3 to 6 h with MG treatment and decreased thereafter (**Figure 3J**). We found the major MG-derived glycation adduct of cell protein, MG-H1 residues, increased rapidly within the initial 1.5 h, remained high until 6 h, and decreased to approach basal levels by 12 h of exposure to MG (**Figure 3K**). We then investigated the proteomic response in early-stage commitment to apoptosis by a label-free quantitative proteomics study of subcellular fractional proteome HEK293 cells treated with and without 131 µM MG for 6 h, examining total cytoplasmic and thereafter nuclear, mitochondrial matrix and intermembrane space, and mitochondrial membrane proteomes—including proteins with MG-H1 modification. Total protein counts detected, detected with MG-H1 modification and changed in abundance in all replicates of control and MG-treated cells are summarized (**Table 2**).

**Table 2:** Subcellular fractional proteomics of HEK293 cells treated with methylglyoxal.

Subcellular fraction	Total protein count (fold change)			Protein count with MG-H1 modification (fold change)		
	Total detected	Increased abundance	Decreased abundance	Total detected	Increased abundance	Decreased abundance
Cytoplasm	4,091	1,185 (1.2- to 30.5-fold)	121 (0.03- to 0.91-fold)	493	41 (1.2- to 14.0-fold)	69 (0.05- to 0.91-fold)
Nucleus	2,060	48 (1.2- to 263-fold)	59 (0.02- to 0.78-fold)	120	5^a^	1^a^
Mitochondrial matrix and intermembrane space	936	16 (1.4- to 49.3-fold)	132 (0.02- to 0.79-fold)	42	2^b^	1^b^
Mitochondrial membrane	168	9 (2.1–9.6)	116 (0.01–0.69)	26	2	9

Supporting protein lists (**Supplementary Tables S1**–**S11**). ^a^Proteins with MG-H1 modification were as follows: abundance increase—carbohydrate-response element-binding protein (Mondo A, 263-fold), protachykinin-1 (21-fold), mitochondrial 3-ketoacyl-CoA thiolase (15-fold), diphthamide biosynthesis protein-1 (7-fold), and mitochondrial enoyl-CoA hydratase (2-fold); abundance decrease—ABHD14A (0.08-fold). ^b^Proteins with MG-H1 modification were as follows: abundance increase—scaffold attachment factor (eightfold) and metastasis suppressor protein-1 (sevenfold); abundance decrease—B-cell CLL/lymphoma 9-like protein (0.02-fold). The fold change of protein abundance is the change in the MG-treated HEK293 cells, with respect to untreated control.

For the cytoplasmic extract, pathway enrichment analysis of proteins with decreased abundance with MG treatment showed over-representation of proteins in the ribosome, spliceosome, RNA transport, and proteasome metabolic pathways (**Table 3**). Sixteen proteins of the spliceosome were detected with MG modification (**Table 4**). For the nuclear extract, proteins decreased were enriched in the major mRNA splicing pathway. In the mitochondrial matrix and intermembrane space and mitochondrial membrane fractions, there were major protein abundance decreases—particularly in the mitochondrial membrane where 116 of 168 proteins detected were decreased in abundance. Pathway enrichment analysis of these proteins revealed enrichment in two key mitochondrial pathways: respiratory electron transport and formation of ATP by chemiosmotic coupling (**Table 3**). There were no MG modifications detected of mitochondrial permeability transition pore (mPTP), respiratory electron transport, or formation of ATP by chemiosmotic coupling component proteins. Overall, the early-stage proteomic response in MG-induced cytotoxicity in HEK293 cells was characterized by a depletion of the RNA splicing proteins and mitochondrial respiratory electron transport chains, suggesting damage of MG to the spliceosome and activation of the mitochondrial apoptotic pathway.

**Table 3:** Pathway enrichment analysis of proteins changed in abundance in HEK293 cells by treatment with methylglyoxal.

Subcellular fraction (abundance change)	Pathway	Count	Fold enrichment	FDR	Fractional abundance	Genes
Cytoplasm (decrease)	Ribosome	51	4.4	4.0 × 10^−18^	0.65 ± 0.17	FAU, MRPL1, MRPL11, MRPL13, MRPL27, MRPL3, MRPS11, MRPS5, NUDT3, RPL10, RPL11, RPL12, RPL13, RPL13A, RPL17, RPL18, RPL21, RPL22, RPL22L1, RPL23, RPL23A, RPL26, RPL29, RPL31, RPL32, RPL34, RPL35, RPL36, RPL38, RPL6, RPL7, RPL8, RPLP2, RPS10, RPS11, RPS13, RPS14, RPS16, RPS18, RPS19, RPS20, RPS25, RPS27, RPS27L, RPS28, RPS29, RPS3, RPS6, RPS8, RPSA, UBA52
	Spliceosome	44	3.9	2.8 × 10^−13^	0.66 ± 0.18	BUD31, CCDC12, CTNNBL1, DDX23, DDX39B, DDX5, DHX15, DHX16, EFTUD2, EIF4A3, HNRNPK, HNRNPM, HNRNPU, LSM7, MAGOH, MAGOHB, PPIE, PPIH, PRPF19, PRPF3, PRPF38B, PRPF4, PRPF40A, PRPF6, RBM17, RBMX, SF3A1, SF3A2, SF3B1, SF3B2, SF3B3, SF3B5, SF3B6, SNRNP40, SNRNP70, SNRNP200, SNW1, SRSF1, SRSF4, SRSF5, SRSF6, THOC3, TRA2B, U2AF2
	RNA transport	46	3.1	2.3 × 10^−10^	0.66 ± 0.14	CYFIP1, DDX39B, EEF1A1, EEF1A2, EIF1, EIF2S1, EIF3C, EIF3CL, EIF3D, EIF3E, EIF3G, EIF3H, EIF3I, EIF3J, EIF4A3, EIF4EBP1, EIF4G1, ELAC2, GEMIN4, GEMIN5, MAGOH, MAGOHB, NMD3, NUP133, NUP205, NUP98, PABPC1, PABPC4, PNN, POP1, RAE1, RAN, RANGAP1, RGPD8, RPP30, SUMO1, SUMO2, SUMO3, TACC3, THOC3, THOC6, TPR, TRNT1, UPF1, UPF3B, XPO5
	Proteasome	16	4.3	9.7 × 10^−5^	0.70 ± 0.06	PSMA3, PSMA4, PSMA6, PSMB1, PSMB2, PSMB6, PSMC5, PSMD3, PSMD4, PSMD7, PSMD8, PSMD11, PSMD12, PSME1, PSME2, PSME3
Nucleus (decrease)	major mRNA splicing	9	12.1	5 × 10^−4^	0.52 ± 0.21	CPSF1, HNRNPUL1, LSM7 MAGOHB, PCBP2, PTBP1, SF3B4, SNRPA1, SNRPD1
Mitochondrial membrane (decrease)	Respiratory electron transport	10	12.1	1.3 × 10^−4^	0.18 ± 0.08	COX5A, COX5B, COX7C, CYCS, NDUFA4, NDUFA5, NDUFB10, SDHB, UQCR10, UQCRB
	Formation of ATP by chemiosmotic coupling	5	28.6	2.8 × 10^−2^	0.24 ± 0.12	ATP5F1A, ATP5F1B, ATP5F1D, ATP5H, ATP5PO

Pathway enrichment analysis is of proteins with decreased abundance in subcellular fractional proteomes of HEK293 cells treated with MG, compared with control. Pathway enrichment analysis was performed using Database for Annotation, Visualization and Integrated Discovery v6.8 (https://david.ncifcrf.gov/) (23), using KEGG and REACTOME. Threshold criterion for significance: FDR <0.05. Fractional abundance is protein abundance in MG-treated cells, compared with untreated control; mean ± SD for the protein count.

**Table 4:** Spliceosome proteins detected with MG-H1 modification in HEK293 cells treated with methylglyoxal *in vitro*.

No	Gene	Name of protein
1	ACIN1	Apoptotic chromatin condensation inducer 1
2	DHX15	DEAH-box helicase 15
3	FUS	RNA-binding protein FUS
4	HNRNPK	Heterogeneous nuclear ribonucleoprotein K
5	HNRNPM	Heterogeneous nuclear ribonucleoprotein M
6	HNRNPU	Heterogeneous nuclear ribonucleoprotein U
7	HSPA1B	Heat shock protein family A member 1B
8	HSPA1L	Heat shock protein family A member like
9	HSPA2	Heat shock protein family A member 2
10	HSPA8	Heat shock protein family A member 8
11	RBM22	Heat shock protein family A member 1B
12	SNRPD2	Small nuclear ribonucleoprotein Sm D2
13	THOC1	THO complex 1
14	TMEM231	Transmembrane protein 231
15	TRA2A	Transformer 2 alpha homolog
16	U2AF1L5	Splicing factor U2AF 35 kDa subunit-like protein

Spliceosome proteins detected with MG-H1 modification of proteomic datasets reported in **Table 2**.

### Association of Methylglyoxal Metabolism With the Spliceosome in Human Tumor Cell Lines and Clinical Human Tumors

**Figure 4:**
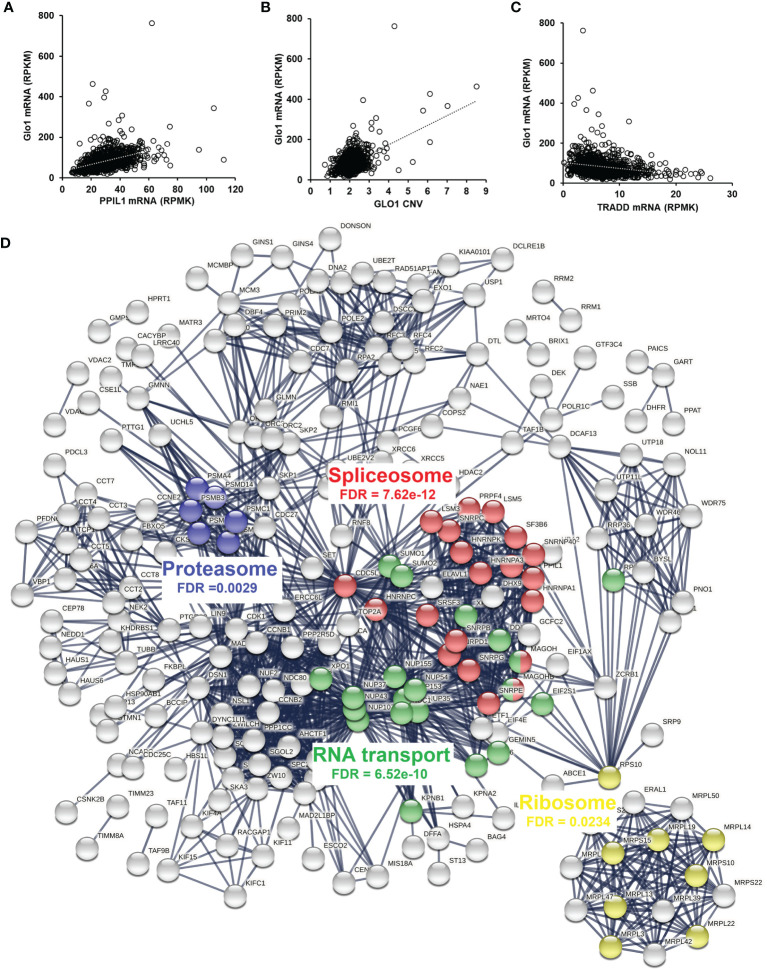
Correlation of glyoxalase 1 expression in human tumor cell lines and clinical human breast cancer with chemotherapy treatment—a negative survival factor. **(A–C)** Correlation of Glo1 expression in the CCLE human tumor cell line database. **(A)** Correlation of Glo1 with PPIL1 (*r* = 0.57). **(B)** Correlation of Glo1 with GLO1 CNV (*r* = 0.53). **(C)** Correlation of Glo1 with TRADD (*r* = −0.35). **(D)** Protein interaction network among genes with expression highly correlated positively with GLO1 in the CCLE human tumor cell database. Several enriched KEGG pathways are highlighted and their significance indicated (false discovery rate (FDR)). For clarity, only the largest connected component is shown.

Having discovered the spliceosome as a target of MG-induced toxicity, we assessed if expression of Glo1 may be linked to protection of the spliceosome in human tumor cell lines and clinical human tumors. An indication of this would be positive correlation of expression of spliceosome proteins with expression of Glo1. To explore this in human tumor cell lines, we accessed RNA-seq data from the CCLE—a database of gene expression and gene copy number estimates for 1,040 human tumor cell lines (https://portals.broadinstitute.org/ccle). Data were available from 1,010 cell lines of different tumor types. Correlation analysis revealed 4,021 genes correlating significantly with Glo1: 3,032 correlated positively with Glo1 with correlation coefficients (*r*) in the range 0.134–0.568 and 989 genes correlated negatively with Glo1 with *r* in the range −0.535 to −0.134 (**Supplementary Table S12**). Two of the top 4 genes rank ordered by *r*-value were genes of the spliceosome: PPIL1 (first, *r* = 0.57) (**Figure 4A**) and CDC5L (fourth, *r* = 0.52). There was also a strong positive correlation of Glo1 mRNA copy number with GLO1 gene copy number (*r* = 0.53), suggesting that increased GLO1 copy number may contribute to increased Glo1 mRNA (**Figure 4B**). Genes with expression correlating positively with Glo1 were analyzed for pathway enrichment analysis, limiting genes for which correlation coefficient *r* ^2^ ≥ 0.10, thereby accounting for ≥10% variation in Glo1 expression. For the 340 genes submitted for analysis, pathway enrichment analysis revealed four pathways: spliceosome, RNA transport, cell cycle, and DNA replication (**Table 5**). This is also illustrated in an interaction network diagram (**Figure 4D**). Increased expression of Glo1 may provide a protective shield of the spliceosome against MG glycation permissive for tumor growth. DNA replication in the cell cycle is dependent on pre-mRNA splicing and accurate alternative splicing (31). Analysis of structural domains enriched in these proteins showed highest enrichment of chaperonin TCP-1, conserved site (**Table 6**). This was previously found to be enriched in proteins susceptible to modification by MG (18).

**Table 5:** Pathway enrichment of gene expression correlating positively with glyoxalase 1 expression in tumor cell lines of the CCLE.

Pathway	Count	Fold enrichment	FDR	Genes
Spliceosome	20	7.0	3.4 × 10^−9^	CDC5L, HNRNPA1, HNRNPA3, HNRNPC, **HNRNPK**, LSM2, LSM3, LSM5, **MAGOH**, **MAGOHB**, PPIL1, **PRPF4**, RBMXL1, SNRNP40, SNRPB, SNRPC, SNRPD1, SNRPE, SNRPG, SRSF3
RNA transport	22	6.0	3.4 × 10^−9^	DDX20, EIF1AX, **EIF2S1**, EIF4E, **GEMIN5**, GEMIN6, KPNB1, **MAGOH**, **MAGOHB**, NDC1, NUP35, NUP37, NUP43, NUP54, NUP107, NUP153, NUP155, RPP40, **SUMO1**, **SUMO2**, XPO1, **XPO5**
Cell cycle	19	7.2	3.4 × 10^−9^	CCNB1, CCNB2, CCNE2, CDC25C, CDC27, CDC7, CDK1, DBF4, HDAC2, MAD2L1, MCM3, ORC2, ORC3, ORC4, PTTG1, SKP1, SKP2, PRIM2, TTK
DNA replication	10	12.7	1.2 × 10^−6^	DNA2, MCM3, POLA1, POLE2, PRIM2, RFC2, RFC3, RFC4, RFC5, RPA2
Proteasome	6	6.4	0.037	PSMD12, PSMA4, PSMD14, PSMB3, PSMC1, PSMB1

Pathway enrichment analysis was performed for 340 genes correlating positively with Glo1 RNA copy number in the CCLE database with r^2^ ≥ 0.1 using the KEGG database; data from 1,010 tumor cell lines for 10,758 genes, applying a Bonferroni correction of 10,758 (**Supplementary Table S12**). Statistical threshold criterion was FDR <0.05. Genes unrecognized in KEGG were pseudogene (2), antisense RNA (7), microRNA (2), long noncoding RNA (4), other intronic RNA (1), and uncharacterized proteins (1). Genes highlighted in bold are those encoding for proteins that were decreased in the early-stage proteomic response to MG-induced cytotoxicity.

**Table 6:** Pathway enrichment of protein domains in genes with expression correlating positively with glyoxalase 1 expression in tumor cell lines of the CCLE.

Protein domain	Count	Bonferroni *p*-value	FDR	Genes
Chaperonin TCP-1, conserved site	8	7.1 × 10^−9^	1.8 × 10^−8^	CCT2, CCT3, CCT4, CCT5, CCT6A, CCT7, CCT8, TCP1
Ribonucleoprotein LSM domain	8	1.9 × 10^−5^	4.8 × 10^−5^	LSM2, LSM3, LSM5, SNRPB, SNRPD1, SNRPD2, SNRPE, SNRPG
Importin-beta, N-terminal	6	4.0 × 10^−3^	9.8 × 10^−3^	CSE1L, IPO11, IPO7, KPNB1, XPO1, XPO5

Genes with expression correlating positively with Glo1 with correlation coefficient r^2^ ≥ 0.10 were analyzed for protein domain enrichment analysis. For the 340 genes submitted for analysis, protein domain enrichment analysis was performed by INTERPRO in the Database for Annotation, Visualization and Integrated Discovery v6.8 (https://david.ncifcrf.gov/) (23). Threshold criterion for significance: FDR <0.05.

For negative correlation with Glo1, there were only eight genes with *r* ^2^ ≥ 0.10. These were three pseudogenes and two long noncoding RNAs; *N*-acetylglucosamine-1-phosphate transferase subunit gamma (GNPTG)—part of a complex targeting lysosomal hydrolases to the lysosome and Tapasin (TAPBL) which mediates binding of newly assembled major histocompatibility complex class I molecules and the transporter associated with antigen processing (TAP), both *r* ^2^ = 0.11; and tumor necrosis factor receptor type 1-associated death domain protein (TRADD)—an adaptor protein involved in receptor-mediated apoptosis, *r* = −0.35 (**Figure 4C**). This supports the link of increased Glo1 expression with decreased risk of apoptosis, through decreased risk of MG-induced apoptosis.

For clinical translation, we performed correlation analysis of Glo1 expression with PPIL1, CDC5L, and TRADD in a pan cancer database (7,489 multiple types of human tumor; RNA seq) and breast cancer (*n* = 4,939, gene chip) of the KM Plotter database—a compendium of gene expression and survival data of cancer patients receiving chemotherapy and other treatments. Correlation analysis with Glo1 expression gave the following: pan cancer—PPIL1, *r* = 0.59 and CDC5L, *r* = 0.58 (*p* < 1 × 10^−6^) and TRADD, *r* = −0.17 (*p* = 5 × 10^−4^); and for breast cancer—PPIL1, *r* = 0.55; CDC5L, *r* = 0.26 and TRADD, *r* = −0.20 (*p* < 1 × 10^−6^). This indicates that there was a similar correlation of Glo1 expression in human clinical tumors as in the human tumor cell lines of the CCLE.

### Glyoxalase 1 Expression as a Risk Predictor of Cancer Patient Survival With Chemotherapy Treatment

**Figure 5:**
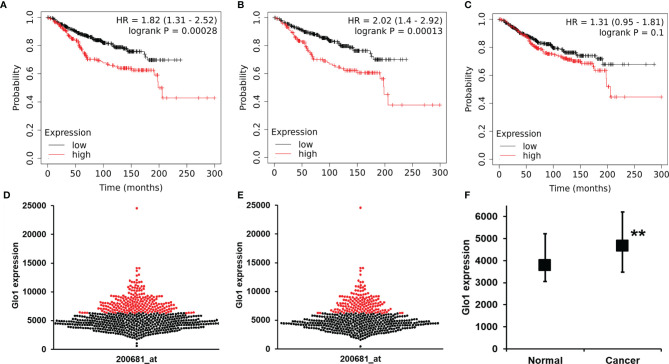
Glyoxalase 1 expression is a negative survival factor in clinical human breast cancer with chemotherapy treatment. Association of baseline Glo1 expression with overall survival in patients with breast cancer treated with chemotherapy (from the KM Plotter database). Glo1 expression in breast cancers of all genotypes: **(A)** Kapan-Meier survival analysis and **(D)** butterfly plot of breast cancers of low (black dots) and high (red dots) Glo1 expression (*n* = 683). Glo1 expression in patients with HER2-negative tumors: **(B)** Kapan-Meier survival analysis and **(E)** butterfly plot of breast cancers of low (black dots) and high (red dots) Glo1 expression (*n* = 531). **(C)** Kapan-Meier survival analysis for Glo2 expression in breast cancers of all genotypes (*n* = 683). **(F)** Glo1 expression in normal breast tissue and breast cancers. Data are median (lower – upper quartile); normal, n = 3778, cancer, n = 4667. Significance: **P < 0.01; Mann-Whitney U test. For the low and high gene expression cutoff, the “Autoselect best cutoff” feature in the KM Plotter was used. This computes survival outcomes for all possible cutoff values between lower and upper quartiles of Glo1 expression and selects the cutoff for FDR below the threshold value (0.05) and minimum *p*-value (24).

To explore the clinical relevance of baseline Glo1 expression in clinical cancer chemotherapy, we investigated association of Glo1 expression with cancer patient survival receiving chemotherapy in the KM Plotter database. For chemotherapy (any), analysis had adequate statistical power for Glo1 expression and patient survival for breast cancer without classification of tumor stage, grade, type, and genotype. The one exception was for analysis of the HER2-negative genotype. For breast cancer patients, high expression of Glo1 was associated with poor survival, with HR = 1.82 (logrank *p* < 0.001, *n* = 683) (**Figures 5A, D**). This effect was also present in HER2-negative breast cancer. For HER2-negative breast cancer, high expression of Glo1 was associated with poor survival, with HR = 2.02 (logrank *p* < 0.001, *n* = 531) (**Figures 5B, E**). The upper quartile survival was low Glo1 expression, 173.7 months, and high Glo1 expression, 63.5 months in both survival analysis datasets; 64% decreased survival. In contrast, there was no significant association of Glo2 (gene HAGH) expression with breast cancer patient survival (**Figure 5C**). For the entire dataset of breast cancer patients in the KM Plotter database, the expression of Glo1 was increased in cancer compared with nonmalignant breast tissue (**Figure 5F**).

## Discussion

Overexpression of Glo1 suppressed the antiproliferative activity of multiple classes of antitumor drugs. Drug-induced increase of cellular MG to cytotoxic levels contributed to the mechanism of action of antitumor drugs, linked to increased formation of MG by off-target drug-induced increased glycolysis. Alkylating agents such as mechlorethamine and topoisomerase inhibitors lead to decreased cellular NAD^+^ in early-stage of treatment, decreasing glycolysis at the glyceraldehyde-3-phosphate dehydrogenase-catalyzed step and consequently increasing cellular GA3P and DHAP and formation of MG (32–34). Doxorubicin also increases glucose metabolism by increasing expression of glucose transporter GLUT1 and hexokinase-2 (35). Paclitaxel stabilizes microtubules, decreasing free tubulin concentration; the latter increasing mitochondrial voltage-dependent anion channel (VDAC) activity and thereby *in situ* activity of hexokinase and glycolysis (36). Methotrexate activates AMPK and thereby stimulates glycolysis by activating phosphorylation of 6-phosphofructo-2-kinase/fructose-2,6-bisphosphatase (37). Increased formation of MG by antitumor agents is likely most marked in S phase of the cell cycle when expression of glycolysis enzymes is highest (38). Indeed, early studies of MG-induced apoptosis suggested cell death occurs in this phase (39). The three- to fivefold increase of MG concentration in the total cell population induced by anticancer drugs may underestimate that of S phase cells, accounting for *ca.* 20% of the cell population (40).

Although anticancer drug-induced increase of MG to cytotoxic levels contributing to the antiproliferative mechanism of action likely explains the susceptibility of the anticancer drugs to Glo1-mediated MDR, the increased cellular concentration of MG assayed after exposure of HEK293 cells to anticancer drugs for 3 h was not strongly correlated to drug-associated fold of Glo1-linked MDR. The latter is expected to correlate with peak MG concentration induced by drug treatment. Increase in MG concentration in response to anticancer drug treatment is expected to increase rapidly and maximize concomitant with increase in the concentration of cellular triosephosphates, as found previously at 3 h for mechlorethamine-treated leukemia L1220 cells (33). However, the time of peak MG concentration likely varies, and so maximal drug-induced MG concentration was not captured herein. Rather, herein, we identified that many anticancer drugs increase cellular MG to cytotoxic levels.

There was an exception to Glo1 expression-linked MDR: enhanced antiproliferative and cytotoxic activity of cisplatin by overexpression of Glo1. Cisplatin is a DNA-alkylating agent, mainly binding and crosslinking N_7_ sites of deoxyguanosine (dG), intrastrand crosslink, cis-Pt(NH3)2d (pGpG) (41). MG is a major precursor of endogenous DNA modification *in vivo* forming imidazopurinone MGdG, in slow dynamic equilibrium with MG; degradation half-life 12 h at pH 7.4 and 37°C. Cellular DNA content of MGdG is *ca.* 1:20,000 dG. Cisplatin may interact favorably with the 6,7-dihydro-6,7-dihydroxy-6/7-methylimidazo moiety of MGdG and thereby be inactivated, explaining why increase of Glo1 with decrease of MG and MGdG increases the cytotoxicity of cisplatin.

A feature of MG metabolism in HEK293 cells was the impact of hypoxia, producing increased flux through anaerobic glycolysis with a twofold increased formation of MG and threefold decrease in activity of Glo1. These responses synergize to increase cellular MG and likely contribute to the 60-fold increased potency of BBGD for induction of cytotoxicity in HEK293 cells with an atmosphere of 3% oxygen versus 20% oxygen. Cell permeable Glo1 inhibitors thereby may have greater potency in hypoxic than normoxic tumors *in vivo*, assuming the downregulation of Glo1 by hypoxia is maintained. In some instances, however, hypoxia conditioning of tumors leads to dysregulation and increased Glo1 expression in hypoxia (42). From the studies herein, dysregulation of Glo1 in tumor hypoxia may contribute to MDR in cancer chemotherapy and increased tumor survival.

Herein, we provided the first examination of the early-stage proteomic response to MG-induced cytotoxicity, finding protein abundance decrease enriched in pathways involving ribosome, spliceosome, RNA, and proteasome pathways. Ribosomal pathways of protein synthesis contain multiple proteins susceptible to MG modification, as found in studies of cytoplasmic protein extracts of endothelial cells (18). Ribosomal proteins may suffer preferential modification by MG and thereafter increased degradation. This study is the first to implicate the spliceosome in the mechanism of MG cytotoxicity. There was a decreased abundance of 95 ribosomal and spliceosomal proteins in MG-induced cytotoxicity (**Table 3**). Dysfunction of the spliceosome is induced by multiple classes of anticancer drugs (43). There was a 48% decrease of major pathway of RNA splicing in nuclear extracts. Further associations between MG metabolism and the spliceosome were the positive correlation of Glo1 expression with expression of spliceosomal genes in human tumor cell lines and clinical human tumors and MG modification detected on 16 spliceosome pathway proteins. The arginine-rich domains of serine/arginine-rich splicing factors, SRSFs 1–12, may be targets in MG-induced cytotoxicity. SRSF1, SRSF4, SRSF5, and SRSF6 were decreased by MG-induced toxicity in HEK393 cells herein. Dysregulation of these splicing factors has been implicated in cancers of the breast, lung, and colon; melanoma; and acute myeloid leukemia (44). Activity of Glo1 inhibitor prodrugs may deserve investigation against these tumor types.

In selection of the duration of exposure of HEK293 cells to MG to capture the proteomic signature for commitment to apoptosis, we determined the minimum duration of incubation of HEK293 cells with MG for decrease in cell viability. We incubated cells with MG for varying periods, washed out residual MG and then continued incubations for 48 h for apoptosis to fully develop and decrease in viable cell number. For treatment with the GC_50_ concentration of MG, the minimum exposure time to decrease cell viability by 50%—complete response—was incubation for 12 h. To capture cells at the midpoint of induction of apoptosis, we therefore incubated cells with MG for 6 h (**Figure 3I**). Analysis of total cell protein glycation by MG at 6 h also showed that this was the time point of maximum MG-derived glycation adduct, MG-H1, content which facilitated detection of MG-modified protein targets. MG-induced cytotoxicity was also characterized by a profound loss of proteins of the mitochondrial chemiosmotic coupling of ATP formation and respiratory electron transport, disabling cellular metabolic energy and respiration pathways. It has been proposed that MG activates the intrinsic apoptotic pathway by modification of the mPTP complex of proteins, a high conductance channel in mitochondria (45, 46). MG modification was not detected on any mPTP proteins herein, and these proteins were not highly correlated with Glo1 in the CCLE and clinical tumor gene expression. This suggests that the loss of mitochondrial proteins may be secondary to dysregulation of the spliceosome in MG-induced apoptosis (47).

The clinical relevance of the metabolism of MG was investigated in cancer patients treated with chemotherapy. We found a marked, 64% decreased overall survival with high expression of Glo1. This is the first time clinical expression of Glo1 in tumors has been linked to survival in clinical cancer chemotherapy. Removal of MG by Glo1 was key to this effect as there was no similar association with expression of Glo2 (gene HAGH). The clinical endpoint used herein, overall patient survival, is a well-defined and robust endpoint. Our findings implicate expression of Glo1 in human breast cancer as a factor with major impact on patient survival. The effect was found in a cohort that had mainly HER2-negative genotype, representing *ca.* 60% breast cancers with often poor cancer chemotherapy outcomes (48). Application of Glo1 inhibitors for relief of Glo1-linked MDR may be a beneficial adjunct treatment for breast cancer treatment by chemotherapy.

Previous studies have shown that cytotoxicity of antitumor drugs in drug-resistant cell lines was enhanced by cotreatment with BBGD (49) and treatment of tumor-bearing mice with BBGD decreased tumor growth, including tumors difficult to treat with current clinical antitumor drugs (8, 49–51). Adjunct chemotherapy with Glo1 inhibitor may improve treatment outcomes in clinical cancer chemotherapy, particularly where high expression of Glo1 is a risk predictor of poor survival outcome.

We conclude that MG-mediated cytotoxicity is a common contributor to the cancer chemotherapeutic response, stimulated by off-target effect of anticancer drugs on glycolysis. Enhanced in hypoxia relevant to clinical tumors, its clinical impact has likely been hitherto under-appreciated. MG-mediated cytotoxicity involves activation of the intrinsic apoptotic pathway with targeting of the spliceosome for MG modification. Conversely, Glo1 is a common mediator of MDR in cancer chemotherapy. Glo1-mediated MDR has a major role in poor survival outcomes in breast cancer which may be countered by clinical development of Glo1 inhibitors. Glo1 is an established druggable target (8, 49, 51). Future clinical translation of cell permeable Glo1 inhibitors as adjunct chemotherapy may improve overall survival in breast cancer and other cancers with high Glo1 expression.

## Data Availability Statement

The datasets presented in this study can be found in online repositories. CCLE and Kaplan-Meier Survival Analysis Database repositories are described in the Methods Section of this article. The proteomic data presented in the study are deposited in the PRIDE Proteome Xchange repository, accession number PXD029315. Other raw data supporting the conclusions of this article will be made available by the authors, without undue reservation.

## Ethics Statement

All methods were carried out in accordance with relevant guidelines and regulations and all experimental protocols were approved by University of Warwick Genetic Modification & Biosafety Committee (Project no. 305).

## Author Contributions

HA cultured HEK293 cells, prepared and propagated plasmids, prepared stable transfectant cell lines, and performed metabolite and drug treatment studies. MA cultured HEK293 cells and studied the proteomic response to MG-induced cytotoxicity. AF performed pathway enrichment analysis. MX provided technical guidance and support to HA and MA. PJT assisted with MG analysis. NR and PJT designed and supervised the studies, contributed to the data analysis, and wrote the manuscript. All authors read and approved the published version of the manuscript.

## Funding

MA thanks Bisha University for research funding, Project UB-39). PT thanks the Qatar Foundation for funding his research program (project code QB-14). NR thanks Qatar University for funding her research. The findings achieved herein are solely the responsibility of the authors.

## Conflict of Interest

The authors declare that the research was conducted in the absence of any commercial or financial relationships that could be construed as a potential conflict of interest.

## Publisher’s Note

All claims expressed in this article are solely those of the authors and do not necessarily represent those of their affiliated organizations, or those of the publisher, the editors and the reviewers. Any product that may be evaluated in this article, or claim that may be made by its manufacturer, is not guaranteed or endorsed by the publisher.
