# Supplementary material for: Studies of Glyoxalase 1-Linked Multidrug Resistance Reveal Glycolysis-Derived Reactive Metabolite, Methylglyoxal, Is a Common Contributor in Cancer Chemotherapy Targeting the Spliceosome
Source: Front Oncol. 2021 Nov 1;11:748698. doi: 10.3389/fonc.2021.748698 (PMC8591171; doi:10.3389/fonc.2021.748698)
Supplement: Supplementary file 1 [file DataSheet_1.pdf]

Studies of glyoxalase 1-linked multidrug resistance reveal glycolysis-derived reactive metabolite, methylglyoxal, is a common contributor in cancer chemotherapy targeting the spliceosome.

Muhanad Alhujaily et al.

Figure S1. The effect of the anti-cancer drugs on Glo1 activity of HEK293 cell lysates. Anticancer drugs were added to diluted cytosolic extracts of HEK293 cells and activity of glyoxalase 1 assayed as described [1]. Data are mean ± SD (n = 4 for control and n = 3 for drug treated). Significance: * and **, P<0.05 and P<0.05, with respect to control; *Student's t-test*. Key for drug concentrations: , + GC_{50}; , + 2 x GC_{50}; and , + 5 x GC_{50}.

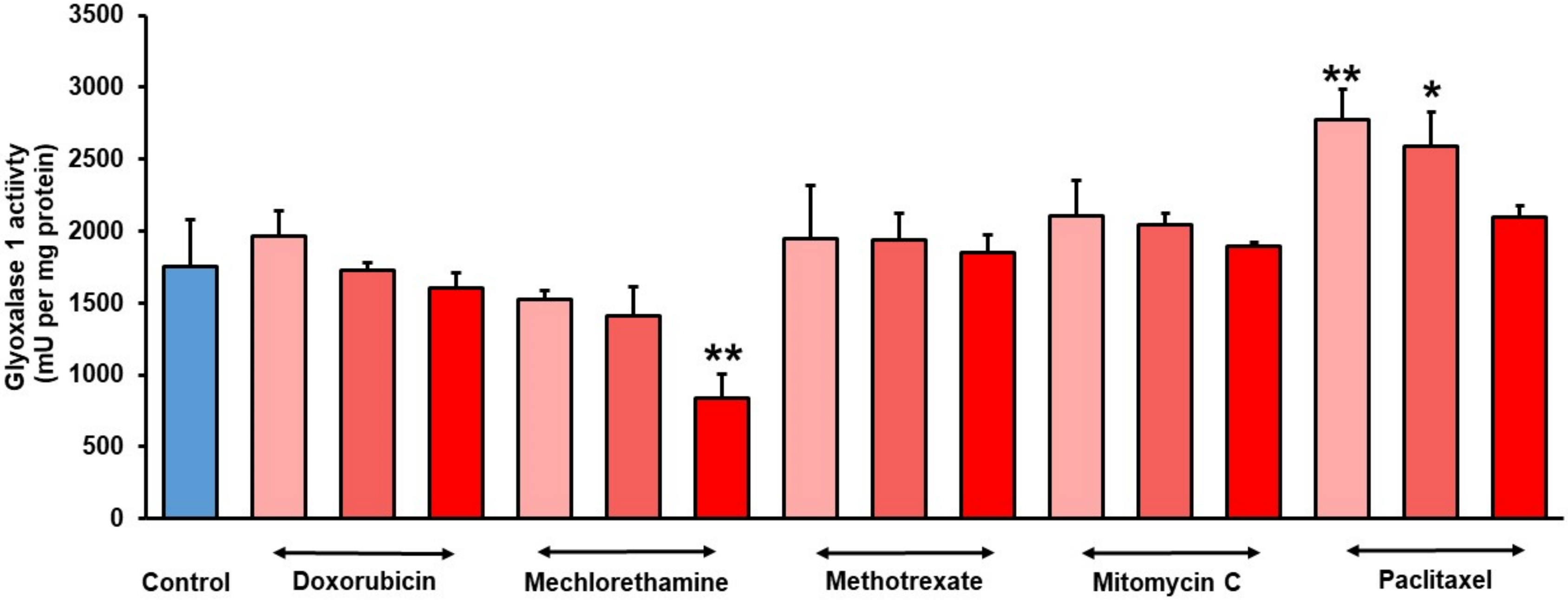

4,

Studies of glyoxalase 1-linked multidrug resistance reveal glycolysis-derived reactive metabolite, methylglyoxal, is a common contributor in cancer chemotherapy targeting the spliceosome.

Muhanad Alhujaily et al.

Table S1. Proteins in the cytoplasmic extract increased in abundance by treatment with methylglyoxal.

No	Gene	Name of protein	Fold change
1	TMEM63B	CSC1-like protein 2	30.5
2	MEGF11	Multiple epidermal growth factor-like domains protein 11	14.0
3	TMEM259	Membralin	8.2
4	TSTA3	GDP-L-fucose synthase	5.7
5	CORO1C	Coronin	5.2
6	EIF4E	Eukaryotic translation initiation factor 4E	5.1
7	LSM7	U6 snRNA-associated Sm-like protein LSm7	4.9
8	TMEM132B	Transmembrane protein 132B	4.8
9	NIN	Ninein	4.8
10	UBL5	Ubiquitin-like protein 5	4.4
11	NLRP2	NACHT, LRR and PYD domains-containing protein 2	4.1
12	TUB	Tubby-like protein	3.9
13	TSPYL2	Testis-specific Y-encoded-like protein 2	3.8
14	TRIM52	Tripartite motif-containing protein 52	3.6
15	CHMP2A	Charged multivesicular body protein 2a	3.5
16	GC	Vitamin D-binding protein	3.5
17	C5	Complement C5	3.5
18	GPR119	Glucose-dependent insulinotropic receptor	3.4
19	KRT9	Keratin, type I cytoskeletal 9	3.3
20	ABHD12B	Protein ABHD12B	3.2
21	CACNA1H	Voltage-dependent T-type calcium channel subunit alpha-1H	3.2
22	SV2B	Synaptic vesicle glycoprotein 2B	3.2
23	PHF5A	PHD finger-like domain-containing protein 5A	3.0
24	CTSB CPSB	Cathepsin B	2.9
25	NUP50	Nuclear pore complex protein Nup50	2.8
26		Uncharacterized protein	2.8
27	THRA	Thyroid hormone receptor alpha	2.7
28	KIAA0355	Uncharacterized protein KIAA0355	2.6
29	COQ9	Ubiquinone biosynthesis protein COQ9, mitochondrial	2.6
30	FAAP20	Fanconi anemia core complex-associated protein 20	2.6
31	REXO2	Oligoribonuclease, mitochondrial	2.4
32	CIITA	MHC class II transactivator	2.4
33	DROSHA	Ribonuclease 3	2.4
34	VAT1	Synaptic vesicle membrane protein VAT-1 homolog	2.3
35	MZT2A	Mitotic-spindle organizing protein 2A	2.3
36	PPARD	Peroxisome proliferator-activated receptor delta	2.2
37	CKS2	Cyclin-dependent kinases regulatory subunit 2	2.2
38	MRPL1	39S ribosomal protein L1, mitochondrial	2.1
39	ZNF568	Zinc finger protein 568	2.1
40	BRCA2	Breast cancer type 2 susceptibility protein	2.1
41	MXRA7	HBV PreS1-transactivated protein 1	2.1
42	HIBADH	3-hydroxyisobutyrate dehydrogenase, mitochondrial	2.1
43	PTPRN2	Receptor-type tyrosine-protein phosphatase N2	2.1
44	SLC25A37	Mitoferrin-1	2.1
45	METAP1	Methionine aminopeptidase 1	2.1
46	SCAPER	S phase cyclin A-associated protein in the ER	2.1
47	SDHA	Succinate dehydrogenase [ubiquinone] flavoprotein subunit, mitochondrial	2.1
48	GGCT	Gamma-glutamylcyclotransferase	2.1
49	NIPBL	Nipped-B-like protein	2.0

50	TUBGCP6	Gamma-tubulin complex component 6	2.0
51	UNG	Uracil-DNA glycosylase	2.0
52	COL4A4	Collagen alpha-4	2.0
53	CLN5	Ceroid-lipofuscinosis neuronal protein 5	2.0
54	MACF1	Microtubule-actin cross-linking factor 1, isoforms 1/2/3/5	2.0
55	CRABP2	Cellular retinoic acid-binding protein 2	2.0
56	TSFM	Elongation factor Ts, mitochondrial	2.0
57	COL27A1	Collagen alpha-1	2.0
58	MECP2	Methyl-CpG-binding protein 2	1.9
59	CCNDBP1	Cyclin-D1-binding protein 1	1.9
60	PLCB1	1-phosphatidylinositol 4,5-bisphosphate phosphodiesterase	1.9
61	SERPINA7	Thyroxine-binding globulin	1.9
62	ZSWIM9	Uncharacterized protein ZSWIM9	1.9
63	CCDC8	Coiled-coil domain-containing protein 8	1.9
64	IWS1	Protein IWS1 homolog	1.9
65	SPTA1	Spectrin alpha chain, erythrocytic 1	1.8
66	SEMA4B	Semaphorin-4B	1.8
67	CPEB1	Cytoplasmic polyadenylation element binding protein 1, isoform CRA_c	1.8
68	CRELD2	Cysteine-rich with EGF-like domain protein 2	1.8
69	UNC13B	Protein unc-13 homolog B	1.8
70	SEPTIN9	Septin 9	1.8
71	A2M	Alpha-2-macroglobulin	1.8
72	CABIN1	Calcineurin-binding protein cabin-1	1.8
73	ZNF622	Zinc finger protein 622	1.8
74	YARS2	Tyrosine-tRNA ligase	1.8
75	MBOAT7	Lysophospholipid acyltransferase 7	1.8
76	LRBA	Lipopolysaccharide-responsive and beige-like anchor protein	1.7
77	SLC6A9	Transporter	1.7
78	ARMH3	Armadillo-like helical domain-containing protein 3	1.7
79	NPEPPS	Puromycin-sensitive aminopeptidase	1.7
80	MYEF2	Myelin expression factor 2	1.7
81	TMPRSS11B	Transmembrane protease serine 11B	1.7
82	MDC1	Mediator of DNA damage checkpoint protein 1	1.6
83	UBQLN1	Ubiquilin-1	1.6
84	MUC20	Mucin-20	1.6
85	SPRED2	Sprouty-related, EVH1 domain-containing protein 2	1.6
86	BUD31	Protein BUD31 homolog	1.6
87	SAMD11	Sterile alpha motif domain-containing protein 11	1.6
88	CEP250	Centrosome-associated protein CEP250	1.6
89	WDR33	pre-mRNA 3' end processing protein WDR33	1.6
90	CCT7	T-complex protein 1 subunit eta	1.5
91	SNRPB2	U2 small nuclear ribonucleoprotein B''	1.5
92	SDHB	Succinate dehydrogenase [ubiquinone] iron-sulfur subunit, mitochondrial	1.5
93	PPP4R1L	Putative serine/threonine-protein phosphatase 4 regulatory subunit 1-like	1.5
94	ALK	Tyrosine-protein kinase receptor	1.5
95	SNTG2	Gamma-2-syntrophin	1.5
96	C20orf27	UPF0687 protein C20orf27	1.5
97	EIPR1	EARP and GARP complex-interacting protein 1	1.5
98	DAP3	28S ribosomal protein S29, mitochondrial	1.5
99	WDR61	WD repeat-containing protein 61	1.5
100	DIS3L2	DIS3-like exonuclease 2	1.4
101	ALDH3A2	Fatty aldehyde dehydrogenase	1.4
102	ETS1	Protein C-ets-1	1.4
103	DAAM2	Disheveled-associated activator of morphogenesis 2	1.4
104	WNK3	Serine/threonine-protein kinase WNK3	1.4

105	EDC3	Enhancer of mRNA-decapping protein 3	1.4
106	FMN2	Formin-2	1.4
107	GRD1	Glutathione reductase, mitochondrial	1.4
108	FAM27E3	Protein FAM27E3	1.3
109	MRPS9	28S ribosomal protein S9, mitochondrial	1.3
110	MBNL1	Muscle blind-like protein 1	1.3
111	LMNA	Prelamin-A/C [Cleaved into: Lamin-A/C	1.3
112	WBP11	WW domain-binding protein 11	1.3
113	SMG6	Telomerase-binding protein EST1A	1.3
114	LMNA	Prelamin-A/C	1.3
115	FABP5	Fatty acid-binding protein 5	1.2
116	RBM14	RNA-binding protein 14	1.2
117	POM121C	Nuclear envelope pore membrane protein POM 121C	1.2
118	NFU1	NFU1 iron-sulfur cluster scaffold homolog, mitochondrial	1.2
119	AKR1B1	Aldo-keto reductase family 1 member B1	1.2
120	TRA2B	Transformer-2 protein homolog beta	1.2
121	NASP	Nuclear autoantigenic sperm protein	1.2

Studies of glyoxalase 1-linked multidrug resistance reveal glycolysis-derived reactive metabolite, methylglyoxal, is a common contributor in cancer chemotherapy targeting the spliceosome.

Muhanad Alhujaily et al.

Table S2. Proteins in the cytoplasmic extract decreased in abundance by treatment with methylglyoxal.

No	Gene	Name of protein	Fold change
1	HNRNPK	Heterogeneous nuclear ribonucleoprotein K	0.91
2	GDI1	Rab GDP dissociation inhibitor alpha	0.90
3	YWHAZ	14-3-3 protein zeta/delta	0.89
4	GDI1	Rab GDP dissociation inhibitor	0.89
5	DDX21	Nucleolar RNA helicase 2	0.89
6	HSP90B1	Endoplasmin	0.89
7	GDI2	Rab GDP dissociation inhibitor	0.88
8	NPM1	Nucleophosmin	0.88
9	NASP	Nuclear autoantigenic sperm protein	0.88
10	SERBP1	Plasminogen activator inhibitor 1 RNA-binding protein	0.88
11	RTCB	tRNA-splicing ligase RtcB homolog	0.88
12	ACTN4	Alpha-actinin-4	0.88
13	HSPE1	10 kDa heat shock protein, mitochondrial	0.87
14	SRSF1	Serine/arginine-rich-splicing factor 1	0.87
15	MYH9	Myosin-9	0.87
16	RBM39	RNA-binding protein 39	0.87
17	EIF3J	Eukaryotic translation initiation factor 3 subunit J	0.87
18	LMNB1	Lamin-B1	0.87
19	EIF4EBP1	Eukaryotic translation initiation factor 4E-binding protein 1	0.87
20	CALU	Calumenin	0.87
21	SEPT11	Septin-11	0.87
22	SRSF6	Serine/arginine-rich splicing factor 6	0.86
23	ARHGDIA	Rho GDP-dissociation inhibitor 1	0.86
24	FUS	RNA-binding protein FUS	0.86
25	CNPY2	Protein canopy homolog 2	0.86
26	TPR	Nucleoprotein TPR	0.86
27	RAB4A	Ras-related protein Rab-4A	0.86
28	RAN	GTP-binding nuclear protein Ran	0.86
29	SGTA	Small glutamine-rich tetratricopeptide repeat-containing protein alpha	0.86
30	RRP1B	Ribosomal RNA processing protein 1 homolog B	0.86
31	PCBD1	Pterin-4-alpha-carbinolamine dehydratase	0.86
32	RPS14	40S ribosomal protein S14	0.86
33	FTSJ3	pre-rRNA processing protein FTSJ3	0.85
34	RPS10P5	Putative 40S ribosomal protein S10-like	0.85
35	RPLP2	60S acidic ribosomal protein P2	0.85
36	H2AFY	Core histone macro-H2A.1	0.85
37	TPP2	Tripeptidyl-peptidase 2	0.85
38	PPA1	Inorganic pyrophosphatase	0.85
39	RCC2	Protein RCC2	0.85
40	CYFIP1	Cytoplasmic FMR1-interacting protein 1	0.85
41	DYNC1I2	Cytoplasmic dynein 1 intermediate chain 2	0.85
42	TPD52L2	Tumor protein D54	0.85
43	SUMO2	Small ubiquitin-related modifier 2	0.85
44	METAP2	Methionine aminopeptidase 2	0.85
45	PFN1	Profilin	0.85
46	LMNB1	Lamin B1, isoform CRA_a	0.85
47	TMEM189-UBI	HCG2044781	0.84
48	TXNDC17	Thioredoxin domain-containing protein 17	0.84
49	SPTAN1	Spectrin alpha chain, non-erythrocytic 1	0.84

50	MCM6	DNA replication licensing factor MCM6	0.84
51	HSP90AB4P	Putative heat shock protein HSP 90-beta 4	0.84
52	PKM	Pyruvate kinase	0.84
53	STRBP	Spermatid perinuclear RNA-binding protein	0.84
54	ZCCHC2	Zinc finger CCHC domain-containing protein 2	0.84
55	NPEPPSL1	Puromycin-sensitive aminopeptidase-like protein	0.84
56	COPS3	COP9 signalosome complex subunit 3	0.84
57	RPS6	40S ribosomal protein S6	0.84
58	MTHFD1	C-1-tetrahydrofolate synthase, cytoplasmic	0.83
59	RPL23A	60S ribosomal protein L23a	0.83
60	MAP4	Microtubule-associated protein	0.83
61	GDI2	Rab GDP dissociation inhibitor beta	0.83
62	ILF3	Interleukin enhancer-binding factor 3	0.83
63	KHSRP	Far upstream element-binding protein 2	0.83
64	LARS	Leucine--tRNA ligase, cytoplasmic	0.83
65	SUMO3	Small ubiquitin-related modifier 3	0.83
66	PDIA6	Protein disulfide-isomerase A6	0.83
67	PLS3	Plastin-3	0.83
68	PDHB	Pyruvate dehydrogenase E1 component subunit beta, mitochondrial	0.83
69	GSPT1	Eukaryotic peptide chain release factor GTP-binding subunit ERF3A	0.83
70	TBCA	Tubulin-specific chaperone A	0.83
71	SF3A1	Splicing factor 3A subunit 1	0.82
72	PTRHD1	Putative peptidyl-tRNA hydrolase PTRHD1	0.82
73	FKBP4	Peptidyl-prolyl cis-trans isomerase FKBP4	0.82
74	HSP90AB1	Heat shock protein HSP 90-beta	0.82
75	POLR2C	DNA-directed RNA polymerase II subunit RPB3	0.82
76	U2AF2	Splicing factor U2AF 65 kDa subunit	0.82
77	MCM3	DNA replication licensing factor MCM3	0.82
78	SLC1A5	Neutral amino acid transporter B(0)	0.82
79	RPL10	60S ribosomal protein L10	0.82
80	DHX15	Pre-mRNA-splicing factor ATP-dependent RNA helicase DHX15	0.82
81	PRPS1L1	Ribose-phosphate pyrophosphokinase 3	0.82
82	RPSA	40S ribosomal protein SA	0.82
83	CAND1	Cullin-associated NEDD8-dissociated protein 1	0.82
84	DDX39A	ATP-dependent RNA helicase DDX39A	0.82
85	EEF1D	Elongation factor 1-delta	0.82
86	HYOU1	Hypoxia up-regulated protein 1	0.82
87	PKM	Pyruvate kinase PKM	0.82
88	RPL31	60S ribosomal protein L31	0.82
89	FAM98B	Protein FAM98B	0.82
90	KYAT3	Kynurenine--oxoglutarate transaminase 3	0.82
91	TARDBP	TAR DNA-binding protein 43	0.81
92	MYBBP1A	Myb-binding protein 1A	0.81
93	DPP3	Dipeptidyl peptidase 3	0.81
94	PKM	Pyruvate kinase	0.81
95	PYGL	Alpha-1,4 glucan phosphorylase	0.81
96	TRIM46	Tripartite motif-containing protein 46	0.81
97	YARS	Tyrosine--tRNA ligase, cytoplasmic	0.81
98	CSNK2A2	Casein kinase II subunit alpha'	0.81
99	HN1	Hematological and neurological-expressed 1 protein	0.81
100	SUPT16H	FACT complex subunit SPT16	0.81
101	CCDC124	Coiled-coil domain-containing protein 124	0.81
102	CLPP	ATP-dependent Clp protease proteolytic subunit	0.81
103	RPL12	60S ribosomal protein L12	0.81
104	FEN1	Flap endonuclease 1	0.81

105	HNRNPM	Heterogeneous nuclear ribonucleoprotein M	0.81
106	MYH10	Myosin-10	0.81
107	MAGOHB	Protein mago nashi homolog 2	0.81
108	EWSR1	RNA-binding protein EWS	0.81
109	OLA1	Obg-like ATPase 1	0.81
110	SND1	Staphylococcal nuclease domain-containing protein 1	0.81
111	CAP1	Adenylyl cyclase-associated protein 1	0.80
112	HIKESHI	Protein Hikeshi	0.80
113	PSMD4	26S proteasome non-ATPase regulatory subunit 4	0.80
114	TKT	Transketolase	0.80
115	CRKL	Crk-like protein	0.80
116	NAP1L4	Nucleosome assembly protein 1-like 4	0.80
117	SMARCA5	SWI/SNF-related matrix-associated actin-dependent regulator of chromatin subfamily	0.80
118	SF3B5	Splicing factor 3B subunit 5	0.80
119	NAP1L4	Nucleosome assembly protein 1-like 4	0.80
120	SF3B3	Splicing factor 3B subunit 3	0.80
121	EIF1	Eukaryotic translation initiation factor 1	0.80
122	ATP5B	ATP synthase subunit beta	0.80
123	CHMP5	Charged multivesicular body protein 5	0.80
124	SEPT2	Septin-2	0.80
125	DCTPP1	dCTP pyrophosphatase 1	0.80
126	TFG	Protein TFG	0.80
127	ALDOA	Fructose-bisphosphate aldolase A	0.80
128	FKBP10	Peptidylprolyl isomerase	0.80
129	LDHB	L-lactate dehydrogenase B chain	0.80
130	ATP5A1	ATP synthase subunit alpha, mitochondrial	0.80
131	SMARCE1	SWI/SNF-related matrix-associated actin-dependent regulator of chromatin subfamily	0.80
132	RPS27	40S ribosomal protein S27	0.80
133	SF3B1	Splicing factor 3B subunit 1	0.80
134	PPP4C	Serine/threonine-protein phosphatase	0.80
135	ACOT7	Cytosolic acyl coenzyme A thioester hydrolase	0.80
136	ATP5B	ATP synthase subunit beta, mitochondrial	0.80
137	MYBBP1A	Myb-binding protein 1A	0.80
138	SULT1B1	Sulfotransferase family cytosolic 1B member 1	0.80
139	COPE	Coatomer protein complex, subunit epsilon, isoform CRA_g	0.80
140	VIM	Vimentin	0.80
141	TMEM109	Transmembrane protein 109	0.79
142	ALDOA	Fructose-bisphosphate aldolase	0.79
143	PYGB	Glycogen phosphorylase, brain form	0.79
144	KRI1	Protein KRI1 homolog	0.79
145	EIF2S1	Eukaryotic translation initiation factor 2 subunit 1	0.79
146	HIST1H1E	Histone H1.4	0.79
147	CCT5	T-complex protein 1 subunit epsilon	0.79
148	FHL1	Four and a half LIM domains protein 1	0.79
149	MCM2	DNA helicase	0.79
150	ACP1	Low molecular weight phosphotyrosine protein phosphatase	0.79
151	TNPO3	Transportin-3	0.79
152	NME1-NME2	Nucleoside diphosphate kinase	0.79
153	NIFK	MKI67 FHA domain-interacting nucleolar phosphoprotein	0.79
154	FDPS	Farnesyl diphosphate synthase (Farnesyl pyrophosphate synthetase, dimethylallyltrans	0.79
155	MARS	Methionine--tRNA ligase, cytoplasmic	0.79
156	CLEC16A	Protein CLEC16A	0.79
157	HSD17B4	Peroxisomal multifunctional enzyme type 2	0.79
158	RAB1B	Ras-related protein Rab-1B	0.79
159	GMPS	GMP synthase [glutamine-hydrolyzing]	0.79

160	LDHA	L-lactate dehydrogenase A chain	0.79
161	HMGA1	High mobility group protein HMG-I/HMG-Y	0.79
162	COL6A2	Collagen alpha-2(VI) chain	0.79
163	RPL8	60S ribosomal protein L8	0.79
164	DBR1	Lariat debranching enzyme	0.79
165	CSDE1	Cold shock domain-containing protein E1	0.79
166	EIF3J	Eukaryotic translation initiation factor 3 subunit J	0.79
167	FKBP5	Peptidyl-prolyl cis-trans isomerase FKBP5	0.79
168	STIP1	Stress-induced-phosphoprotein 1	0.79
169	ACOT7	Cytosolic acyl coenzyme A thioester hydrolase	0.79
170	ST13	Hsc70-interacting protein	0.79
171	NIFK	MKI67 FHA domain-interacting nucleolar phosphoprotein	0.78
172	HEXIM1	Protein HEXIM1	0.78
173	VDAC1	Voltage-dependent anion-selective channel protein 1	0.78
174	NME1	Nucleoside diphosphate kinase A	0.78
175	RPL6	60S ribosomal protein L6	0.78
176	DDX39B	Spliceosome RNA helicase DDX39B	0.78
177	EBNA1BP2	EBNA1 binding protein 2, isoform CRA_d	0.78
178	CBX3	Chromobox protein homolog 3	0.78
179	UBB	Polyubiquitin-B	0.78
180	ESPNL	Espin-like protein	0.78
181	AHCY	Adenosylhomocysteinase	0.78
182	NAA50	N-alpha-acetyltransferase 50	0.78
183	HSPE1-MOB4	HSPE1-MOB4 readthrough	0.78
184	RPL18	60S ribosomal protein L18	0.78
185	AKAP12	A-kinase anchor protein 12	0.78
186	PA2G4	Proliferation-associated protein 2G4	0.78
187	RPS18	40S ribosomal protein S18	0.78
188	MSH6	DNA mismatch repair protein Msh6	0.78
189	PCBP2	Poly(rC)-binding protein 2	0.78
190	PEBP1	Phosphatidylethanolamine-binding protein 1	0.78
191	DISC1	Disrupted in schizophrenia 1 isoform 49	0.78
192	ATP5B	ATP synthase subunit beta, mitochondrial	0.78
193	QARS	Glutamine--tRNA ligase	0.78
194	KARS	Lysine--tRNA ligase	0.78
195	DDX23	Probable ATP-dependent RNA helicase DDX23	0.78
196	MRPL3	39S ribosomal protein L3, mitochondrial	0.78
197	PSMB6	Proteasome subunit beta type-6	0.78
198	CANX	Calnexin	0.78
199	LANCL1	LanC-like protein 1	0.78
200	RAB1A	Ras-related protein Rab-1A	0.77
201	EEF1D	Elongation factor 1-delta	0.77
202	FKBP1A	Peptidylprolyl isomerase	0.77
203	PFN2	Profilin	0.77
204	PRMT3	Protein arginine N-methyltransferase 3	0.77
205	VDAC1	Voltage-dependent anion-selective channel protein 1	0.77
206	RPL22	60S ribosomal protein L22	0.77
207	RAB2A	Ras-related protein Rab-2A	0.77
208	RPS20	40S ribosomal protein S20	0.77
209	PABPC4	Polyadenylate-binding protein 4	0.77
210	GCN1	eIF-2-alpha kinase activator GCN1	0.77
211	HNRNPU	Heterogeneous nuclear ribonucleoprotein U	0.77
212	ATOX1	Copper transport protein ATOX1	0.77
213	MYL6	Myosin light polypeptide 6	0.77
214	PMPCB	Mitochondrial-processing peptidase subunit beta	0.77

215	RARS	Arginine--tRNA ligase, cytoplasmic	0.77
216	PSAT1	Phosphoserine aminotransferase	0.77
217	IDH1	Isocitrate dehydrogenase [NADP] cytoplasmic	0.77
218	COX17	Cytochrome c oxidase copper chaperone	0.77
219	DDX5	Probable ATP-dependent RNA helicase DDX5	0.77
220	NUCKS1	Nuclear ubiquitous casein and cyclin-dependent kinase substrate 1	0.77
221	RPL23	60S ribosomal protein L23	0.77
222	RDX	Radixin	0.77
223	FAHD2A	Fumarylacetoacetate hydrolase domain-containing protein 2A	0.77
224	TXNDC9	Thioredoxin domain containing 9, isoform CRA_a	0.77
225	SRM	Spermidine synthase	0.77
226	MDH1	Malate dehydrogenase, cytoplasmic	0.77
227	PRPF4	U4/U6 small nuclear ribonucleoprotein Prp4	0.77
228	PRDX2	Peroxiredoxin-2	0.77
229	HIST1H4A	Histone H4	0.77
230	PRDX1	Peroxiredoxin-1	0.76
231	DCTN2	Dynactin subunit 2	0.76
232	LAP3	Cytosol aminopeptidase	0.76
233	HARS2	Probable histidine--tRNA ligase, mitochondrial	0.76
234	ILF2	Interleukin enhancer-binding factor 2	0.76
235	UBE2O	(E3-independent) E2 ubiquitin-conjugating enzyme	0.76
236	AK2	Adenylate kinase 2, mitochondrial	0.76
237	ADSS	Adenylosuccinate synthetase isozyme 2	0.76
238	AIMP1	Aminoacyl tRNA synthase complex-interacting multifunctional protein 1	0.76
239	PRDX1	Peroxiredoxin-1	0.76
240	DFFA	DNA fragmentation factor subunit alpha	0.76
241	RPL8	60S ribosomal protein L8	0.76
242	NFYA	Nuclear transcription factor Y subunit alpha	0.76
243	RPL35	60S ribosomal protein L35	0.76
244	MAK16	Protein MAK16 homolog	0.76
245	ALDH16A1	Aldehyde dehydrogenase family 16 member A1	0.76
246	CAMSAP2	Calmodulin-regulated spectrin-associated protein 2	0.76
247	DCTN1	Dynactin subunit 1	0.76
248	CCT6A	T-complex protein 1 subunit zeta	0.76
249	TST	Thiosulfate sulfurtransferase	0.76
250	RRP12	RRP12-like protein	0.76
251	TUFM	Elongation factor Tu, mitochondrial	0.76
252	FLNB	Filamin-B	0.76
253	LASP1	LIM and SH3 domain protein 1	0.76
254	MATR3	Matrin-3	0.76
255	DCTN2	Dynactin subunit 2	0.76
256	TPM1	Tropomyosin 1 (Alpha), isoform CRA_f	0.76
257	RPS3	40S ribosomal protein S3	0.76
258	IARS	Isoleucine--tRNA ligase, cytoplasmic	0.75
259	ACYP1	Acylphosphatase	0.75
260	ELOB	Elongin-B	0.75
261	SMC1A	Structural maintenance of chromosomes protein 1A	0.75
262	EIF3D	Eukaryotic translation initiation factor 3 subunit D	0.75
263	DNMT1	DNA (cytosine-5)-methyltransferase 1	0.75
264	EXOSC2	Exosome complex component RRP4	0.75
265	PRDX2	Peroxiredoxin 2, isoform CRA_a	0.75
266	VPS35	Vacuolar protein sorting-associated protein 35	0.75
267	CSE1L	Exportin-2	0.75
268	MCM7	DNA replication licensing factor MCM7	0.75
269	HNRNPU	Heterogeneous nuclear ribonucleoprotein U	0.75

270	SRP14	Signal recognition particle 14 kDa protein	0.75
271	TUBA1B	Tubulin alpha-1B chain	0.75
272	SLIRP	SRA stem-loop-interacting RNA-binding protein, mitochondrial	0.75
273	SARS	Serine--tRNA ligase, cytoplasmic	0.75
274	RECQL5	ATP-dependent DNA helicase Q5	0.75
275	CDV3	Protein CDV3 homolog	0.75
276	WDR77	Methylosome protein 50	0.75
277	SRPRB	Signal recognition particle receptor subunit beta	0.75
278	EIF3G	Eukaryotic translation initiation factor 3 subunit G	0.75
279	TRAP1	Heat shock protein 75 kDa, mitochondrial	0.75
280	RPS27L	40S ribosomal protein S27	0.75
281	EIF4A3	Eukaryotic initiation factor 4A-III	0.75
282	MCM4	DNA replication licensing factor MCM4	0.75
283	RPL17	60S ribosomal protein L17	0.75
284	SF3B2	Splicing factor 3B subunit 2	0.75
285	CHMP4B	Charged multivesicular body protein 4b	0.75
286	MMP3	Stromelysin-1	0.75
287	CCT8	T-complex protein 1 subunit theta	0.75
288	PPP2R1A	Serine/threonine-protein phosphatase 2A 65 kDa regulatory subunit A alpha isoform	0.75
289	TBR1	T-box brain protein 1	0.75
290	ASNS	Asparagine synthetase [glutamine-hydrolyzing]	0.75
291	UCHL5	Ubiquitin carboxyl-terminal hydrolase	0.75
292	CYB5A	Cytochrome b5	0.75
293	SKP1	S-phase kinase-associated protein 1	0.75
294	BAG3	BAG family molecular chaperone regulator 3	0.75
295	CELF1	CUG triplet repeat, RNA binding protein 1, isoform CRA_c	0.75
296	PAF1	RNA polymerase II-associated factor 1 homolog	0.74
297	DPYSL5	Dihydropyrimidinase-related protein 5	0.74
298	CCT4	T-complex protein 1 subunit delta	0.74
299	ADSL	Adenylosuccinate lyase	0.74
300	API5	Apoptosis inhibitor 5	0.74
301	AIMP1	Aminoacyl tRNA synthase complex-interacting multifunctional protein 1	0.74
302	LARP1	La-related protein 1	0.74
303	MRPS26	28S ribosomal protein S26, mitochondrial	0.74
304	PSMD12	26S proteasome non-ATPase regulatory subunit 12	0.74
305	BOP1	Ribosome biogenesis protein BOP1	0.74
306	RAB14	Ras-related protein Rab-14	0.74
307	FUBP1	Far upstream element-binding protein 1	0.74
308	DDB1	DNA damage-binding protein 1	0.74
309	AP3B1	AP-3 complex subunit beta-1	0.74
310	PSMA4	Proteasome endopeptidase complex	0.74
311	AP1B1	AP-1 complex subunit beta-1	0.74
312	EXOSC2	Exosome complex component RRP4	0.74
313	ADAR	Double-stranded RNA-specific adenosine deaminase	0.74
314	MAPRE1	Microtubule-associated protein RP/EB family member 1	0.74
315	EIF3M	Eukaryotic translation initiation factor 3 subunit M	0.74
316	PRPF4B	Serine/threonine-protein kinase PRP4 homolog	0.74
317	PSMC5	26S protease regulatory subunit 8	0.74
318	RPS16	40S ribosomal protein S16	0.74
319	THUMPD1	THUMP domain-containing protein 1	0.74
320	ZRANB2	Zinc finger Ran-binding domain-containing protein 2	0.74
321	SNX2	Sorting nexin-2	0.74
322	ETF1	Eukaryotic peptide chain release factor subunit 1	0.74
323	EIF3M	Eukaryotic translation initiation factor 3 subunit M	0.74
324	BUB3	Mitotic checkpoint protein BUB3	0.74

325	RPL32	60S ribosomal protein L32	0.74
326	EPN1	Epsin-1	0.73
327	EZR	Ezrin	0.73
328	CORO1B	Coronin-1B	0.73
329	MOGS	Mannosyl-oligosaccharide glucosidase	0.73
330	ADSL	Adenylosuccinate lyase	0.73
331	IMPDH1	Inosine-5'-monophosphate dehydrogenase 1	0.73
332	PDCD6IP	Programmed cell death 6-interacting protein	0.73
333	STUB1	E3 ubiquitin-protein ligase CHIP	0.73
334	PMPCA	Mitochondrial-processing peptidase subunit alpha	0.73
335	DCXR	L-xylulose reductase	0.73
336	ANKRD17	Ankyrin repeat domain-containing protein 17	0.73
337	VARS	Valine--tRNA ligase	0.73
338	PCCA	Propionyl-CoA carboxylase alpha chain, mitochondrial	0.73
339	DUT	Deoxyuridine 5'-triphosphate nucleotidohydrolase, mitochondrial	0.73
340	EEF2	Elongation factor 2	0.73
341	PDLIM1	PDZ and LIM domain protein 1	0.73
342	CTNNBL1	Beta-catenin-like protein 1	0.73
343	VBP1	Prefoldin subunit 3	0.73
344	PSMB2	Proteasome subunit beta type-2	0.73
345	MTHFD1L	Monofunctional C1-tetrahydrofolate synthase, mitochondrial	0.73
346	EPS15L1	Epidermal growth factor receptor substrate 15-like 1	0.73
347	SRCAP	Helicase SRCAP	0.73
348	RTN4	Reticulon	0.73
349	SMC1A	Structural maintenance of chromosomes protein	0.73
350	CHORDC1	Cysteine and histidine-rich domain-containing protein 1	0.73
351	ARL6IP4	ADP-ribosylation factor-like protein 6-interacting protein 4	0.73
352	RCN2	Reticulocalbin-2	0.73
353	EWSR1	RNA-binding protein EWS	0.73
354	UBTF	Nucleolar transcription factor 1	0.73
355	TRIM28	Transcription intermediary factor 1-beta	0.73
356	CTPS1	CTP synthase 1	0.73
357	VDAC2	Voltage-dependent anion-selective channel protein 2	0.73
358	PTMS	Parathymosin	0.73
359	GNPDA1	Glucosamine-6-phosphate isomerase	0.73
360	PLIN3	Perilipin-3	0.73
361	STAG2	Cohesin subunit SA-2	0.73
362	GEMIN5	Gem-associated protein 5	0.73
363	KTN1	Kinectin	0.73
364	TXNL1	Thioredoxin-like protein 1	0.73
365	SNRNP70	U1 small nuclear ribonucleoprotein 70 kDa	0.73
366	HIST1H2BN	Histone H2B	0.73
367	BOP1	Ribosome biogenesis protein BOP1	0.73
368	SSRP1	FACT complex subunit SSRP1	0.73
369	PPP1R7	Protein phosphatase 1 regulatory subunit 7	0.73
370	NAMPT	Nicotinamide phosphoribosyltransferase	0.73
371	RABGAP1	Rab GTPase-activating protein 1	0.72
372	RBM17	Splicing factor 45	0.72
373	PRPSAP2	Phosphoribosyl pyrophosphate synthase-associated protein 2	0.72
374	GAFA3	FGF-2 activity-associated protein 3	0.72
375	S100A13	Protein S100-A13	0.72
376	EIF3I	Eukaryotic translation initiation factor 3 subunit I	0.72
377	EIF2S1	Eukaryotic translation initiation factor 2 subunit 1	0.72
378	AGL	Glycogen debranching enzyme	0.72
379	FLNA	Filamin-A	0.72

380	SPG20	Spartin	0.72
381	EEF1A1	Elongation factor 1-alpha 1	0.72
382	PPID	Peptidyl-prolyl cis-trans isomerase D	0.72
383	CNDP2	Cytosolic non-specific dipeptidase	0.72
384	ELAC2	ElaC homolog 2 (E. coli), isoform CRA_a	0.72
385	RPS10-NUDT3	RPS10-NUDT3 readthrough	0.72
386	STARD9	StAR-related lipid transfer protein 9	0.72
387	GINS3	DNA replication complex GINS protein PSF3	0.72
388	NAP1L1	Nucleosome assembly protein 1-like 1	0.72
389	OTUB1	Ubiquitin thioesterase	0.72
390	ACACA	Acetyl-CoA carboxylase 1	0.72
391	CHD4	Chromodomain-helicase-DNA-binding protein 4	0.72
392	ALPI	Intestinal-type alkaline phosphatase	0.72
393	PDCD5	Programmed cell death protein 5	0.72
394	AHCYL2	Adenosylhomocysteinase	0.72
395	PSMA6	Proteasome subunit alpha type	0.72
396	KIN	DNA/RNA-binding protein KIN17	0.72
397	BCLAF1	Bcl-2-associated transcription factor 1	0.72
398	VPS26A	Vacuolar protein sorting-associated protein 26A	0.72
399	AP2B1	AP-2 complex subunit beta	0.72
400	CDK1	Cyclin-dependent kinase 1	0.72
401	PPIE	Peptidyl-prolyl cis-trans isomerase	0.72
402	TPM3	Tropomyosin alpha-3 chain	0.72
403	COX6B1	Cytochrome c oxidase subunit 6B1	0.71
404	MYL12A	Myosin regulatory light chain 12A	0.71
405	EIF3CL	Eukaryotic translation initiation factor 3 subunit C-like protein	0.71
406	HIST1H2BJ	Histone H2B type 1-J	0.71
407	MDN1	Midasin	0.71
408	DDX18	ATP-dependent RNA helicase DDX18	0.71
409	BAZ1B	Tyrosine-protein kinase BAZ1B	0.71
410	COPA	Coatomer subunit alpha	0.71
411	ARHGDIA	Rho GDP-dissociation inhibitor 1	0.71
412	GART	Trifunctional purine biosynthetic protein adenosine-3	0.71
413	DYNC1H1	Cytoplasmic dynein 1 heavy chain 1	0.71
414	GTF2F1	General transcription factor IIF subunit 1	0.71
415	ZBTB1	Zinc finger and BTB domain-containing protein 1	0.71
416	RPS29	40S ribosomal protein S29	0.71
417	PAPOLA	Poly(A) polymerase alpha	0.71
418	PRKAR2A	cAMP-dependent protein kinase type II-alpha regulatory subunit	0.71
419	EXOSC5	Exosome complex component RRP46	0.71
420	LRRFIP1	Leucine-rich repeat flightless-interacting protein 1	0.71
421	CARS	Cysteine--tRNA ligase, cytoplasmic	0.71
422	PSME1	Proteasome activator complex subunit 1	0.71
423	KPNA1	Importin subunit alpha-5	0.71
424	CCDC43	CCDC43 protein	0.71
425	PPT1	Palmitoyl-protein thioesterase 1	0.71
426	CDV3	Protein CDV3 homolog	0.71
427	MRPS22	28S ribosomal protein S22, mitochondrial	0.71
428	PPA2	Inorganic pyrophosphatase 2, mitochondrial	0.71
429	KCTD5	BTB/POZ domain-containing protein KCTD5	0.71
430	EIF4G1	Eukaryotic translation initiation factor 4 gamma 1	0.71
431	C7orf50	Uncharacterized protein C7orf50	0.71
432	GNPDA2	Glucosamine-6-phosphate isomerase 2	0.71
433	SNRNP200	U5 small nuclear ribonucleoprotein 200 kDa helicase	0.71
434	RBM17	Splicing factor 45	0.71

435	RACK1	Receptor of-activated protein C kinase 1	0.71
436	TBCB	Tubulin-folding cofactor B	0.71
437	CDKN2A	Cyclin-dependent kinase inhibitor 2A	0.71
438	TXNDC5	Thioredoxin domain-containing protein 5	0.71
439	ATXN10	Ataxin-10	0.71
440	C19orf25	UPF0449 protein C19orf25	0.71
441	DIABLO	Diablo homolog, mitochondrial	0.71
442	P4HA1	Prolyl 4-hydroxylase subunit alpha-1	0.71
443	VARS	Valine--tRNA ligase	0.71
444	EFTUD2	116 kDa U5 small nuclear ribonucleoprotein component	0.71
445	PPP2R5D	Serine/threonine-protein phosphatase 2A 56 kDa regulatory subunit delta isoform	0.71
446	FASTKD2	FAST kinase domain-containing protein 2, mitochondrial	0.71
447	CIAO1	Probable cytosolic iron-sulfur protein assembly protein CIAO1	0.71
448	COPS8	COP9 signalosome complex subunit 8	0.70
449	SETD3	Histone-lysine N-methyltransferase setd3	0.70
450	YTHDF1	YTH domain-containing family protein 1	0.70
451	ME2	NAD-dependent malic enzyme, mitochondrial	0.70
452	ACTR2	Actin-related protein 2	0.70
453	PSMD3	26S proteasome non-ATPase regulatory subunit 3	0.70
454	SYAP1	Synapse-associated protein 1	0.70
455	UPF1	Regulator of nonsense transcripts 1	0.70
456	TUBA4A	Tubulin alpha-4A chain	0.70
457	STAM2	Signal transducing adapter molecule 2	0.70
458	CUL4B	Cullin 4B, isoform CRA_e	0.70
459	EML5	Echinoderm microtubule-associated protein-like 5	0.70
460	ARPC4-TTLL3	ARPC4-TTLL3 readthrough	0.70
461	CYB5R3	NADH-cytochrome b5 reductase 3	0.70
462	LARP4	La-related protein 4	0.70
463	PRPF19	Pre-mRNA-processing factor 19	0.70
464	SH3KBP1	SH3 domain-containing kinase-binding protein 1	0.70
465	POLR2E	DNA-directed RNA polymerases I, II, and III subunit RPABC1	0.70
466	KPNA5	Importin subunit alpha-6	0.70
467	CAD	CAD protein	0.70
468	KCTD12	BTB/POZ domain-containing protein KCTD12	0.70
469	PHLPP1	PH domain leucine-rich repeat-containing protein phosphatase 1	0.70
470	RPS28	40S ribosomal protein S28	0.70
471	CLUH	Clustered mitochondria protein homolog	0.70
472	RANGAP1	Ran GTPase-activating protein 1	0.70
473	SF3A2	Splicing factor 3A subunit 2	0.70
474	TOMM34	Mitochondrial import receptor subunit TOM34	0.70
475	UBE2V1	Ubiquitin-conjugating enzyme E2 variant 1	0.70
476	EIF3C	Eukaryotic translation initiation factor 3 subunit C	0.70
477	ABCF2	ATP-binding cassette sub-family F member 2	0.70
478	EMD	Emerin	0.70
479	TACO1	Translational activator of cytochrome c oxidase 1	0.70
480	VTA1	Chromosome 6 open reading frame 55, isoform CRA_b	0.70
481	NOP14	Nucleolar protein 14	0.70
482	DDI2	Protein DDI1 homolog 2	0.70
483	KDELC2	KDEL motif-containing protein 2	0.70
484	POLR2G	DNA-directed RNA polymerase II subunit RPB7	0.70
485	RPL18	60S ribosomal protein L18	0.69
486	PHB	Prohibitin	0.69
487	HK2	Hexokinase-2	0.69
488	IPO8	Importin-8	0.69
489	NDUFS3	NADH dehydrogenase [ubiquinone] iron-sulfur protein 3, mitochondrial	0.69

490	KPNA1	Importin subunit alpha-5	0.69
491	GINS4	DNA replication complex GINS protein SLD5	0.69
492	ATP1A1	Sodium/potassium-transporting ATPase subunit alpha-1	0.69
493	RWDD1	RWD domain-containing protein 1	0.69
494	UBE2O	(E3-independent) E2 ubiquitin-conjugating enzyme	0.69
495	AIMP2	Aminoacyl tRNA synthase complex-interacting multifunctional protein 2	0.69
496	SNX5	Sorting nexin-5	0.69
497	DDX3X	ATP-dependent RNA helicase DDX3X	0.69
498	SRSF5	Serine/arginine-rich splicing factor 5	0.69
499	FSCN1	Fascin	0.69
500	CALD1	Caldesmon	0.69
501	RAC3	Ras-related C3 botulinum toxin substrate 3	0.69
502	RAB14	Ras-related protein Rab-14	0.69
503	SRSF4	Serine/arginine-rich-splicing factor 4	0.69
504	FAM50B	Protein FAM50B	0.69
505	TKFC	Triokinase/FMN cyclase	0.69
506	NAP1L1	Nucleosome assembly protein 1-like 1	0.69
507	HMGB2	High mobility group protein B2	0.69
508	PRPF4B	Serine/threonine-protein kinase PRP4 homolog	0.69
509	MYL12A	Myosin regulatory light chain 12A	0.69
510	PPAT	Amidophosphoribosyltransferase	0.69
511	RPS25	40S ribosomal protein S25	0.69
512	FKBP10	Peptidyl-prolyl cis-trans isomerase FKBP10	0.69
513	HSPA14	Heat shock 70 kDa protein 14	0.69
514	PLCB3	1-phosphatidylinositol 4,5-bisphosphate phosphodiesterase beta-3	0.69
515	POLR2L	DNA-directed RNA polymerases I, II, and III subunit RPABC5	0.69
516	MRPL47	39S ribosomal protein L47, mitochondrial	0.69
517	IMPDH1	Inosine-5'-monophosphate dehydrogenase 1	0.69
518	ARPC5	Actin-related protein 2/3 complex subunit 5	0.69
519	PAFAH1B1	Platelet-activating factor acetylhydrolase IB subunit alpha	0.69
520	DDX10	RNA helicase	0.69
521	RPL11	60S ribosomal protein L11	0.68
522	CFAP20	Cilia- and flagella-associated protein 20	0.68
523	AKR1A1	Alcohol dehydrogenase [NADP(+)]	0.68
524	CCAR2	Cell cycle and apoptosis regulator protein 2	0.68
525	HSPB11	Chromosome 1 open reading frame 41, isoform CRA_b	0.68
526	POP1	Ribonucleases P/MRP protein subunit POP1	0.68
527	MIB2	E3 ubiquitin-protein ligase MIB2	0.68
528	RAD23B	UV excision repair protein RAD23 homolog B	0.68
529	PDE12	2',5'-phosphodiesterase 12	0.68
530	NACA	Nascent polypeptide-associated complex subunit alpha	0.68
531	PSME2	Proteasome activator complex subunit 2	0.68
532	TRMT1L	TRMT1-like protein	0.68
533	ATP5O	ATP synthase subunit O, mitochondrial	0.68
534	RPS11	40S ribosomal protein S11	0.68
535	DHRS7	Dehydrogenase/reductase SDR family member 7	0.68
536	SLK	STE20-like serine/threonine-protein kinase	0.68
537	PNN	Pinin	0.68
538	MYLK	Myosin light chain kinase, smooth muscle	0.68
539	SUPT6H	Transcription elongation factor SPT6	0.68
540	TPM3	Tropomyosin alpha-3 chain	0.68
541	UBASH3B	Ubiquitin-associated and SH3 domain-containing protein B	0.68
542	SRI	Sorcin	0.68
543	COPS7B	COP9 signalosome complex subunit 7b	0.68
544	CALCOCO2	Calcium-binding and coiled-coil domain-containing protein 2	0.68

545	GORASP2	Golgi reassembly-stacking protein 2	0.68
546	CUTA	Protein CutA	0.68
547	TBL3	Transducin beta-like protein 3	0.68
548	RPRD1B	Regulation of nuclear pre-mRNA domain-containing protein 1B	0.68
549	SRPK1	SRSF protein kinase 1	0.68
550	ZNF428	Zinc finger protein 428	0.68
551	PSMA3	Proteasome subunit alpha type-3	0.68
552	VPS29	Vacuolar protein sorting-associated protein 29	0.68
553	TUBB3	Tubulin beta-3 chain	0.68
554	NOP16	Nucleolar protein 16	0.68
555	PSMB1	Proteasome subunit beta type-1	0.68
556	HECTD1	E3 ubiquitin-protein ligase HECTD1	0.68
557	FLYWCH1	FLYWCH-type zinc finger-containing protein 1	0.67
558	TAX1BP3	Tax1-binding protein 3	0.67
559	BAG2	BAG family molecular chaperone regulator 2	0.67
560	RAB10	Ras-related protein Rab-10	0.67
561	RAB8A	Ras-related protein Rab-8A	0.67
562	CISD1	CDGSH iron-sulfur domain-containing protein 1	0.67
563	AP1S1	AP-1 complex subunit sigma-1A	0.67
564	EIF2A	Eukaryotic translation initiation factor 2A	0.67
565	RPL13	60S ribosomal protein L13	0.67
566	GABARAPL2	Gamma-aminobutyric acid receptor-associated protein-like 2	0.67
567	IDH3B	Isocitrate dehydrogenase [NAD] subunit, mitochondrial	0.67
568	KCTD9	BTB/POZ domain-containing protein KCTD9	0.67
569	RAC2	Ras-related C3 botulinum toxin substrate 2	0.67
570	HNRNPM	Heterogeneous nuclear ribonucleoprotein M	0.67
571	MFAP1	Microfibrillar-associated protein 1	0.67
572	FBL	rRNA 2'-O-methyltransferase fibrillarin	0.67
573	RPS8	40S ribosomal protein S8	0.67
574	CTSB	Cathepsin B	0.67
575	HMGCS1	Hydroxymethylglutaryl-CoA synthase, cytoplasmic	0.67
576	ACTL6A	Actin-like protein 6A	0.67
577	CPXM2	Inactive carboxypeptidase-like protein X2	0.67
578	TCEA1	Transcription elongation factor A protein 1	0.67
579	NCAPD2	Condensin complex subunit 1	0.67
580	UNC45A	Protein unc-45 homolog A	0.67
581	RPL11	60S ribosomal protein L11	0.67
582	EEF1A2	Elongation factor 1-alpha 2	0.67
583	AASDHPPT	L-aminoadipate-semialdehyde dehydrogenase-phosphopantetheinyl transferase	0.67
584	PGAM5	Serine/threonine-protein phosphatase PGAM5, mitochondrial	0.67
585	TUBB	Tubulin beta chain	0.67
586	SON	Protein SON	0.67
587	C17orf49	Chromosome 17 open reading frame 49	0.66
588	EHD4	EH domain-containing protein 4	0.66
589	COPB2	Coatomer subunit beta'	0.66
590	PFDN6	Prefoldin subunit 6	0.66
591	RBM10	RNA binding motif protein 10, isoform CRA_d	0.66
592	ATP6V1B2	V-type proton ATPase subunit B, brain isoform	0.66
593	CUL1	Cullin-1	0.66
594	CHMP4A	Charged multivesicular body protein 4a	0.66
595	ATP6V1A	V-type proton ATPase catalytic subunit A	0.66
596	GLRX3	Glutaredoxin-3	0.66
597	EIF3E	Eukaryotic translation initiation factor 3 subunit E	0.66
598	TUBB2A	Tubulin beta-2A chain	0.66
599	HMGB2	High mobility group protein B2	0.66

600	ATP6V1B2	V-type proton ATPase subunit B, brain isoform	0.66
601	DYNC1I1	Cytoplasmic dynein 1 intermediate chain 1	0.66
602	EDC4	Enhancer of mRNA-decapping protein 4	0.66
603	LGALSL	Galectin-related protein	0.66
604	ANXA1	Annexin	0.66
605	USP5	Ubiquitin carboxyl-terminal hydrolase 5	0.66
606	FAHD2B	Fumarylacetoacetate hydrolase domain-containing protein 2B	0.66
607	RRM2	Ribonucleoside-diphosphate reductase subunit M2	0.66
608	DCUN1D5	DCN1-like protein 5	0.66
609	MAP1LC3B	Microtubule-associated protein 1 light chain 3 beta, isoform CRA_f	0.66
610	DPYSL5	Dihydropyrimidinase-related protein 5	0.66
611	ATP1B3	Sodium/potassium-transporting ATPase subunit beta-3	0.66
612	MARCKSL1	MARCKS-related protein	0.66
613	ZFR	Zinc finger RNA-binding protein	0.66
614	RUFY1	RUN and FYVE domain-containing protein 1	0.66
615	IPO4	Importin-4	0.66
616	DNPH1	2'-deoxynucleoside 5'-phosphate N-hydrolase 1	0.66
617	XPO5	Exportin-5	0.66
618	DNAJA2	DnaJ homolog subfamily A member 2	0.66
619	CUL5	Cullin-5	0.66
620	NAA10	N-alpha-acetyltransferase 10	0.66
621	GTF3C5	General transcription factor 3C polypeptide 5	0.66
622	TRMT112	Multifunctional methyltransferase subunit TRM112-like protein	0.66
623	RBMX	RNA-binding motif protein, X chromosome	0.66
624	TECR	Very-long-chain enoyl-CoA reductase	0.66
625	COPB1	Coatomer subunit beta	0.66
626	PSME3	Proteasome activator complex subunit 3	0.66
627	HMOX1	Heme oxygenase 1	0.66
628	DPYSL2	Dihydropyrimidinase-related protein 2	0.65
629	GTF2IRD2B	General transcription factor II-I repeat domain-containing protein 2B	0.65
630	KPNA2	Importin subunit alpha-1	0.65
631	MARCKS	Myristoylated alanine-rich C-kinase substrate	0.65
632	LAMTOR1	Ragulator complex protein LAMTOR1	0.65
633	DNAJC7	DnaJ homolog subfamily C member 7	0.65
634	AQP3	Aquaporin-3	0.65
635	ALDH1A2	Retinal dehydrogenase 2	0.65
636	HM13	Minor histocompatibility antigen H13	0.65
637	MBD3	Methyl-CpG-binding domain protein 3	0.65
638	YWHAH	14-3-3 protein eta	0.65
639	ARF3	ADP-ribosylation factor 3	0.65
640	PDS5A	Sister chromatid cohesion protein PDS5 homolog A	0.65
641	HDAC1	Histone deacetylase 1	0.65
642	SRP9	Signal recognition particle 9 kDa protein	0.65
643	ATP5H	ATP synthase subunit d, mitochondrial	0.65
644	RPL13A	60S ribosomal protein L13a	0.65
645	PAPOLA	Poly(A) polymerase alpha	0.65
646	ARPC2	Actin-related protein 2/3 complex subunit 2	0.65
647	BCLAF1	Bcl-2-associated transcription factor 1	0.65
648	AARS2	Alanine--tRNA ligase, mitochondrial	0.65
649	RAB7A	Ras-related protein Rab-7a	0.65
650	NAA10	N-alpha-acetyltransferase 10	0.65
651	DARS	Aspartate--tRNA ligase, cytoplasmic	0.65
652	TDP1	Tyrosyl-DNA phosphodiesterase 1	0.65
653	DYNC1LI1	Cytoplasmic dynein 1 light intermediate chain 1	0.65
654	ARIH1	E3 ubiquitin-protein ligase ARIH1	0.65

655	CLUH	Clustered mitochondria protein homolog	0.64
656	ACP1	Acid phosphatase 1, soluble, isoform CRA_d	0.64
657	SNX6	Sorting nexin	0.64
658	RPL36	60S ribosomal protein L36	0.64
659	UBAP2L	Ubiquitin-associated protein 2-like	0.64
660	ARF5	ADP-ribosylation factor 5	0.64
661	CDK5RAP3	CDK5 regulatory subunit-associated protein 3	0.64
662	PITPNB	Phosphatidylinositol transfer protein beta isoform	0.64
663	VAMP7	Vesicle-associated membrane protein 7	0.64
664	PRPF40A	Pre-mRNA-processing factor 40 homolog A	0.64
665	PUS7	Pseudouridylate synthase 7 homolog	0.64
666	CAP1	Adenylyl cyclase-associated protein 1	0.64
667	UBXN1	UBX domain-containing protein 1	0.64
668	SHTN1	Shootin-1	0.64
669	HDLBP	High density lipoprotein binding protein (Vigilin), isoform CRA_a	0.64
670	CDC73	Parafibromin	0.64
671	RPL38	60S ribosomal protein L38	0.64
672	DDA1	DET1- and DDB1-associated protein 1	0.64
673	THOC6	THO complex subunit 6 homolog	0.64
674	URM1	Ubiquitin-related modifier 1	0.64
675	QKI	Protein quaking	0.64
676	NPIPA2	Nuclear pore complex-interacting protein family member A2	0.64
677	TMOD3	Tropomodulin-3	0.64
678	LRRC47	Leucine-rich repeat-containing protein 47	0.64
679	OGDH	2-oxoglutarate dehydrogenase, mitochondrial	0.64
680	ALDH1B1	Aldehyde dehydrogenase X, mitochondrial	0.64
681	CCAR2	Cell cycle and apoptosis regulator protein 2	0.63
682	UGDH	UDP-glucose 6-dehydrogenase	0.63
683	PPA2	Inorganic pyrophosphatase 2, mitochondrial	0.63
684	SBDS	Ribosome maturation protein SBDS	0.63
685	DARS	Aspartate--tRNA ligase, cytoplasmic	0.63
686	HBS1L	HBS1-like protein	0.63
687	STAU1	Double-stranded RNA-binding protein Staufen homolog 1	0.63
688	ALDH7A1	Alpha-aminoadipic semialdehyde dehydrogenase	0.63
689	CDK2	Cyclin-dependent kinase 2	0.63
690	KIF11	Kinesin-like protein KIF11	0.63
691	WDR82	WD repeat-containing protein 82	0.63
692	MAT2B	Methionine adenosyltransferase 2 subunit beta	0.63
693	SART3	Squamous cell carcinoma antigen recognized by T-cells 3	0.63
694	MRI1	Methylthioribose-1-phosphate isomerase	0.63
695	RPL7	60S ribosomal protein L7	0.63
696	AKAP1	A-kinase anchor protein 1, mitochondrial	0.63
697	CNOT1	CCR4-NOT transcription complex subunit 1	0.63
698	TWF1	Twinfilin-1	0.63
699	PPP3CA	Serine/threonine-protein phosphatase	0.63
700	DAD1	Dolichyl-diphosphooligosaccharide--protein glycosyltransferase subunit DAD1	0.63
701	GPKOW	G patch domain and KOW motifs-containing protein	0.63
702	SCP2	Non-specific lipid-transfer protein	0.63
703	CUL3	Cullin-3	0.63
704	PACSIN2	Protein kinase C and casein kinase substrate in neurons protein 2	0.63
705	COPS4	COP9 signalosome complex subunit 4	0.63
706	C15orf57	Uncharacterized protein C15orf57	0.63
707	PSMD8	26S proteasome non-ATPase regulatory subunit 8	0.63
708	SCLY	Selenocysteine lyase	0.63
709	PAFAH1B3	Platelet-activating factor acetylhydrolase IB subunit gamma	0.63

710	PSMD7	26S proteasome non-ATPase regulatory subunit 7	0.62
711	PPIH	Peptidyl-prolyl cis-trans isomerase	0.62
712	MYO15A	Unconventional myosin-XV	0.62
713	VAMP2	Vesicle-associated membrane protein 2	0.62
714	AP2A1	AP-2 complex subunit alpha-1	0.62
715	H2AFY2	Histone H2A	0.62
716	ESYT1	Extended synaptotagmin-1	0.62
717	PHPT1	14 kDa phosphohistidine phosphatase	0.62
718	DRG2	Developmentally-regulated GTP-binding protein 2	0.62
719	NRDC	Nardilysin	0.62
720	AGBL4	Cytosolic carboxypeptidase 6	0.62
721	PES1	Pescadillo homolog	0.62
722	HAGH	Hydroxyacylglutathione hydrolase, mitochondrial	0.62
723	PFDN2	Prefoldin subunit 2	0.62
724	RAB6B	Ras-related protein Rab-6B	0.62
725	POLR2A	DNA-directed RNA polymerase subunit	0.62
726	COPG1	Coatomer subunit gamma-1	0.62
727	MTA1	Metastasis-associated protein MTA1	0.62
728	ZNF513	Zinc finger protein 513	0.62
729	MAT2B	Methionine adenosyltransferase 2 subunit beta	0.62
730	RPP30	Ribonuclease P protein subunit p30	0.62
731	UFC1	Ubiquitin-fold modifier-conjugating enzyme 1	0.62
732	SMARCE1	SWI/SNF-related matrix-associated actin-dependent regulator of chromatin subfamily	0.62
733	CDK4	Cyclin-dependent kinase 4	0.62
734	POLR1C	DNA-directed RNA polymerases I and III subunit RPAC1	0.62
735	PGM2	Phosphoglucomutase-2	0.61
736	HDAC2	Histone deacetylase 2	0.61
737	TPM2	Tropomyosin beta chain	0.61
738	SEC24C	Protein transport protein Sec24C	0.61
739	DCAF7	DDB1- and CUL4-associated factor 7	0.61
740	HUWE1	E3 ubiquitin-protein ligase HUWE1	0.61
741	UBE2T	Ubiquitin-conjugating enzyme E2 T	0.61
742	PRRC2C	Protein PRRC2C	0.61
743	ZNF345	Zinc finger protein 345	0.61
744	MRPS28	28S ribosomal protein S28, mitochondrial	0.61
745	DARS2	Aspartate--tRNA ligase, mitochondrial	0.61
746	TOP2A	DNA topoisomerase 2-alpha	0.61
747	EPB41L3	Band 4.1-like protein 3	0.61
748	EIF3K	Eukaryotic translation initiation factor 3 subunit K	0.61
749	BSG	Basigin	0.61
750	SRP54	Signal recognition particle 54 kDa protein	0.61
751	UBA52	Ubiquitin-60S ribosomal protein L40	0.61
752	NUP205	Nuclear pore complex protein Nup205	0.61
753	MYD88	Myeloid differentiation primary response protein MyD88	0.61
754	ENAH	Protein enabled homolog	0.61
755	COX7A2	Cytochrome c oxidase subunit 7A2, mitochondrial	0.61
756	CALM2	Calmodulin	0.61
757	UQCRC2	Cytochrome b-c1 complex subunit 2, mitochondrial	0.61
758	MRPS23	28S ribosomal protein S23, mitochondrial	0.61
759	IQGAP1	Ras GTPase-activating-like protein IQGAP1	0.61
760	SDF4	45 kDa calcium-binding protein	0.61
761	KPNA4	Importin subunit alpha-3	0.61
762	TRA2B	Transformer-2 protein homolog beta	0.61
763	CPOX	Oxygen-dependent coproporphyrinogen-III oxidase, mitochondrial	0.61
764	PMM2	Phosphomannomutase	0.61

765	CFDP1	Craniofacial development protein 1	0.61
766	PCK2	Phosphoenolpyruvate carboxykinase [GTP], mitochondrial	0.61
767	IPO8	Importin-8	0.61
768	LOC105372343	Uncharacterized protein	0.61
769	PHB2	Prohibitin-2	0.61
770	DDX54	ATP-dependent RNA helicase DDX54	0.61
771	MRPL13	39S ribosomal protein L13, mitochondrial	0.61
772	TTN	Titin	0.61
773	SH3BGRL3	SH3 domain-binding glutamic acid-rich-like protein 3	0.61
774	MOCS2	Molybdopterin synthase sulfur carrier subunit	0.61
775	POLR2J3	DNA-directed RNA polymerase II subunit RPB11-b2	0.61
776	WNK1	Serine/threonine-protein kinase WNK1	0.60
777	PABPC1	Polyadenylate-binding protein 1	0.60
778	RPUSD3	RNA pseudouridylate synthase domain-containing protein 3	0.60
779	IFT27	Intraflagellar transport protein 27 homolog	0.60
780	UGDH	UDP-glucose 6-dehydrogenase	0.60
781	RAB11B	Ras-related protein Rab-11B	0.60
782	RRM2	Ribonucleoside-diphosphate reductase subunit M2	0.60
783	RAP1GDS1	Rap1 GTPase-GDP dissociation stimulator 1	0.60
784	SH3D19	SH3 domain-containing protein 19	0.60
785	SNRNP40	Small Nuclear Ribonucleoprotein U5 Subunit 40	0.60
786	PAF1	RNA polymerase II-associated factor 1 homolog	0.60
787	RBBP5	Retinoblastoma-binding protein 5	0.60
788	ZCCHC11	Terminal uridylyltransferase 4	0.60
789	SMC2	Structural maintenance of chromosomes protein 2	0.60
790	SLC16A5	Monocarboxylate transporter 6	0.60
791	RRM1	Ribonucleoside-diphosphate reductase large subunit	0.60
792	STAT1	Signal transducer and activator of transcription	0.60
793	TCOF1	Treacle protein	0.60
794	DOCK2	Dedicator of cytokinesis protein 2	0.60
795	CDR2L	Cerebellar degeneration-related protein 2-like	0.60
796	SMU1	WD40 repeat-containing protein SMU1	0.59
797	RAE1	mRNA export factor	0.59
798	STAT1	Signal transducer and activator of transcription 1-alpha/beta	0.59
799	TBCD	Tubulin-specific chaperone D	0.59
800	TSR1	Pre-rRNA-processing protein TSR1 homolog	0.59
801	KDM7A	Lysine-specific demethylase 7A	0.59
802	ATP5J2-PTCD1	ATP5J2-PTCD1 readthrough	0.59
803	TWF1	Twinfilin-1	0.59
804	KIF2A	Kinesin-like protein KIF2A	0.59
805	OSBP	Oxysterol-binding protein 1	0.59
806	GULP1	PTB domain-containing engulfment adapter protein 1	0.59
807	EFHD1	EF-hand domain-containing protein D1	0.59
808	GINS2	DNA replication complex GINS protein PSF2	0.59
809	GNPDA1	Glucosamine-6-phosphate isomerase 1	0.59
810	RPRD1A	Regulation of nuclear pre-mRNA domain-containing protein 1A	0.59
811	HBS1L	HBS1-like protein	0.59
812	PSMD11	26S proteasome non-ATPase regulatory subunit 11	0.59
813	RAB11A	Ras-related protein Rab-11A	0.59
814	COX5B	Cytochrome c oxidase subunit 5B, mitochondrial	0.59
815	NOC4L	Nucleolar complex protein 4 homolog	0.59
816	DRAP1	Dr1-associated corepressor	0.59
817	PPP1R12A	Protein phosphatase 1 regulatory subunit 12A	0.59
818	FASN	Fatty acid synthase	0.59
819	RPS16	40S ribosomal protein S16	0.58

820	SND1	Staphylococcal nuclease domain-containing protein 1	0.58
821	GLA	Alpha-galactosidase A	0.58
822	CDC27	Cell division cycle protein 27 homolog	0.58
823	RPAP3	RNA polymerase II-associated protein 3	0.58
824	SLC25A44	Solute carrier family 25 member 44	0.58
825	RPL26	60S ribosomal protein L26	0.58
826	MSTO1	Protein misato homolog 1	0.58
827	PAAF1	Proteasomal ATPase-associated factor 1	0.58
828	MCCC2	Methylcrotonoyl-CoA carboxylase beta chain, mitochondrial	0.58
829	WDR70	WD repeat-containing protein 70	0.58
830	CKAP5	Cytoskeleton-associated protein 5	0.58
831	TUBB3	HCG1983504, isoform CRA_d	0.58
832	TRMT1	tRNA (guanine(26)-N(2))-dimethyltransferase	0.58
833	NUDC	Nuclear migration protein nudC	0.58
834	TRIT1	tRNA dimethylallyltransferase, mitochondrial	0.58
835	PELP1	Proline-, glutamic acid- and leucine-rich protein 1	0.58
836	FANCI	Fanconi anemia group I protein	0.58
837	NDUFA5	NADH dehydrogenase [ubiquinone] 1 alpha subcomplex subunit 5	0.58
838	DHX16	Putative pre-mRNA-splicing factor ATP-dependent RNA helicase DHX16	0.58
839	CKAP5	Cytoskeleton-associated protein 5	0.58
840	SAR1A	GTP-binding protein SAR1a	0.58
841	WDHD1	WD repeat and HMG-box DNA-binding protein 1	0.58
842	KPNA6	Importin subunit alpha-7	0.58
843	UBFD1	Ubiquitin domain-containing protein UBFD1	0.58
844	RRBP1	Ribosome-binding protein 1	0.58
845	MAPRE2	Microtubule-associated protein RP/EB family member 2	0.58
846	TP53RK	TP53-regulating kinase	0.58
847	SNCA	Alpha-synuclein	0.58
848	PELO	Protein pelota homolog	0.58
849	MICALL1	MICAL-like protein 1	0.58
850	DTYMK	Thymidylate kinase	0.58
851	UBE2Z	Ubiquitin-conjugating enzyme E2 Z	0.58
852	ATXN2	Ataxin-2	0.57
853	NOL9	Polynucleotide 5'-hydroxyl-kinase NOL9	0.57
854	TBCE	Tubulin-specific chaperone E	0.57
855	ATG3	Ubiquitin-like-conjugating enzyme ATG3	0.57
856	ATXN2L	Ataxin-2-like protein	0.57
857	LSM7	U6 snRNA-associated Sm-like protein LSm7	0.57
858	NIP7	60S ribosome subunit biogenesis protein NIP7 homolog	0.57
859	PITPNA	Phosphatidylinositol transfer protein alpha isoform	0.57
860	THOC3	THO complex subunit 3	0.57
861	IMMT	MICOS complex subunit MIC60	0.57
862	RPS13	40S ribosomal protein S13	0.57
863	PFDN5	Prefoldin subunit 5	0.57
864	PDZRN4	PDZ domain-containing RING finger protein 4	0.57
865	ELP3	Elongator complex protein 3	0.56
866	RAD18	E3 ubiquitin-protein ligase RAD18	0.56
867	ABCD3	ATP-binding cassette sub-family D member 3	0.56
868	LAS1L	Ribosomal biogenesis protein LAS1L	0.56
869	UTP14A	U3 small nucleolar RNA-associated protein 14 homolog A	0.56
870	SPEN	Msx2-interacting protein	0.56
871	FAM184B	Protein FAM184B	0.56
872	CMPK1	UMP-CMP kinase	0.56
873	PRKAR2A	cAMP-dependent protein kinase type II-alpha regulatory subunit	0.56
874	BCAP31	B-cell receptor-associated protein 31	0.56

875	FXN	Frataxin, mitochondrial	0.56
876	CACNG6	Voltage-dependent calcium channel gamma-6 subunit	0.56
877	COLGALT1	Procollagen galactosyltransferase 1	0.56
878	MED29	Intersex-like (Drosophila)	0.56
879	ZNF503	Zinc finger protein 503	0.56
880	PYCR2	Pyrroline-5-carboxylate reductase 2	0.56
881	TPM4	Tropomyosin alpha-4 chain	0.56
882	FAM98A	Protein FAM98A	0.56
883	SHCBP1L	Testicular spindle-associated protein SHCBP1L	0.56
884	BCAT1	Branched-chain-amino-acid aminotransferase, cytosolic	0.56
885	PIN4	Peptidyl-prolyl cis-trans isomerase NIMA-interacting 4	0.56
886	TXLNA	Alpha-taxilin	0.55
887	DSP	Desmoplakin	0.55
888	CFAP36	Cilia- and flagella-associated protein 36	0.55
889	NUMA1	Nuclear mitotic apparatus protein 1	0.55
890	NOB1	RNA-binding protein NOB1	0.55
891	VPS4A	Vacuolar protein sorting-associated protein 4A	0.55
892	GRIPAP1	GRIP1-associated protein 1	0.55
893	ATP6V1G1	V-type proton ATPase subunit G 1	0.55
894	NUDT15	Nucleotide triphosphate diphosphatase NUDT15	0.55
895	EEF1E1	Eukaryotic translation elongation factor 1 epsilon-1	0.55
896	TSEN34	tRNA-splicing endonuclease subunit Sen34	0.55
897	NCKAP1	Nck-associated protein 1	0.55
898	ACO1	Cytoplasmic aconitate hydratase	0.55
899	CUL2	Cullin-2	0.55
900	RGPD8	RANBP2-like and GRIP domain-containing protein 8	0.55
901	UBAP2L	Ubiquitin-associated protein 2-like	0.55
902	DDX56	DEAD (Asp-Glu-Ala-Asp) box polypeptide 56, isoform CRA_a	0.55
903	WDR19	WD repeat-containing protein 19	0.55
904	MRPS5	28S ribosomal protein S5, mitochondrial	0.55
905	PGRMC1	Membrane-associated progesterone receptor component 1	0.55
906	IPO11	Importin-11	0.55
907	CNBP	Cellular nucleic acid-binding protein	0.55
908	ATP6V1C1	V-type proton ATPase subunit C 1	0.54
909	LYPLA2	Acyl-protein thioesterase 2	0.54
910	TACC3	Transforming acidic coiled-coil-containing protein 3	0.54
911	KYAT3	Kynurenine--oxoglutarate transaminase 3	0.54
912	DHX57	Putative ATP-dependent RNA helicase DHX57	0.54
913	ANKRD1	Ankyrin repeat domain-containing protein 1	0.54
914	PFDN5	Prefoldin subunit 5	0.54
915	HAS1	Hyaluronan synthase 1	0.54
916	ENAH	Protein enabled homolog	0.54
917	NOLC1	Nucleolar and coiled-body phosphoprotein 1	0.54
918	HRAS	GTPase HRas	0.54
919	PRPF38B	Pre-mRNA-splicing factor 38B	0.54
920	DSTN	Destrin	0.54
921	APEH	Acylamino-acid-releasing enzyme	0.54
922	ASH2L	Set1/Ash2 histone methyltransferase complex subunit ASH2	0.54
923	GTF2F1	General transcription factor IIF subunit 1	0.54
924	TRIM28	Transcription intermediary factor 1-beta	0.54
925	HIBADH	3-hydroxyisobutyrate dehydrogenase, mitochondrial	0.54
926	COASY	Bifunctional coenzyme A synthase	0.54
927	PGP	Glycerol-3-phosphate phosphatase	0.54
928	EED	Polycomb protein EED	0.54
929	MRPL43	39S ribosomal protein L43, mitochondrial	0.54

930	LIMA1	LIM domain and actin-binding protein 1	0.54
931	GNB2	Guanine nucleotide-binding protein G(I)/G(S)/G(T) subunit beta-2	0.53
932	RPL21	60S ribosomal protein L21	0.53
933	PRPF6	Pre-mRNA-processing factor 6	0.53
934	CCM2L	Cerebral cavernous malformations 2 protein-like	0.53
935	CHMP2B	Charged multivesicular body protein 2b	0.53
936	HES1	Transcription factor HES-1	0.53
937	SNX1	Sorting nexin-1	0.53
938	SNX3	Sorting nexin-3	0.53
939	MESDC2	LDLR chaperone MESD	0.53
940	AP2A2	AP-2 complex subunit alpha-2	0.53
941	CPSF7	Cleavage and polyadenylation-specificity factor subunit 7	0.53
942	GEMIN4	Gem-associated protein 4	0.53
943	UBR5	E3 ubiquitin-protein ligase UBR5	0.53
944	ANXA2	Annexin A2	0.53
945	NIPSNAP1	Protein NipSnap homolog 1	0.53
946	KIF1BP	KIF1-binding protein	0.53
947	POLR1A	DNA-directed RNA polymerase subunit	0.53
948	MYO18A	Unconventional myosin-XVIIIa	0.53
949	LAGE3	EKC/KEOPS complex subunit LAGE3	0.53
950	AP1M1	AP-1 complex subunit mu-1	0.52
951	MCM4	DNA replication licensing factor MCM4	0.52
952	ARL1	ADP-ribosylation factor-like protein 1	0.52
953	SH3KBP1	SH3 domain-containing kinase-binding protein 1	0.52
954	TYMS	Thymidylate synthase	0.52
955	LASP1	LIM and SH3 domain protein 1	0.52
956	PLOD2	Procollagen-lysine,2-oxoglutarate 5-dioxygenase 2	0.52
957	AMPD2	AMP deaminase 2	0.52
958	ABCF1	ATP-binding cassette sub-family F member 1	0.52
959	NXN	Nucleoredoxin	0.52
960	GPATCH4	G patch domain-containing protein 4	0.52
961	MRPL58	Peptidyl-tRNA hydrolase ICT1, mitochondrial	0.52
962	AKAP6	A-kinase anchor protein 6	0.52
963	COIL	Coilin	0.52
964	CORO7-PAM1(	Coronin	0.52
965	TXN2	Thioredoxin, mitochondrial	0.52
966	COPZ1	Coatomer subunit zeta-1	0.52
967	TTC19	Tetratricopeptide repeat protein 19, mitochondrial	0.52
968	GIPC1	PDZ domain-containing protein GIPC1	0.52
969	NUP133	Nuclear pore complex protein Nup133	0.52
970	EMC2	ER membrane protein complex subunit 2	0.52
971	EIF3H	Eukaryotic translation initiation factor 3 subunit H	0.51
972	MAP2K1	Dual specificity mitogen-activated protein kinase kinase 1	0.51
973	CIAPIN1	Anamorsin	0.51
974	PARD3B	Partitioning defective 3 homolog B	0.51
975	ARGLU1	Arginine and glutamate-rich protein 1	0.51
976	DUS1L	tRNA-dihydrouridine(16/17) synthase [NAD(P)(+)]-like	0.51
977	NUP98	Nuclear pore complex protein Nup98-Nup96	0.51
978	IFT172	Intraflagellar transport protein 172 homolog	0.51
979	TCAF1	TRPM8 channel-associated factor 1	0.51
980	TRIP12	E3 ubiquitin-protein ligase TRIP12	0.51
981	SRPK2	SRSF protein kinase 2	0.51
982	TMED9	Transmembrane emp24 domain-containing protein 9	0.51
983	SUMO1	Small ubiquitin-related modifier 1	0.51
984	PSMG1	Proteasome assembly chaperone 1	0.51

985	ANAPC7	Anaphase-promoting complex subunit 7	0.51
986	RALA	Ras-related protein Ral-A	0.51
987	MRPL1	39S ribosomal protein L1, mitochondrial	0.50
988	TSPYL1	Testis-specific Y-encoded-like protein 1	0.50
989	SCRIB	Protein scribble homolog	0.50
990	CSTB	Cystatin-B	0.50
991	CDC123	Cell division cycle protein 123 homolog	0.50
992	TOP2B	DNA topoisomerase 2	0.50
993	RRP15	RRP15-like protein	0.50
994	PPP3CB	Serine/threonine-protein phosphatase	0.50
995	KLHL33	Kelch-like protein 33	0.50
996	MRPS11	28S ribosomal protein S11, mitochondrial	0.49
997	RPL34	60S ribosomal protein L34	0.49
998	MRPL45	39S ribosomal protein L45, mitochondrial	0.49
999	ANKS3	Ankyrin repeat and SAM domain-containing protein 3	0.49
1000	CBWD1	COBW domain-containing protein 1	0.49
1001	ATXN2L	Ataxin-2-like protein	0.49
1002	RABGGTA	Geranylgeranyl transferase type-2 subunit alpha	0.49
1003	EEF1E1	Eukaryotic translation elongation factor 1 epsilon-1	0.49
1004	SETD7	Histone-lysine N-methyltransferase	0.49
1005	ZNF593	Zinc finger protein 593	0.49
1006	TPT1	Translationally-controlled tumor protein	0.49
1007	POR	NADPH--cytochrome P450 reductase	0.49
1008	RFC1	Replication factor C subunit 1	0.49
1009	ARL8A	ADP-ribosylation factor-like protein 8A	0.49
1010	GBF1	Golgi-specific brefeldin A-resistance guanine nucleotide exchange factor 1	0.49
1011	EIF2AK2	Interferon-induced, double-stranded RNA-activated protein kinase	0.48
1012	MCCC2	Methylcrotonoyl-CoA carboxylase 2 isoform 1	0.48
1013	DIAPH1	Protein diaphanous homolog 1	0.48
1014	GTF3C4	General transcription factor 3C polypeptide 4	0.48
1015	TOX3	TOX high mobility group box family member 3	0.48
1016	RPL22L1	60S ribosomal protein L22-like 1	0.48
1017	NAA16	N-alpha-acetyltransferase 16, NatA auxiliary subunit	0.48
1018	NAPA	Alpha-soluble NSF attachment protein	0.47
1019	CYB5B	Cytochrome b5 type B	0.47
1020	PRKACA	cAMP-dependent protein kinase catalytic subunit alpha	0.47
1021	ACKR2	Atypical chemokine receptor 2	0.47
1022	EPB41L2	Band 4.1-like protein 2	0.47
1023	SAAL1	Protein SAAL1	0.47
1024	TRAPPC3	Trafficking protein particle complex subunit 3	0.47
1025	PYROXD2	Pyridine nucleotide-disulfide oxidoreductase domain-containing protein 2	0.47
1026	ISYNA1	Inositol-3-phosphate synthase 1	0.47
1027	NUCB2	Nucleobindin 2, isoform CRA_b	0.47
1028	SF3B6	Splicing factor 3B subunit 6	0.46
1029	PRKACB	cAMP-dependent protein kinase catalytic subunit beta	0.46
1030	CPE	Carboxypeptidase E	0.46
1031	RPS26P11	Putative 40S ribosomal protein S26-like 1	0.46
1032	TBC1D15	TBC1 domain family member 15	0.46
1033	TELO2	Telomere length regulation protein TEL2 homolog	0.46
1034	NME7	Nucleoside diphosphate kinase 7	0.46
1035	BAG6	BCL2 Associated Athanogene 6	0.46
1036	NGDN	Neuroguidin	0.46
1037	RANBP10	Ran-binding protein 10	0.46
1038	MYO5A	Unconventional myosin-Va	0.46
1039	PCNT	Pericentrin	0.46

1040	GPD2	Glycerol-3-phosphate dehydrogenase, mitochondrial	0.46
1041	CKAP4	Cytoskeleton-associated protein 4	0.46
1042	NECAP1	Adaptin ear-binding coat-associated protein 1	0.45
1043	UBC	Polyubiquitin-C	0.45
1044	ASS1	Argininosuccinate synthase	0.45
1045	TTC1	Tetratricopeptide repeat protein 1	0.45
1046	DHCR24	Delta(24)-sterol reductase	0.45
1047	CTNND1	Catenin delta-1	0.45
1048	BZW2	Basic leucine zipper and W2 domain-containing protein 2	0.45
1049	TRIM33	E3 ubiquitin-protein ligase TRIM33	0.45
1050	AKT1S1	Proline-rich AKT1 substrate 1	0.45
1051	UQCRFS1	Cytochrome b-c1 complex subunit Rieske, mitochondrial	0.45
1052	EPB41L3	Band 4.1-like protein 3	0.44
1053	LXN	Latexin	0.44
1054	IMPDH1	Inosine-5'-monophosphate dehydrogenase	0.44
1055	DPCD	Protein DPCD	0.44
1056	DLAT	Dihydrolipoyllysine-residue acetyltransferase component of pyruvate dehydrogenase c	0.44
1057	NDUFA9	NADH dehydrogenase [ubiquinone] 1 alpha subcomplex subunit 9, mitochondrial	0.44
1058	RAB18	Ras-related protein Rab-18	0.44
1059	C18orf25	Uncharacterized protein C18orf25	0.44
1060	ARFIP1	Arfaptin-1	0.44
1061	PLK1	Serine/threonine-protein kinase PLK1	0.44
1062	RPS6KA3	Ribosomal protein S6 kinase alpha-3	0.43
1063	RPF2	Ribosome production factor 2 homolog	0.43
1064	RIF1	Telomere-associated protein RIF1	0.43
1065	ECHDC1	Ethylmalonyl-CoA decarboxylase	0.43
1066	HSPBP1	Hsp70-binding protein 1	0.43
1067	ADPRHL2	Poly(ADP-ribose) glycohydrolase ARH3	0.43
1068	CHD3	Chromodomain-helicase-DNA-binding protein 3	0.42
1069	KIFC3	Kinesin-like protein KIFC3	0.42
1070	UBE2C	Ubiquitin-conjugating enzyme E2	0.42
1071	TUBG1	Tubulin gamma-1 chain	0.42
1072	NFATC4	Nuclear factor of activated T-cells, cytoplasmic 4	0.42
1073	ARHGAP1	Rho GTPase-activating protein 1	0.42
1074	PRPF3	U4/U6 small nuclear ribonucleoprotein Prp3	0.42
1075	FAH	Fumarylacetoacetase	0.42
1076	QKI	Protein quaking	0.42
1077	ARPC3	Actin-related protein 2/3 complex subunit 3	0.41
1078	NMD3	60S ribosomal export protein NMD3	0.41
1079	FADD	FAS-associated death domain protein	0.41
1080	DYNLL1	Dynein light chain 1, cytoplasmic	0.41
1081	NCF2	Neutrophil cytosol factor 2	0.41
1082	CRK	Adapter molecule crk	0.41
1083	NIF3L1	NIF3-like protein 1	0.41
1084	EFHD2	EF-hand domain-containing protein D2	0.40
1085	FAM117B	Protein FAM117B	0.40
1086	MCMBP	Mini-chromosome maintenance complex-binding protein	0.40
1087	MRPL11	39S ribosomal protein L11, mitochondrial	0.40
1088	STRC	Stereocilin	0.39
1089	STAT3	Signal transducer and activator of transcription	0.39
1090	SLC39A11	Zinc transporter ZIP11	0.39
1091	CCDC12	Coiled-coil domain containing 12, isoform CRA_a	0.39
1092	AGPS	Alkylglycerone-phosphate synthase	0.39
1093	MID1	E3 ubiquitin-protein ligase Midline-1	0.39
1094	TEX33	Testis-expressed protein 33	0.39

1095	MBIP	MAP3K12-binding inhibitory protein 1	0.38
1096	PRX	Periaxin	0.38
1097	UQCRC1	Cytochrome b-c1 complex subunit 1, mitochondrial	0.38
1098	MAGEB4	Melanoma-associated antigen B4	0.38
1099	LBR	Lamin-B receptor	0.38
1100	AACS	Acetoacetyl-CoA synthetase	0.38
1101	CDK7	Cyclin-dependent kinase 7	0.38
1102	DDX56	Probable ATP-dependent RNA helicase DDX56	0.38
1103	PTDSS1	Phosphatidylserine synthase 1	0.38
1104	FAM186A	Protein FAM186A	0.38
1105	GNAO1	Guanine nucleotide-binding protein G(o) subunit alpha	0.38
1106	USP19	Ubiquitin carboxyl-terminal hydrolase 19	0.37
1107	ZNF346	Zinc finger protein 346	0.37
1108	TRPT1	tRNA 2'-phosphotransferase 1	0.37
1109	NVL	Nuclear valosin-containing protein-like	0.37
1110	PRKAR1A	cAMP-dependent protein kinase type I-alpha regulatory subunit	0.36
1111	PLAA	Phospholipase A-2-activating protein	0.36
1112	SIRT5	NAD-dependent protein deacylase sirtuin-5, mitochondrial	0.36
1113	PDXDC1	Pyridoxal-dependent decarboxylase domain-containing protein 1	0.36
1114	TRNT1	CCA tRNA nucleotidyltransferase 1, mitochondrial	0.36
1115	MAP7	Ensconsin	0.35
1116	HGH1	Protein HGH1 homolog	0.35
1117	MRFAP1	MORF4 family-associated protein 1	0.35
1118	PRR3	Proline-rich protein 3	0.35
1119	RNF181	E3 ubiquitin-protein ligase RNF181	0.34
1120	POLR2B	DNA-directed RNA polymerase subunit beta	0.34
1121	MAGOH	Protein mago nashi homolog	0.34
1122	EIF1AD	Probable RNA-binding protein EIF1AD	0.34
1123	UPF3B	Regulator of nonsense transcripts 3B	0.34
1124	POLR2I	DNA-directed RNA polymerase II subunit RPB9	0.34
1125	FAU	40S ribosomal protein S30	0.33
1126	TXNL1	Thioredoxin-like protein 1	0.33
1127	RPN2	Dolichyl-diphosphooligosaccharide--protein glycosyltransferase subunit 2	0.33
1128	PACS1	Phosphofurin acidic cluster sorting protein 1	0.33
1129	TIMM8B	Mitochondrial import inner membrane translocase subunit Tim8 B	0.32
1130	NAGK	N-acetyl-D-glucosamine kinase	0.32
1131	RANGRF	Ran guanine nucleotide release factor	0.32
1132	SUCLG2	Succinate--CoA ligase [GDP-forming] subunit beta, mitochondrial	0.31
1133	HELLS	Helicase, lymphoid-specific, isoform CRA_d	0.31
1134	MEA1	Male-enhanced antigen 1	0.31
1135	SNAP23	Synaptosomal-associated protein	0.30
1136	ATP5J	ATP synthase-coupling factor 6, mitochondrial	0.30
1137	TAB1	TGF-beta-activated kinase 1 and MAP3K7-binding protein 1	0.29
1138	OGT	UDP-N-acetylglucosamine--peptide N-acetylglucosaminyltransferase 110 kDa subuni	0.29
1139	RAD23A	UV excision repair protein RAD23 homolog A	0.29
1140	C2	Complement C2	0.28
1141	MAPK7	Mitogen-activated protein kinase 7	0.28
1142	GYG1	Glycogenin 1, isoform CRA_e	0.28
1143	CENPC	Centromere protein C	0.28
1144	MKLN1	Muskelin	0.28
1145	NCAPG2	Condensin-2 complex subunit G2	0.27
1146	LIG3	DNA ligase 3	0.27
1147	SNX17	Sorting nexin-17	0.27
1148	PPP6R3	Serine/threonine-protein phosphatase 6 regulatory subunit 3	0.27
1149	PPIC	Peptidyl-prolyl cis-trans isomerase C	0.25

1150	HEXB	Beta-hexosaminidase subunit beta	0.25
1151	GADD45GIP1	Growth arrest and DNA damage-inducible proteins-interacting protein 1	0.25
1152	VSTM2A	V-set and transmembrane domain-containing protein 2A	0.25
1153	BIRC5	Baculoviral IAP repeat-containing protein 5	0.24
1154	RPS19	40S ribosomal protein S19	0.24
1155	MRPS21	28S ribosomal protein S21, mitochondrial	0.24
1156	TRIM21	E3 ubiquitin-protein ligase TRIM21	0.24
1157	ASCC2	Activating signal cointegrator 1 complex subunit 2	0.23
1158	AHCYL1	Adenosylhomocysteinase 2	0.22
1159	STK4	Serine/threonine-protein kinase 4	0.22
1160	TOM1	Target of Myb protein 1	0.22
1161	LUZP1	Leucine zipper protein 1	0.22
1162	FAM208A	Protein TASOR	0.22
1163	MORF4L2	Mortality factor 4-like protein 2	0.21
1164	TRMT5	tRNA (guanine(37)-N1)-methyltransferase	0.21
1165	KIAA0556	Protein KIAA0556	0.21
1166	BUD31	Protein BUD31 homolog	0.21
1167	HEXB	Beta-hexosaminidase subunit beta	0.19
1168	RPL29	60S ribosomal protein L29	0.19
1169	CROCC	Rootletin	0.18
1170	LIPE	Hormone-sensitive lipase	0.17
1171	SAV1	Protein salvador homolog 1	0.16
1172	MRPL27	39S ribosomal protein L27, mitochondrial	0.16
1173	FAM162A	Protein FAM162A	0.15
1174	SNW1	SNW domain-containing protein 1	0.15
1175	TAF3	Transcription initiation factor TFIID subunit 3	0.13
1176	TAF1L	Transcription initiation factor TFIID subunit 1-like	0.12
1177	IRGQ	Immunity-related GTPase family Q protein	0.11
1178	PDDC1	Parkinson disease 7 domain-containing protein 1	0.10
1179	FBXW8	F-box/WD repeat-containing protein 8	0.10
1180	NRBP1	Nuclear receptor-binding protein	0.10
1181	CHRDL1	Chordin-like protein 1	0.09
1182	GLTSCR2	Glioma tumor suppressor candidate region gene 2 protein	0.07
1183	ANXA1	Annexin A1	0.06
1184	OR4A15	Olfactory receptor 4A15	0.05
1185	IFT81	Intraflagellar transport protein 81 homolog	0.03

Studies of glyoxalase 1-linked multidrug resistance reveal glycolysis-derived reactive metabolite, methylglyoxal, is a common contributor in cancer chemotherapy targeting the spliceosome.

Muhanad Alhujaily et al.

Table S3. Proteins of the cytoplasmic extract containing MG-H1 residues and increased by HEK293 cells treatment with methylglyoxal

No	Gene	Name of protein	Fold change
1	MEGF11	Multiple epidermal growth factor-like domains protein 11	14.0
2	TSTA3	GDP-L-fucose synthase	5.7
3	NLRP2	NACHT, LRR and PYD domains-containing protein 2	4.1
4	TRIM52	Tripartite motif-containing protein 52	3.6
5	CHMP2A	Charged multivesicular body protein 2a	3.5
6	GPR119	Glucose-dependent insulinotropic receptor	3.4
7	KRT9	Keratin, type I cytoskeletal 9	3.3
8	ABHD12B	Alpha/Beta Hydrolase Domain-Containing Protein 12B	3.2
9	CACNA1H	Voltage-dependent T-type calcium channel subunit alpha-1H	3.2
10	SV2B	Synaptic vesicle glycoprotein 2B	3.2
11	THRA	Thyroid hormone receptor alpha	2.7
12	KIAA0355	Uncharacterized protein KIAA0355	2.6
13	FAAP20	Fanconi anemia core complex-associated protein 20	2.6
14	REXO2	Oligoribonuclease, mitochondrial	2.4
15	CIITA	MHC class II transactivator	2.4
16	MZT2A	Mitotic-spindle organizing protein 2A	2.3
17	PTPRN2	Receptor-type tyrosine-protein phosphatase N2	2.1
18	SLC25A37	Mitoferrin-1	2.1
19	TUBGCP6	Gamma-tubulin complex component 6	2.0
20	CLN5	Ceroid-lipofuscinosis neuronal protein 5	2.0
21	MACF1	Microtubule-actin cross-linking factor 1, isoforms 1/2/3/5	2.0
22	COL27A1	Collagen alpha-1(XXVII) chain	2.0
23	PLCB1	Phosphoinositide phospholipase C	1.9
24	C19orf68	Uncharacterized protein C19orf68	1.9
25	SEMA4B	Semaphorin-4B	1.8
26	CABIN1	Calcineurin-binding protein cabin-1	1.8
27	SLC6A9	Fanconi Anemia Core Complex Associated Protein 20	1.7
28	MYEF2	Myelin expression factor 2	1.7
29	MUC20	Mucin-20	1.6
30	SAMD11	Sterile alpha motif domain-containing protein 11	1.6
31	CEP250	Centrosome-associated protein CEP250	1.6
32	WDR33	pre-mRNA 3' end processing protein WDR33	1.6
33	PPP4R1L	Putative serine/threonine-protein phosphatase 4 regulatory subunit 1-like	1.5
34	SNTG2	Gamma-2-syntrophin	1.5
35	DAP3	28S ribosomal protein S29, mitochondrial	1.5
36	WNK3	Serine/threonine-protein kinase WNK3	1.4
37	EDC3	Enhancer of mRNA-decapping protein 3	1.4
38	FAM27E3	Family With Sequence Similarity 27 Member E3	1.3
39	SMG6	Telomerase-binding protein EST1A	1.3
40	POM121C	Nuclear envelope pore membrane protein POM 121C	1.2
41	NASP	Nuclear autoantigenic sperm protein	1.2

Studies of glyoxalase 1-linked multidrug resistance reveal glycolysis-derived reactive metabolite, methylglyoxal, is a common contributor in cancer chemotherapy targeting the spliceosome.

Muhanad Alhujaily et al.

Table S4. Proteins of the cytoplasmic extract containing MG-H1 residues and decreased by HEK293 cells treatment with methylglyoxal

No	Gene	Name of protein	Fold change
1	HNRNPK	Heterogeneous nuclear ribonucleoprotein K	0.91
2	FUS	RNA-binding protein FUS	0.86
3	ZCCHC2	Zinc finger CCHC domain-containing protein 2	0.84
4	DHX15	Pre-mRNA-splicing factor ATP-dependent RNA helicase	0.82
5	TRIM46	Tripartite motif-containing protein 46	0.81
6	CSNK2A2	Casein kinase II subunit alpha'	0.81
7	HNRNPM	Heterogeneous nuclear ribonucleoprotein M	0.81
8	EWSR1	RNA-binding protein EWS	0.81
9	KRI1	Protein KRI1 homolog	0.79
10	LDHA	L-lactate dehydrogenase A chain	0.79
11	ESPNL	Espin-like protein	0.78
12	MSH6	DNA mismatch repair protein Msh6	0.78
13	ILF2	Interleukin enhancer-binding factor 2	0.76
14	PRDX1	Peroxiredoxin-1	0.76
15	MAK16	Protein MAK16 homolog OS	0.76
16	HNRNPU	Heterogeneous nuclear ribonucleoprotein U	0.75
17	RECQL5	ATP-dependent DNA helicase	0.75
18	EEF2	Elongation factor 2	0.73
19	HIST1H2BN	Histone H2B	0.73
20	BAZ1B	Tyrosine-protein kinase BAZ1B	0.71
21	ATXN10	Ataxin-10	0.71
22	PHLPP1	PH domain leucine-rich repeat-containing protein phosphatase 1	0.70
23	SUPT6H	Transcription elongation factor SPT6	0.68
24	KCTD9	BTB/POZ domain-containing protein KCTD9	0.67
25	CPXM2	Inactive carboxypeptidase-like protein X2	0.67
26	USP5	Ubiquitin carboxyl-terminal hydrolase 5	0.66
27	ZFR	Zinc finger RNA-binding protein	0.66
28	NPIPA2	Nuclear pore complex-interacting protein family member A2	0.64
29	MYO15A	Unconventional myosin-XV	0.62
30	AGBL4	Cytosolic carboxypeptidase 6	0.62
31	HAGH	Hydroxyacylglutathione hydrolase, mitochondrial	0.62
32	ZNF513	Zinc finger protein 513	0.62
33	ZNF345	Zinc finger protein 345	0.61
34	SRP54	Signal recognition particle 54 kDa protein	0.61
35	UBA52	Ubiquitin-60S ribosomal protein L40	0.61
36	MYD88	Myeloid differentiation primary response protein MyD88	0.61
37	LOC105372343	Uncharacterized protein	0.61
38	RPUSD3	RNA pseudouridylate synthase domain-containing protein 3	0.60
39	DOCK2	Dedicator of cytokinesis protein 2	0.60
40	TRIT1	tRNA dimethylallyltransferase, mitochondrial	0.58
41	SPEN	Msx2-interacting protein	0.56
42	DSP	Desmoplakin	0.55
43	ANKRD1	Ankyrin repeat domain-containing protein 1	0.54
44	POLR1A	DNA-directed RNA polymerase subunit	0.53
45	MYO18A	Unconventional myosin-XVIIIa	0.53

46	AKAP6	A-kinase anchor protein 6	0.52
47	PARD3B	Partitioning defective 3 homolog B	0.51
48	IFT172	Intraflagellar transport protein 172 homolog	0.51
49	TOP2B	DNA topoisomerase 2	0.50
50	KLHL33	Kelch-like protein 33	0.50
51	ANKS3	Ankyrin repeat and SAM domain-containing protein 3	0.49
52	ACKR2	Atypical chemokine receptor 2	0.47
53	CPE	Carboxypeptidase E	0.46
54	GPD2	Glycerol-3-phosphate dehydrogenase, mitochondrial	0.46
55	CHD3	Chromodomain-helicase-DNA-binding protein 3	0.42
56	SLC39A11	Zinc transporter ZIP11	0.39
57	TEX33	Testis-expressed protein 33	0.39
58	MBIP	MAP3K12-binding inhibitory protein 1	0.38
59	PRX	Periaxin	0.38
60	MAP7	Ensconsin	0.35
61	VSTM2A	V-set and transmembrane domain-containing protein 2A	0.25
62	MRPS21	28S ribosomal protein S21, mitochondrial	0.24
63	TRIM21	E3 ubiquitin-protein ligase TRIM21	0.24
64	KIAA0556	Protein KIAA0556	0.21
65	CROCC	Rootletin	0.18
66	LIPE	Hormone-sensitive lipase	0.17
67	TAF3	Transcription initiation factor TFIID subunit 3	0.13
68	FBXW8	F-box/WD repeat-containing protein 8	0.10
69	OR4A15	Olfactory receptor 4A15	0.05

Studies of glyoxalase 1-linked multidrug resistance reveal glycolysis-derived reactive metabolite, methylglyoxal, is a common contributor in cancer chemotherapy targeting the spliceosome.

Muhanad Alhujaily et al.

Table S5. Proteins in the nuclear extract increased in abundance by treatment with methylglyoxal.

No	Gene	Name of protein	Fold change
1	MLXIPL	Carbohydrate-responsive element-binding protein	262.6
2	ATXN7L1	Ataxin-7-like protein 1	216.1
3	PPBP	Platelet basic protein	48.3
4	IGHG2	Immunoglobulin heavy constant gamma 2	23.8
5	TAC1	Protachykinin-1	20.6
6	L1TD1	LINE-1 type transposase domain-containing protein 1	19.5
7	RBM10	RNA binding motif protein 10, isoform CRA_d	19.1
8	KNG1	Kininogen-1	17.3
9	ACAA2	3-ketoacyl-CoA thiolase, mitochondrial	14.7
10	IGHA2	Ig alpha-2 chain C region	14.5
11	HPX	HPX protein	13.9
12	HRG	Histidine-rich glycoprotein	11.3
13	PF4	Platelet factor 4	10.9
14	APOA1	Apolipoprotein A-I	9.6
15	IGHG1	Ig gamma-1 chain C region	9.2
16	TF	Transferrin	8.5
17	IGHG3	Immunoglobulin heavy constant gamma 3	7.8
18	DPH1	Diphthamide biosynthesis protein 1	6.7
19	A2M	Alpha-2-macroglobulin	6.3
20	PRRC1	Protein PRRC1 (Proline-rich and coiled-coil-containing protein 1)	6.2
21	ALB	Albumin	6.2
22	EIF4EBP1	Eukaryotic translation initiation factor 4E-binding protein 1	5.4
23	MRPL28	39S ribosomal protein L28, mitochondrial	4.7
24	NCF2	Neutrophil cytosol factor 2	4.0
25	AHSA1	Activator of 90 kDa heat shock protein ATPase homolog 1	3.0
26	WDR66	WD repeat-containing protein 66	2.9
27	CNPY3	Protein canopy homolog 3	2.8
28	CHTOP	Chromatin target of PRMT1 protein	2.7
29	SUCLG2	Succinate--CoA ligase [GDP-forming] subunit beta, mitochondrial	2.3
30	RWDD1	RWD domain-containing protein 1	2.2
31	SCAMP3	Secretory carrier-associated membrane protein 3	2.2
32	CCDC158	Coiled-coil domain-containing protein 158	2.1
33	ECHS1	Enoyl-CoA hydratase, mitochondrial	2.0
34	SLC9A3R1	Na(+)/H(+) exchange regulatory cofactor NHE-RF1	1.9
35	POTEE	POTE ankyrin domain family member E	1.8
36	NENF	Neudesin	1.6
37	PDIA3	Protein disulfide-isomerase A3	1.6
38	ACTB	Actin, cytoplasmic 1	1.6
39	ACTBL2	Beta-actin-like protein 2	1.6
40	IDE	Insulin-degrading enzyme	1.5
41	CAPZA1	F-actin-capping protein subunit alpha-1	1.5
42	ACTC1	Actin, alpha cardiac muscle 1	1.5
43	PTPN1	Tyrosine-protein phosphatase non-receptor type	1.5
44	HSPA8	Heat shock cognate 71 kDa protein	1.4
45	PSAP	Prosaposin	1.3

46	MMP25	Matrix metalloproteinase-25	1.3
47	COX17	Cytochrome c oxidase copper chaperone	1.2
48	GAA	Lysosomal alpha-glucosidase	1.2

Studies of glyoxalase 1-linked multidrug resistance reveal glycolysis-derived reactive metabolite, methylglyoxal, is a common contributor in cancer chemotherapy targeting the spliceosome.

Muhanad Alhujaily et al.

Table S6. Proteins in the nuclear extract decreased in abundance by treatment with methylglyoxal.

No	Gene	Name of protein	Fold change
1	XRCC5	X-ray repair cross-complementing protein 5	0.78
2	PYCR2	Pyrroline-5-carboxylate reductase	0.77
3	MAGOHB	Protein mago nashi homolog 2	0.72
4	CPSF1	Cleavage and polyadenylation specificity factor subunit 1	0.71
5	HNRNPUL1	Heterogeneous nuclear ribonucleoprotein U-like protein 1	0.67
6	CIRBP	Cold-inducible RNA-binding protein	0.67
7	4SV	Uncharacterized protein	0.67
8	SNRPA1	U2 small nuclear ribonucleoprotein A'	0.66
9	RPS5	40S ribosomal protein S5	0.64
10	PCBP2	Poly(rC)-binding protein 2	0.63
11	FAM136A	Family With Sequence Similarity 136 Member A	0.61
12	RPL36A	60S ribosomal protein L36a	0.60
13	ZFR	Zinc finger RNA-binding protein	0.60
14	PHC2	Polyhomeotic-like protein 2	0.59
15	NIP7	60S ribosome subunit biogenesis protein NIP7 homolog	0.58
16	TEX15	Testis-expressed protein 15	0.57
17	FAM50A	Protein FAM50A	0.56
18	GLO1	Glyoxalase 1	0.56
19	SNRPD1	Small nuclear ribonucleoprotein Sm D1	0.56
20	PTBP1	Polypyrimidine tract-binding protein 1	0.55
21	PYCR1	Pyrroline-5-carboxylate reductase 1, mitochondrial	0.50
22	PRMT1	Protein arginine N-methyltransferase 1	0.50
23	ODF3L2	Outer dense fiber protein 3-like protein 2	0.50
24	ARHGAP4	Rho GTPase-activating protein 4	0.49
25	RPL28	60S ribosomal protein L28 OS	0.49
26	MAZ	Myc-associated zinc finger protein	0.47
27	SUB1	Activated RNA polymerase II transcriptional coactivator p15	0.47
28	FSHR	Follicle-stimulating hormone receptor	0.47
29	PUS1	tRNA pseudouridine synthase	0.46
30	DKC1	H/ACA ribonucleoprotein complex subunit 4	0.44
31	NAT10	RNA cytidine acetyltransferase	0.43
32	CHD8	Chromodomain-helicase-DNA-binding protein 8	0.43
33	SF3B4	Splicing factor 3B subunit 4	0.42
34	HADH	Hydroxyacyl-coenzyme A dehydrogenase, mitochondrial	0.42
35	STK38L	Serine/threonine-protein kinase 38-like	0.42
36	MAP4	Microtubule-associated protein	0.41
37	BCKDHB	2-oxoisovalerate dehydrogenase subunit beta, mitochondrial	0.40
38	CETN2	Centrin-2	0.39
39	PRRC2B	Protein PRRC2B (Proline-rich coiled-coil protein 2B)	0.38
40	NIF3L1BP1	Ngg1 interacting factor 3 like 1 binding protein 1, isoform CRA_b	0.37
41	CBR4	Carbonyl reductase family member 4	0.36
42	C19orf53	Leydig cell tumor 10 kDa protein homolog	0.36
43	LYST	Lysosomal-trafficking regulator	0.34
44	LMNTD2	Lamin tail domain-containing protein 2	0.31
45	TERF2IP	Telomeric repeat-binding factor 2-interacting protein 1	0.30

46	ATPIF1	ATPase inhibitor, mitochondrial	0.29
47	RPS27L	40S ribosomal protein S27	0.26
48	HMGA2	High mobility group protein HMGI-C	0.20
49	THOC5	THO complex subunit 5 homolog	0.20
50	ITPRIPL2	Inositol 1,4,5-trisphosphate receptor-interacting protein-like 2	0.19
51	LSM7	U6 snRNA-associated Sm-like protein LSm7	0.18
52	AASS	Alpha-aminoadipic semialdehyde synthase, mitochondrial	0.17
53	GTF2E2	Transcription initiation factor IIE subunit beta	0.17
54	GYS1	Glycogen [starch] synthase, muscle	0.16
55	GCDH	Glutaryl-CoA dehydrogenase, mitochondrial	0.13
56	ABHD14A	Alpha/beta hydrolase domain-containing protein 14A	0.09
57	PSMD7	26S proteasome non-ATPase regulatory subunit 7	0.08
58	RBM3	RNA-binding protein 3	0.02

Studies of glyoxalase 1-linked multidrug resistance reveal glycolysis-derived reactive metabolite, methylglyoxal, is a common contributor in cancer chemotherapy targeting the spliceosome.

Muhanad Alhujaily et al.

Table S7. Proteins in the mitochondrial matrix and intermembrane space extract increased in abundance by treatment with methylglyoxal.

No	Gene	Name of protein	Fold change
1	CRYAB	crystallin alpha B	49.3
2	TERF2	telomeric repeat binding factor 2	27.3
3	ALDH3A1	aldehyde dehydrogenase 3 family member A1	20.6
4	KRT5	keratin 5	18.4
5	SLC26A10	solute carrier family 26 member 10	10.7
6	SAFB2	scaffold attachment factor B2	8
7	MTSS1	MTSS1, I-BAR domain containing	6.8
8	IRAK4	interleukin 1 receptor associated kinase 4	6.6
9	CALML5	calmodulin like 5	4.1
10	CASP14	caspase 14	3
11	MYL6	myosin light chain 6	2
12	RPL21	ribosomal protein L21	1.9
13	RPL3	ribosomal protein L3	1.7
14	RNPS1	RNA binding protein with serine rich domain 1	1.7
15	RPL3	ribosomal protein L3	1.6
16	SOD1	superoxide dismutase 1, soluble	1.4

Studies of glyoxalase 1-linked multidrug resistance reveal glycolysis-derived reactive metabolite, methylglyoxal, is a common contributor in cancer chemotherapy targeting the spliceosome.
Muhanad Alhujaily et al.

Table S8. Proteins in the mitochondrial matrix and intermembrane space extract decreased in abundance by treatment with methylglyoxal.

No	Gene	Name of protein	Fold change
1	NAP1L4	Nucleosome assembly protein 1-like 4 OS	0.79
2	PTPN11	Tyrosine-protein phosphatase non-receptor type 11 OS	0.75
3	METAP2	Methionine aminopeptidase 2 OS	0.74
4	RANBP1	Ran-specific GTPase-activating protein OS	0.74
5	NUMA1	Nuclear mitotic apparatus protein 1 OS	0.73
6	NAP1L1	Nucleosome assembly protein 1-like 1 (Fragment) OS	0.72
7	ANP32B	Acidic leucine-rich nuclear phosphoprotein 32 family member B OS	0.71
8	NAP1L1	Nucleosome assembly protein 1-like 1 OS	0.71
9	WDR12	Ribosome biogenesis protein WDR12 OS	0.71
10	MAD2L1	Mitotic spindle assembly checkpoint protein MAD2A OS	0.70
11	UCHL3	Ubiquitin carboxyl-terminal hydrolase OS	0.69
12	GIGYF2	PERQ amino acid-rich with GYF domain-containing protein 2 OS	0.68
13	NAPA	Alpha-soluble NSF attachment protein OS	0.67
14	EIF5A	Eukaryotic translation initiation factor 5A (Fragment) OS	0.67
15	CCT8	T-complex protein 1 subunit theta OS	0.67
16	RNASEH2A	Ribonuclease H2 subunit A OS	0.67
17	API5	Apoptosis inhibitor 5 OS	0.66
18	NAE1	NEDD8-activating enzyme E1 regulatory subunit OS	0.66
19	NELFE	Negative elongation factor E OS	0.65
20	TRMT6	tRNA (adenine(58)-N(1))-methyltransferase non-catalytic subunit TRM6 OS	0.65
21	PLS3	Plastin-3 OS	0.65
22	PAFAH1B3	Platelet-activating factor acetylhydrolase IB subunit gamma (Fragment) OS	0.62
23	NMD3	60S ribosomal export protein NMD3 OS	0.62
24	OSBP	Oxysterol-binding protein 1 OS	0.61
25	ACP1	Low molecular weight phosphotyrosine protein phosphatase OS	0.61
26	ARHGDIA	Rho GDP-dissociation inhibitor 1 (Fragment) OS	0.60
27	UBTF	Nucleolar transcription factor 1 OS	0.60
28	EIF2B2	Translation initiation factor eIF-2B subunit beta OS	0.59
29	SRPK2	SRSF protein kinase 2 OS	0.59
30	KHSRP	Far upstream element-binding protein 2 OS	0.59
31	JMJD6	Bifunctional arginine demethylase and lysyl-hydroxylase JMJD6 OS	0.59
32	APOA1	Apolipoprotein A-I OS	0.59
33	SMARCC1	SWI/SNF complex subunit SMARCC1 OS	0.58
34	C14orf166	UPF0568 protein C14orf166 OS	0.58
35	ECM29	Proteasome-associated protein ECM29 homolog OS	0.57
36	GSTM3	Glutathione S-transferase OS	0.57
37	TROVE2	60 kDa SS-A/Ro ribonucleoprotein (Fragment) OS	0.57
38	CLUH	Clustered mitochondria protein homolog (Fragment) OS	0.56
39	KLC1	Kinesin light chain 1 OS	0.56
40	CCDC124	Coiled-coil domain-containing protein 124 OS	0.55
41	CCDC43	CCDC43 protein OS	0.55
42	VIM	Vimentin OS	0.55
43	TCERG1	Transcription elongation regulator 1 OS	0.54
44	VTA1	Chromosome 6 open reading frame 55, isoform CRA_b OS	0.54
45	APIP	Methylthioribulose-1-phosphate dehydratase OS	0.54

46	ZC3H15	Zinc finger CCCH domain-containing protein 15 OS	0.54
47	DNAJC2	DnaJ homolog subfamily C member 2 OS	0.54
48	STAT1	Signal transducer and activator of transcription OS	0.54
49	PRPF31	U4/U6 small nuclear ribonucleoprotein Prp31 OS	0.54
50	CLUH	Clustered mitochondria protein homolog OS	0.54
51	ARMT1	Protein-glutamate O-methyltransferase OS	0.53
52	CNDP2	Cytosolic non-specific dipeptidase OS	0.53
53	EDC4	Enhancer of mRNA-decapping protein 4 OS	0.52
54	SURF2	Surfeit locus protein 2 OS	0.52
55	SNX5	Sorting nexin-5 OS	0.51
56	IFT88	Intraflagellar transport protein 88 homolog (Fragment) OS	0.51
57	TFG	Protein TFG (Fragment) OS	0.51
58	GLOD4	Glyoxalase domain-containing protein 4 OS	0.50
59	ATXN7L3B	Putative ataxin-7-like protein 3B OS	0.49
60	EAPP	E2F-associated phosphoprotein OS	0.48
61	PKN2	Serine/threonine-protein kinase N2 OS	0.48
62	CTTN	Src substrate cortactin OS	0.47
63	GTPBP1	GTP-binding protein 1 OS	0.47
64	PPIA	Peptidyl-prolyl cis-trans isomerase A	0.47
65	CBSL	Cystathionine beta-synthase-like protein OS	0.46
66	RABGGTB	Geranylgeranyl transferase type-2 subunit beta OS	0.46
67	UBE2G1	Ubiquitin-conjugating enzyme E2 G1 OS	0.46
68	TST	Thiosulfate sulfurtransferase OS	0.45
69	ADI1	1,2-dihydroxy-3-keto-5-methylthiopentene dioxygenase OS	0.45
70	NUP133	Nuclear pore complex protein Nup133 OS	0.44
71	HSD17B4	Peroxisomal multifunctional enzyme type 2 OS	0.44
72	DDX55	RNA helicase OS	0.44
73	HSPB11	Chromosome 1 open reading frame 41, isoform CRA_b OS	0.43
74	GINS1	DNA replication complex GINS protein PSF1 OS	0.43
75	RIOK1	Serine/threonine-protein kinase RIO1 OS	0.42
76	DSP	Desmoplakin OS	0.42
77	PREP	Prolyl endopeptidase OS	0.42
78	ARL6IP4	ADP-ribosylation factor-like protein 6-interacting protein 4 OS	0.42
79	CDC123	Cell division cycle protein 123 homolog OS	0.42
80	GRB2	Growth factor receptor-bound protein 2 OS	0.41
81	EIF1	Eukaryotic translation initiation factor 1 OS	0.41
82	BLVRA	Biliverdin reductase A OS	0.40
83	NT5C	5'(3')-deoxyribonucleotidase, cytosolic type OS	0.40
84	RABL6	Rab-like protein 6 OS	0.40
85	CWC27	Peptidyl-prolyl cis-trans isomerase CWC27 homolog OS	0.39
86	HNRNPH3	Heterogeneous nuclear ribonucleoprotein H3 OS	0.39
87	TXNRD1	Thioredoxin reductase 1, cytoplasmic OS	0.39
88	AFM	Afamin OS	0.39
89	PHF6	PHD finger protein 6 OS	0.39
90	NLN	Neurolysin, mitochondrial OS	0.39
91	PGM1	Phosphoglucomutase-1 OS	0.38
92	GTF3C5	General transcription factor 3C polypeptide 5 OS	0.38
93	POLR1C	DNA-directed RNA polymerases I and III subunit RPAC1 OS	0.37
94	ACTN2	Alpha-actinin-2 OS	0.36
95	STAU1	Double-stranded RNA-binding protein Staufen homolog 1 OS	0.36
96	POLR2G	DNA-directed RNA polymerase II subunit RPB7 OS	0.36

97	VPS29	Vacuolar protein sorting-associated protein 29 OS	0.36
98	TLR3	Toll-like receptor 3 OS	0.36
99	EIF4H	Eukaryotic translation initiation factor 4H OS	0.34
100	AHSA1	Activator of 90 kDa heat shock protein ATPase homolog 1 OS	0.34
101	SNX9	Sorting nexin-9 OS	0.34
102	PABPN1	Polyadenylate-binding protein 2 (Fragment) OS	0.34
103	PPP1R7	Protein phosphatase 1 regulatory subunit 7 OS	0.33
104	TFAM	Transcription factor A, mitochondrial (Fragment) OS	0.32
105	POLR2A	DNA-directed RNA polymerase subunit OS	0.32
106	GNL3L	Guanine nucleotide-binding protein-like 3-like protein OS	0.31
107	PDXK	Pyridoxal kinase OS	0.30
108	PRRC2C	Protein PRRC2C OS	0.30
109	BOLA2	BolA-like protein 2 OS	0.28
110	WBSCR22	Probable 18S rRNA (guanine-N(7))-methyltransferase (Fragment) OS	0.27
111	PRPF4	U4/U6 small nuclear ribonucleoprotein Prp4 OS	0.27
112	CCDC40	Coiled-coil domain-containing protein 40 OS	0.26
113	DUSP9	Dual specificity protein phosphatase 9 OS	0.25
114	UTP18	U3 small nucleolar RNA-associated protein 18 homolog OS	0.25
115	CRK	Adapter molecule crk OS	0.24
116	NDUFB10	NADH dehydrogenase [ubiquinone] 1 beta subcomplex subunit 10 OS	0.23
117	FAM217A	Protein FAM217A OS	0.23
118	FUBP1	Far upstream element-binding protein 1 OS	0.23
119	ARL2	ADP-ribosylation factor-like protein 2 OS	0.22
120	PFAS	Phosphoribosylformylglycinamidine synthase OS	0.21
121	DDX54	ATP-dependent RNA helicase DDX54 OS	0.17
122	HEATR3	HEAT repeat-containing protein 3 OS	0.14
123	MAT2B	Methionine adenosyltransferase 2 subunit beta (Fragment) OS	0.14
124	PFDN1	Prefoldin subunit 1 OS	0.13
125	VPS50	Syndetin OS	0.12
126	CPNE8	Copine-8 OS	0.10
127	TPP2	Tripeptidyl-peptidase 2 OS	0.08
128	P2RX4	P2X purinoceptor 4 OS	0.06
129	ISOC1	Isochorismatase domain-containing protein 1 (Fragment) OS	0.06
130	MRPL57	Ribosomal protein 63, mitochondrial OS	0.04
131	DUS3L	tRNA-dihydrouridine(47) synthase [NAD(P)(+)]-like OS	0.02
132	BCL9L	B-cell CLL/lymphoma 9-like protein OS	0.02

Studies of glyoxalase 1-linked multidrug resistance reveal glycolysis-derived reactive metabolite, methylglyoxal, is a common contributor in cancer chemotherapy targeting the spliceosome.

Muhanad Alhujaily et al.

Table S9. Proteins of the mitochondrial membrane of HEK 293 cells increased by treatment with methylglyoxal.

No	Gene	Protein	Fold change
1	RPS5	Ribosomal protein S5	9.6
2	YLPM1	YLP motif-containing protein 1	9.4
3	CRTC3	CREB-regulated transcription coactivator 3	5.0
4	GRIN2D	Glutamate receptor ionotropic, NMDA 2D	4.4
5	RPS23	40S ribosomal protein S23	4.0
6	STATH	Statherin	3.2
7	GRM6	Metabotropic glutamate receptor 6	2.7
8	PHB	Prohibitin	2.5
9	UFSP1	Inactive Ufm1-specific protease 1	2.1

Studies of glyoxalase 1-linked multidrug resistance reveal glycolysis-derived reactive metabolite, methylglyoxal, is a common contributor in cancer chemotherapy targeting the spliceosome.

Muhanad Alhujaily et al.

Table S10. Proteins of the mitochondrial membrane of HEK 293 cells decreased by treatment with methylglyoxal.

No	Gene	Protein	Fold change
1	HSPD1	60 kDa heat shock protein, mitochondrial	0.69
2	TOPAZ1	Testis- and ovary-specific PAZ domain-containing protein 1	0.53
3	PRB1	Basic salivary proline-rich protein 1	0.51
4	TUBB	Tubulin beta chain	0.47
5	SLC25A6	ADP/ATP translocase 3	0.47
6	IL4	Interleukin 4 variant 2	0.46
7	RPS14	40S ribosomal protein S14	0.43
8	ATP5B	ATP synthase subunit beta, mitochondrial	0.39
9	APEH	Acylamino-acid-releasing enzyme	0.38
10	HSPA1B	Heat shock 70 kDa protein 1B	0.38
11	HIST1H1D	Histone H1.3	0.37
12	ATP5D	ATP synthase subunit delta, mitochondrial	0.37
13	RAVER2	Ribonucleoprotein PTB-binding 2	0.35
14	hCG_2039566	Histone H2A	0.35
15	COX5B	Cytochrome c oxidase subunit 5B, mitochondrial	0.32
16	YBX1	Nuclease-sensitive element-binding protein 1	0.32
17	HSPE1	10 kDa heat shock protein, mitochondrial	0.31
18	SDHB	Succinate dehydrogenase [ubiquinone] iron-sulfur subunit, mitochondrial	0.31
19	ATP5A1	ATP synthase subunit alpha, mitochondrial	0.31
20	EPB41L2	Band 4.1-like protein 2	0.31
21	STOML2	Stomatin-like protein 2, mitochondrial	0.31
22	SRSF3	Serine/arginine-rich splicing factor 3	0.30
23	MDH2	Malate dehydrogenase, mitochondrial	0.30
24	CAPNS1	Calpain small subunit 1	0.29
25	ACADVL	Very long-chain-specific acyl-CoA dehydrogenase, mitochondrial	0.28
26	ATP5O	ATP synthase subunit O, mitochondrial	0.28
27	ACTC1	Actin, alpha cardiac muscle 1	0.27
28	SHMT2	Serine hydroxymethyltransferase	0.27
29	GAPDH	Glyceraldehyde-3-phosphate dehydrogenase	0.26
30	UQCRB	Cytochrome b-c1 complex subunit 7	0.26
31	HSPA8	Heat shock cognate 71 kDa protein	0.26
32	S100A11	Protein S100-A11 (Calgizzarin)	0.25
33	LAMP2	Lysosome-associated membrane glycoprotein	0.25
34	PSIP1	PC4 and SFRS1-interacting protein	0.25
35	MATR3	Matrin-3	0.25
36	ATP2A2	Sarcoplasmic/endoplasmic reticulum calcium ATPase 2	0.25
37	PPIA	Peptidyl-prolyl cis-trans isomerase A	0.24
38	PHC3	Polyhomeotic-like protein 3	0.24
39	RPLP1	60S acidic ribosomal protein P1	0.23
40	HNRNPA1	Heterogeneous nuclear ribonucleoprotein A1	0.23
41	SLC25A11	Mitochondrial 2-oxoglutarate/malate carrier protein	0.23
42	EPHX1	Epoxide hydrolase 1	0.23
43	H2AFX	Histone H2AX	0.23

44	ACTB	Actin, cytoplasmic 1	0.22
45	SFXN1	Sideroflexin-1	0.22
46	ATP5J2-PTCD1	ATP synthase membrane subunit f	0.22
47	FAM162A	Protein FAM162A	0.21
48	ALYREF	THO complex subunit 4	0.21
49	FAM71E1	Protein FAM71E1	0.21
50	SEC61B	Protein transport protein Sec61 subunit beta	0.21
51	PPIB	Peptidyl-prolyl cis-trans isomerase B	0.21
52	BANF1	Barrier-to-autointegration factor	0.21
53	OCIAD1	OCIA domain-containing protein 1	0.20
54	COX7C	Cytochrome c oxidase subunit 7C, mitochondrial	0.20
55	ENO1	Alpha-enolase	0.20
56	IGF2BP1	Insulin-like growth factor 2 mRNA-binding protein 1	0.19
57	HSPA5	78 kDa glucose-regulated protein	0.19
58	IMMT	MICOS complex subunit MIC60	0.19
59	DDX5	Probable ATP-dependent RNA helicase DDX5	0.19
60	RCC1	Regulator of chromosome condensation	0.19
61	BCAP31	B-cell receptor-associated protein 31	0.19
62	PGRMC1	Membrane-associated progesterone receptor component 1	0.18
63	DNA2	DNA replication ATP-dependent helicase/nuclease DNA2	0.18
64	ENO2	Gamma-enolase	0.18
65	STAR	Steroidogenic acute regulatory protein, mitochondrial	0.18
66	RAB7A	Ras-related protein Rab-7a	0.18
67	CISD1	CDGSH iron-sulfur domain-containing protein 1	0.18
68	FUBP1	Far upstream element-binding protein 1	0.18
69	UQCR10	Cytochrome b-c1 complex subunit 9	0.18
70	SUB1	Activated RNA polymerase II transcriptional coactivator p15	0.17
71	SCAMP1	Secretory carrier-associated membrane protein	0.17
72	HADHA	Trifunctional enzyme subunit alpha, mitochondrial	0.16
73	LRRC59	Leucine-rich repeat-containing protein 59	0.16
74	EIF5A	Eukaryotic translation initiation factor 5A	0.16
75	CORO7-PAM16	Coronin	0.15
76	TIMM9	Mitochondrial import inner membrane translocase subunit Tim9	0.15
77	RPN1	Dolichyl-diphosphooligosaccharide--protein glycosyltransferase subunit 1	0.15
78	PRDX1	Peroxiredoxin-1	0.14
79	CYCS	Cytochrome c	0.14
80	HSP90AA1	Heat shock protein HSP 90-alpha	0.14
81	CCDC74A	Coiled-coil domain-containing protein 74A	0.14
82	PDIA3	Protein disulfide-isomerase A3	0.13
83	TAGLN2	Transgelin-2	0.13
84	COX5A	Cytochrome c oxidase subunit 5A, mitochondrial	0.13
85	NDUFA4	Cytochrome c oxidase subunit NDUFA4	0.13
86	ANXA2	Annexin	0.13
87	RBMX	RNA-binding motif protein, X chromosome	0.13
88	HNRNPH1	Heterogeneous nuclear ribonucleoprotein H	0.13
89	HMGB1	High mobility group protein B1	0.12
90	HADH	Hydroxyacyl-coenzyme A dehydrogenase, mitochondrial	0.12
91	NPM1	Nucleophosmin	0.11
92	PTBP1	Polypyrimidine tract-binding protein 1	0.11
93	NDUFA5	NADH dehydrogenase [ubiquinone] 1 alpha subcomplex subunit 5	0.11

94	ATP5H	ATP synthase subunit d, mitochondrial	0.11
95	PDCD5	Programmed cell death protein 5	0.10
96	SNRPC	U1 small nuclear ribonucleoprotein C	0.10
97	SCP2	Non-specific lipid-transfer protein	0.10
98	SSR3	Translocon-associated protein subunit gamma	0.10
99	MTDH	Protein LYRIC (Metadherin)	0.09
100	CALM2	Calmodulin	0.09
101	HNRNPC	Heterogeneous nuclear ribonucleoproteins C1/C2	0.09
102	NDUFB10	NADH dehydrogenase [ubiquinone] 1 beta subcomplex subunit 10	0.09
103	MRPS35	28S ribosomal protein S35, mitochondrial	0.08
104	TECR	Very-long-chain enoyl-CoA reductase	0.08
105	PDIA6	Protein disulfide-isomerase A6	0.08
106	SRSF1	Serine/arginine-rich splicing factor 1	0.06
107	LMAN2	Vesicular integral-membrane protein VIP36	0.06
108	DOCK8	Dedicator of cytokinesis protein 8	0.05
109	SYCP2	Synaptonemal complex protein 2	0.05
110	GOT2	Aspartate aminotransferase, mitochondrial	0.04
111	HS3ST3B1	Heparan sulfate glucosamine 3-O-sulfotransferase 3B1	0.04
112	HMGN2	Non-histone chromosomal protein HMG-17	0.02
113	UACA	Uveal autoantigen with coiled-coil domains and ankyrin repeats	0.02
114	ANXA5	Annexin A5	0.01
115	TIRAP	Toll/interleukin-1 receptor domain-containing adapter protein	0.01
116	TAF15	TATA-binding protein-associated factor 2N	0.00

Studies of glyoxalase 1-linked multidrug resistance reveal glycolysis-derived reactive metabolite, methylglyoxal, is a common contributor in cancer chemotherapy targeting the spliceosome.

Muhanad Alhujaily et al.

Table S11. Proteins of the mitochondrial membrane extract containing MG-H1 residues and changed in abundance by HEK293 cell treatment with methylglyoxal

No	Gene	Name of protein	Fold change
1	GRIN2D	Glutamate receptor ionotropic, NMDA 2D	4.44
2	GRM6	Metabotropic glutamate receptor 6	2.71
3	TOPAZ1	Testis- and ovary-specific PAZ domain-containing protein 1	0.53
4	ACADVL	Very long-chain-specific acyl-CoA dehydrogenase, mitochondrial	0.28
5	PHC3	Polyhomeotic-like protein 3	0.24
6	FAM71E1	Protein FAM71E1	0.21
7	ENO2	Gamma-enolase	0.18
8	STAR	Steroidogenic acute regulatory protein, mitochondrial	0.18
9	CCDC74A	Coiled-coil domain-containing protein 74A	0.14
10	DOCK8	Dedicator of cytokinesis protein 8	0.05
11	SYCP2	Synaptonemal complex protein 2	0.05
12	TIRAP	Toll/interleukin-1 receptor domain-containing adapter protein	0.01

Studies of glyoxalase 1-linked multidrug resistance reveal glycolysis-derived reactive metabolite, methylglyoxal, is a common contributor in cancer chemotherapy targeting the spliceosome.

Muhanad Alhujaily et al.

Table S12. Correlation of gene expression with Glo1 in the CCLE database of human tumor cell lines.

Correlation coefficients (Pearson)					
Positive correlations			negative correlations		
	Gene correlate	r		Gene correlate	r
1	PPIL1	0.568	1	CHAC1	-0.134
2	RPL7L1	0.566	2	PDXK	-0.134
3	PRIM2	0.531	3	NR1H3	-0.134
4	CDC5L	0.517	4	FBXW8	-0.135
5	CCT4	0.506	5	ZSWIM8	-0.135
6	CENPQ	0.489	6	TMEM143	-0.135
7	SF3B14	0.481	7	SLC39A3	-0.135
8	SNRPC	0.479	8	RP11.849H4.4	-0.135
9	CSE1L	0.463	9	DDIT4	-0.135
10	AC107081.5	0.458	10	SUSD1	-0.135
11	GPN3	0.454	11	FBXW4	-0.135
12	TAF11	0.449	12	KCNN4	-0.135
13	TTK	0.447	13	STAG3L5P.PV	-0.135
14	RPS10	0.445	14	CTSA	-0.135
15	RAD54B	0.445	15	MBD1	-0.135
16	LSM12	0.443	16	SDHAP1	-0.135
17	LSM2	0.438	17	CTC.512J14.5	-0.136
18	CDK1	0.436	18	MOGS	-0.136
19	RNF8	0.434	19	TOM1L2	-0.136
20	XPO1	0.431	20	WDR13	-0.136
21	RRP36	0.428	21	RP11.876N24.4	-0.136
22	RP11.355B11.2	0.426	22	UBALD2	-0.136
23	SRSF3	0.424	23	ITPKC	-0.136
24	MRPL19	0.423	24	RP11.425L10.1	-0.136
25	MTERFD1	0.418	25	ITPR3	-0.136
26	GMPS	0.418	26	CD44	-0.136
27	GMNN	0.417	27	KIF13B	-0.136
28	SRP9	0.414	28	PILRB	-0.136
29	LRRC40	0.414	29	DUSP28	-0.136
30	MAD2L1	0.413	30	RASSF7	-0.136
31	PAK1IP1	0.413	31	SEC24C	-0.137
32	ERCC6L	0.412	32	SLC3A2	-0.137
33	SUMO1	0.411	33	AGAP10	-0.137
34	HSP90AB1	0.410	34	PRDX5	-0.137
35	ORC3	0.410	35	ANAPC2	-0.137
36	OLA1	0.410	36	MCEE	-0.137
37	MIR3917	0.407	37	ANKRD10	-0.137
38	HDAC2	0.406	38	EXOC3	-0.137
39	LIN9	0.406	39	NCOR2	-0.137
40	NDC1	0.403	40	AKAP17A	-0.137

41	HNRNPC	0.401	41	MTND1P23	-0.137
42	PPP2R5D	0.400	42	LMAN2	-0.137
43	NUP54	0.400	43	CCDC146	-0.137
44	MRPL42	0.399	44	CYTH2	-0.137
45	NUDCD1	0.397	45	DCUN1D3	-0.137
46	UQCC2	0.396	46	SERINC2	-0.138
47	MRPS10	0.396	47	C21orf2	-0.138
48	RPF2	0.396	48	WBP1	-0.138
49	NUP155	0.395	49	LINC00999	-0.138
50	SPC25	0.393	50	DNAJC3	-0.138
51	BTF3L4	0.392	51	EIF2S3L	-0.138
52	NOL11	0.392	52	ZNF213	-0.138
53	SKA2	0.391	53	RABGGTA	-0.138
54	PTGES3	0.390	54	B4GALT1	-0.138
55	CCDC138	0.389	55	MT.RNR1	-0.138
56	MRPL47	0.389	56	ZNFX1	-0.138
57	CCT8	0.389	57	FADS3	-0.138
58	RRM1.AS1	0.388	58	MIR22HG	-0.138
59	NUP43	0.388	59	COL7A1	-0.138
60	MATR3	0.387	60	IRF2	-0.138
61	PCNP	0.386	61	ARHGAP26	-0.139
62	DFFA	0.386	62	FAM214B	-0.139
63	ZBTB9	0.386	63	ZNF408	-0.139
64	NUP35	0.386	64	TAGLN2	-0.139
65	RPP40	0.385	65	RP11.1246C19.	-0.139
66	SUV39H2	0.385	66	TMC4	-0.139
67	KHDRBS1	0.385	67	JAG1	-0.139
68	CCT7	0.384	68	SIL1	-0.139
69	GLMN	0.384	69	PER2	-0.139
70	MELK	0.384	70	CASP8	-0.139
71	SGOL2	0.384	71	WDR90	-0.139
72	DHFR	0.383	72	TMEM62	-0.139
73	UTP11L	0.382	73	AC005154.6	-0.139
74	TRMT10C	0.382	74	XBP1	-0.139
75	METTL2A	0.382	75	GLG1	-0.140
76	IPO11	0.382	76	ANKS3	-0.140
77	PSMC1	0.381	77	ZNF628	-0.140
78	KNSTRN	0.381	78	CCDC107	-0.140
79	CKS1B	0.381	79	CTSK	-0.140
80	USP1	0.380	80	CTD.2547E10.	-0.140
81	MAD2L1BP	0.380	81	RP11.296I10.6	-0.140
82	MRPL13	0.378	82	RP11.680G24.	-0.140
83	NPM1	0.378	83	AC084018.1	-0.140
84	CENPI	0.377	84	KIAA0895L	-0.140
85	RP11.342K6.1	0.376	85	ITGA3	-0.141
86	PSMD12	0.376	86	VPS13C	-0.141
87	VTA1	0.376	87	RNH1	-0.141
88	PCGF6	0.375	88	TECPR1	-0.141

89	KPNA2	0.375	89	ITGB1P1	-0.141
90	CDC25C	0.375	90	KRT8P33	-0.141
91	RP11.138C9.1	0.374	91	ARID5A	-0.141
92	HSPA4	0.374	92	KRCC1	-0.141
93	BYSL	0.374	93	CDKN1A	-0.141
94	XPO5	0.374	94	TRIM52	-0.142
95	NAE1	0.374	95	ADHFE1	-0.142
96	KIF11	0.373	96	ARHGAP23	-0.142
97	TCP1	0.373	97	MAP3K11	-0.142
98	COQ3	0.373	98	CDK6	-0.142
99	APOO	0.372	99	RP11.927P21.1	-0.142
100	KIAA0101	0.371	100	ATP13A1	-0.142
101	CHAF1B	0.370	101	RPS2P7	-0.142
102	XRCC5	0.370	102	MT.ND2	-0.142
103	HAT1	0.369	103	NME3	-0.142
104	PAICS	0.369	104	TMED9	-0.142
105	PNO1	0.369	105	ERVK13.1	-0.142
106	COIL	0.368	106	OGT	-0.142
107	GTF3C4	0.368	107	ADRBK1	-0.143
108	TPRKB	0.368	108	DHX38	-0.143
109	UTP18	0.368	109	S100A6	-0.143
110	DCLRE1B	0.367	110	RP11.263K19.[illegible]	-0.143
111	MLF1IP	0.367	111	AC005594.3	-0.143
112	MTBP	0.366	112	TTC17	-0.143
113	CEP78	0.366	113	CTU1	-0.143
114	NAA15	0.366	114	FER1L4	-0.143
115	CTD.2510F5.4	0.366	115	CTSC	-0.143
116	CDC27	0.365	116	RP11.347C12.[illegible]	-0.144
117	TAF9B	0.364	117	TMEM63A	-0.144
118	TIPIN	0.364	118	PROB1	-0.144
119	NUSAP1	0.364	119	FBXW7	-0.144
120	PARPBP	0.363	120	GP1BA	-0.144
121	MRPL14	0.363	121	RP11.458F8.4	-0.144
122	AHCTF1	0.361	122	RP4.669L17.10	-0.144
123	FKBPL	0.361	123	DNAJB2	-0.144
124	AMD1	0.361	124	ENKD1	-0.144
125	RP11.360L9.7	0.361	125	SEL1L3	-0.144
126	MMS22L	0.361	126	CUX1	-0.144
127	SNRNP40	0.360	127	SEC24D	-0.144
128	SSB	0.360	128	TMEM87B	-0.144
129	MRPL22	0.360	129	CMIP	-0.144
130	RFC5	0.360	130	RP11.446E9.1	-0.144
131	DEK	0.359	131	FAM193B	-0.144
132	FAM98B	0.359	132	RPL24P4	-0.144
133	RAD51AP1	0.359	133	RP11.498C9.2	-0.144
134	NCAPG	0.358	134	LBX2.AS1	-0.144
135	PSMB1	0.358	135	TAZ	-0.145
136	SNRPE	0.357	136	CHD2	-0.145

137	HNRNPK	0.357	137	CCDC85B	-0.145
138	VDAC2	0.357	138	FERMT3	-0.146
139	DYNC1LI1	0.357	139	MFSD3	-0.146
140	CCNB1	0.357	140	RPS6KA2	-0.146
141	VBP1	0.356	141	TYK2	-0.146
142	EEF1E1	0.356	142	GGT1	-0.146
143	HSPD1P1	0.355	143	TPST2	-0.146
144	RP11.303E16.8	0.355	144	IFI27	-0.146
145	SNRPD1	0.354	145	PSMC1P1	-0.146
146	PBK	0.354	146	MYCBP2	-0.146
147	GEMIN6	0.354	147	RPS7P10	-0.146
148	FBXO5	0.354	148	MPPE1	-0.146
149	CACYBP	0.353	149	NCOA3	-0.146
150	SLC25A17	0.353	150	CIC	-0.146
151	HBS1L	0.353	151	ZNF784	-0.147
152	RFC4	0.353	152	ELL2	-0.147
153	UBE2N	0.352	153	FAM156A	-0.147
154	RP11.15L13.4	0.352	154	PIGQ	-0.147
155	CDC7	0.352	155	ABHD17A	-0.147
156	NUF2	0.352	156	CATSPER2P1	-0.147
157	TOP2A	0.352	157	GPR89B	-0.147
158	EIF4E	0.351	158	TNK2	-0.147
159	IPO7	0.351	159	AC132872.1	-0.147
160	RP11.342K6.2	0.350	160	CYLD	-0.147
161	SMIM13	0.350	161	FICD	-0.147
162	PFDN6	0.350	162	LMF2	-0.147
163	RRM1	0.350	163	SPTBN1	-0.147
164	AL590762.1	0.350	164	AC007969.5	-0.147
165	GCFC2	0.349	165	AC000068.9	-0.148
166	PTTG1	0.349	166	PCNXL3	-0.148
167	ESCO2	0.349	167	LAMP1	-0.148
168	CCT5	0.349	168	RINL	-0.148
169	XRCC6	0.349	169	NLRP1	-0.148
170	NUP37	0.349	170	PLEC	-0.148
171	TUBD1	0.349	171	BMS1P5	-0.148
172	RPF1	0.348	172	MFSD10	-0.148
173	ZWILCH	0.348	173	GBAP1	-0.148
174	DSCC1	0.348	174	SRCAP	-0.148
175	KPNB1	0.348	175	RHOG	-0.148
176	MCM10	0.348	176	DGKQ	-0.148
177	ISCA1	0.348	177	AGAP7	-0.149
178	ST13	0.348	178	RP11.258F1.1	-0.149
179	GCSH	0.347	179	SMAD7	-0.149
180	CEP57L1	0.347	180	CLASRP	-0.149
181	STIL	0.347	181	VPS39	-0.149
182	PDCL3	0.347	182	POLM	-0.149
183	BRIX1	0.347	183	ELF1	-0.149
184	BCCIP	0.347	184	TMEM80	-0.149

185	OSGEPL1	0.346	185	AMY2B	-0.149
186	HSF2	0.346	186	TMEM258	-0.149
187	PPP1CC	0.346	187	RP11.143J12.2	-0.150
188	RMI1	0.346	188	VAMP8	-0.150
189	CSNK2B	0.346	189	TCF25	-0.150
190	NSL1	0.345	190	SPNS1	-0.150
191	CENPW	0.345	191	ANKZF1	-0.150
192	VDAC3	0.345	192	CAPG	-0.150
193	SERBP1	0.345	193	TBC1D3H	-0.150
194	RPAP3	0.345	194	AP001062.7	-0.151
195	CTB.43P18.1	0.344	195	AIM1	-0.151
196	UBE2T	0.344	196	VIMP	-0.151
197	UCHL5	0.343	197	GSTK1	-0.151
198	GINS4	0.343	198	ABHD4	-0.151
199	SET	0.343	199	TXNDC5	-0.151
200	LSM5	0.342	200	CCNL1	-0.151
201	AUNIP	0.342	201	AC009404.2	-0.151
202	PSMB3	0.342	202	TRAF3IP2	-0.151
203	LSM3	0.342	203	CBLB	-0.151
204	CCDC58	0.342	204	PGAM4	-0.151
205	ZW10	0.341	205	TSC22D4	-0.151
206	TRIP13	0.341	206	IDS	-0.151
207	TFAM	0.341	207	ATAD3C	-0.151
208	PSMD14	0.341	208	ARHGEF2	-0.152
209	POLR1C	0.341	209	OTUD5	-0.152
210	SGOL1	0.340	210	INE1	-0.152
211	STMN1	0.340	211	MAN2B1	-0.152
212	ATP5F1	0.340	212	JUND	-0.152
213	DLGAP5	0.340	213	ZFAND2A	-0.152
214	PPP2CA	0.340	214	RP11.361D15.2	-0.152
215	DDX20	0.340	215	ASMTL.AS1	-0.152
216	ILF2	0.339	216	HCLS1	-0.152
217	DNA2	0.339	217	TMEM129	-0.152
218	DEPDC1	0.339	218	HNRNPA1P10	-0.152
219	COPS2	0.339	219	RP11.274B21.1	-0.152
220	SUMO2	0.339	220	NSUN5P1	-0.152
221	GEMIN5	0.339	221	NOMO3	-0.152
222	CCT2	0.339	222	GUK1	-0.152
223	POLE2	0.339	223	RDH14	-0.152
224	KIFC1	0.338	224	SQSTM1	-0.152
225	RP5.1113E3.3	0.338	225	TPP1	-0.153
226	DHX9	0.337	226	ACSF3	-0.153
227	NUP107	0.337	227	SLC25A45	-0.153
228	MCM3	0.337	228	ANXA2P2	-0.153
229	KIF15	0.337	229	CLEC16A	-0.153
230	MRPL50	0.337	230	RP11.39C10.1	-0.153
231	RP11.474G23.2	0.337	231	WWP2	-0.153
232	RACGAP1	0.336	232	ARPC1B	-0.153

233	CCT3	0.336	233	ARPC2	-0.153
234	HPRT1	0.336	234	PSKH1	-0.153
235	CBX1	0.336	235	SLC10A3	-0.153
236	ATAD2	0.336	236	AP003068.9	-0.153
237	ORC4	0.336	237	RRN3P1	-0.154
238	TMEM14B	0.336	238	RNF31	-0.154
239	LTV1	0.335	239	BMS1P1	-0.154
240	TIMM8A	0.335	240	RP3.368A4.6	-0.154
241	HNRNPA3	0.335	241	NFE2L1	-0.154
242	MIS18A	0.335	242	FTX	-0.154
243	RFC2	0.335	243	TNRC18	-0.154
244	KIF4A	0.335	244	CTD.3126B10.	-0.154
245	DTL	0.334	245	RP11.347C12.1	-0.154
246	RPA2	0.334	246	TBC1D2	-0.154
247	C11orf82	0.334	247	IFI27L2	-0.155
248	SSX2IP	0.334	248	PDF	-0.155
249	ARMC1	0.334	249	SLC12A9	-0.155
250	RFC3	0.334	250	HLA.F	-0.155
251	GNAI3	0.334	251	NUCB1.AS1	-0.155
252	DONSON	0.334	252	LAT2	-0.155
253	SLC25A33	0.333	253	CCDC78	-0.155
254	ELAVL1	0.333	254	CTBP1	-0.155
255	ASNSD1	0.333	255	HLA.C	-0.156
256	TIMM23B	0.333	256	RP11.493K19.3	-0.156
257	DARS2	0.333	257	RP11.179G5.1	-0.156
258	CCNB2	0.333	258	AC021224.1	-0.156
259	MDH1	0.333	259	NARFL	-0.156
260	PSMG1	0.333	260	VDAC1P1	-0.156
261	SMC3	0.333	261	RP11.20I23.8	-0.156
262	DBF4	0.333	262	NPIPA5	-0.156
263	UBA2	0.332	263	GOLGA6L5	-0.156
264	DSN1	0.332	264	HLA.DMA	-0.156
265	POLA1	0.331	265	RP11.395L14.1	-0.157
266	DENR	0.331	266	MYH9	-0.157
267	NEDD1	0.330	267	NUCB1	-0.157
268	EIF1AX	0.330	268	DPP7	-0.157
269	SKA3	0.330	269	FUK	-0.157
270	UNG	0.330	270	IER2	-0.157
271	DCAF13	0.329	271	WDR81	-0.157
272	AASDHPPT	0.329	272	STX5	-0.157
273	HSPE1	0.329	273	EML2	-0.157
274	MCMBP	0.328	274	ZNF319	-0.157
275	ZCRB1	0.328	275	PBXIP1	-0.157
276	WDYHV1	0.328	276	HNRNPA1P7	-0.157
277	GART	0.328	277	CCM2	-0.157
278	SNRPB	0.328	278	FAM203B	-0.157
279	ANKRD32	0.327	279	AL162151.3	-0.158
280	UBXN2A	0.327	280	ELMO3	-0.158

281	HNRNPA1	0.327	281	RPL5P4	-0.158
282	TMEM177	0.327	282	CTSD	-0.158
283	ETF1	0.327	283	ACBD4	-0.158
284	MED20	0.327	284	UBQLN4P1	-0.158
285	SNRPG	0.327	285	OVGP1	-0.159
286	NUP153	0.327	286	TBC1D10A	-0.159
287	HAUS6	0.326	287	TRABD	-0.159
288	GINS1	0.326	288	MXD4	-0.159
289	FANCB	0.325	289	SDHAP2	-0.159
290	TAF1B	0.325	290	RRBP1	-0.160
291	CENPL	0.325	291	FKBP2	-0.160
292	ORC2	0.325	292	RP5.1142A6.9	-0.160
293	WDR75	0.325	293	TBRG1	-0.160
294	MRTO4	0.324	294	STUB1	-0.160
295	RBMXL1	0.324	295	DDA1	-0.160
296	MCUR1	0.324	296	MOB2	-0.160
297	MAGOHB	0.324	297	GADD45A	-0.160
298	PPAT	0.324	298	SLC7A7	-0.160
299	CCT6A	0.324	299	MGRN1	-0.160
300	UBE2V2	0.324	300	ZMIZ2	-0.160
301	MRPS22	0.323	301	RP11.112J3.15	-0.160
302	CTD.2256P15.4	0.323	302	ANKH	-0.160
303	SKP2	0.323	303	BANF1P3	-0.161
304	PHF13	0.323	304	AFF1	-0.161
305	TMPO	0.323	305	TMEM243	-0.161
306	MRPL39	0.323	306	CAPN1	-0.161
307	ZMYM1	0.323	307	ANXA11	-0.161
308	ABCE1	0.322	308	SLC8B1	-0.161
309	CCZ1B	0.322	309	MANBA	-0.161
310	MRPL3	0.322	310	MPRIPP1	-0.161
311	FAM104B	0.322	311	AMT	-0.161
312	TUBB	0.322	312	RP11.572P18.1	-0.162
313	CDKN2AIPNL	0.321	313	API5P1	-0.162
314	BAG4	0.321	314	RP5.1050D4.2	-0.162
315	CCNE2	0.321	315	CAPS	-0.162
316	SPAG5	0.321	316	LA16c.349E10.	-0.162
317	ERAL1	0.321	317	RP11.252A24.2	-0.162
318	CMSS1	0.321	318	F8A2	-0.162
319	TOMM22	0.320	319	RP11.146F11.1	-0.162
320	NUFIP1	0.320	320	MNT	-0.162
321	NEK2	0.320	321	NAGLU	-0.162
322	RP11.144G7.2	0.320	322	EEF1A1P13	-0.162
323	NDC80	0.319	323	UBE2SP2	-0.162
324	NDUFB4	0.319	324	TNXB	-0.162
325	EIF2S1	0.319	325	RP11.958N24.2	-0.162
326	SKP1	0.319	326	SLC35E1	-0.162
327	MAGOH	0.319	327	ANKRD13D	-0.163
328	PSMA4	0.319	328	ACADVL	-0.163

329	WDR46	0.318	329	DYX1C1.CCP(	-0.163
330	TIMM23	0.318	330	BIRC3	-0.163
331	RRM2	0.318	331	HIVEP2	-0.163
332	DESI2	0.318	332	FASTK	-0.163
333	MRPS23	0.318	333	HSF4	-0.163
334	FARSB	0.318	334	CENPT	-0.163
335	EXO1	0.318	335	RP4.800G7.2	-0.163
336	MRPS15	0.317	336	NPIPB5	-0.163
337	MRPL51	0.317	337	TBC1D3G	-0.163
338	HAUS1	0.316	338	LAT	-0.163
339	PRPF4	0.316	339	ISCA1P1	-0.163
340	C4orf46	0.316	340	HM13	-0.163
341	KIAA1524	0.316	341	SLC52A2	-0.163
342	USP39	0.316	342	PISD	-0.164
343	MASTL	0.316	343	HMGN2P5	-0.164
344	TPX2	0.316	344	POLR2J4	-0.164
345	C2orf69	0.316	345	ALPK1	-0.164
346	CDC6	0.316	346	C17orf59	-0.164
347	PRIM1	0.316	347	P4HB	-0.164
348	MSH2	0.315	348	SRPR	-0.164
349	KIF23	0.315	349	EIF5AL1	-0.164
350	BRIP1	0.315	350	RPS3AP25	-0.165
351	ZNF146	0.315	351	RP11.163E9.2	-0.165
352	SAYSD1	0.315	352	RP11.490H24.5	-0.165
353	RPL21	0.314	353	RGPD1	-0.165
354	BCAS2	0.314	354	RPL4P4	-0.165
355	CETN3	0.314	355	ELL	-0.165
356	CDC23	0.314	356	ZFAND2B	-0.165
357	PPP3R1	0.314	357	AC073869.1	-0.165
358	HMGB1	0.314	358	RTN3P1	-0.165
359	ACTL6A	0.313	359	HLA.A	-0.165
360	SF3A3	0.313	360	AC104134.2	-0.165
361	TEX10	0.313	361	MFSD6	-0.166
362	MRPS9	0.313	362	RPL39P3	-0.166
363	KIAA1143	0.313	363	MAP2K3	-0.166
364	ZNF644	0.313	364	NPIPB4	-0.166
365	RBM27	0.313	365	FAM157C	-0.166
366	AC008132.12	0.312	366	TAF10	-0.167
367	CENPH	0.312	367	RP11.497H16.5	-0.167
368	AURKA	0.312	368	ADCY7	-0.167
369	SNX4	0.312	369	CTC.338M12.7	-0.167
370	RBMX	0.312	370	STAG3L2	-0.167
371	HSPD1	0.312	371	MT.CYB	-0.167
372	RPL6	0.312	372	RP11.164J13.1	-0.167
373	RAD18	0.311	373	TTLL3	-0.167
374	HSP90AA1	0.311	374	B4GALT7	-0.167
375	GTF2H2	0.311	375	ACRC	-0.167
376	E2F6	0.311	376	RPS7P1	-0.167

377	H2AFZ	0.311	377	AC138969.4	-0.167
378	NOC3L	0.311	378	NEK8	-0.167
379	METTL6	0.310	379	NMRK1	-0.167
380	TBCE	0.310	380	RP11.556K13.1	-0.168
381	CCDC77	0.310	381	RP4.673M15.1	-0.168
382	C2orf44	0.309	382	RNPS1P1	-0.168
383	FXR1	0.309	383	PKD1P5	-0.168
384	CCNA2	0.309	384	CYB561D1	-0.168
385	CNOT6	0.309	385	TSR3	-0.168
386	WDR5	0.309	386	LRCH4	-0.168
387	ERH	0.309	387	CCDC9	-0.168
388	RNF2	0.308	388	SLC12A6	-0.168
389	KIF20A	0.308	389	RHOT2	-0.168
390	RANBP1	0.308	390	EML3	-0.168
391	RPA3	0.308	391	TPRA1	-0.169
392	NUP85	0.308	392	CCPG1	-0.169
393	ACTR6	0.308	393	CTD.3065B20.	-0.169
394	C5orf34	0.307	394	RP11.567G24.1	-0.169
395	GAR1	0.307	395	ZNF785	-0.169
396	MRPL37	0.307	396	C4B	-0.169
397	KIF2C	0.307	397	RP11.758P17.3	-0.169
398	RAD51C	0.306	398	ANKRD61	-0.169
399	BUB1B	0.306	399	CSAD	-0.169
400	CASP8AP2	0.306	400	BCL3	-0.169
401	SASS6	0.306	401	SLX1B	-0.169
402	COX7A2	0.306	402	RAB40C	-0.169
403	BLM	0.306	403	VPS51	-0.169
404	NDUFB6	0.306	404	ZFYVE27	-0.169
405	VMA21	0.306	405	TLK2P1	-0.170
406	PSRC1	0.305	406	SLC22A20	-0.170
407	NLN	0.305	407	ANP32AP1	-0.170
408	NIF3L1	0.305	408	CTD.3193O13.	-0.170
409	CBX3	0.305	409	RNF213	-0.170
410	CDCA2	0.305	410	RP11.91A18.4	-0.170
411	DRG1	0.305	411	AC018804.6	-0.171
412	H3F3A	0.305	412	ST3GAL1	-0.171
413	RP11.212P7.3	0.305	413	UVSSA	-0.171
414	BZW1	0.305	414	R3HDM4	-0.171
415	RPS26	0.305	415	TUBA4A	-0.171
416	FBXO45	0.304	416	LINC00894	-0.171
417	CEBPZ	0.304	417	CLSTN3	-0.171
418	MRPL18	0.304	418	CLTB	-0.171
419	POC1A	0.304	419	GPR108	-0.171
420	NASP	0.304	420	SLC39A13	-0.171
421	RPL39	0.304	421	RP11.274B21.4	-0.171
422	CKS2	0.304	422	STAT5A	-0.171
423	KLHL23	0.303	423	SEMA4B	-0.172
424	C1orf112	0.303	424	PARP3	-0.172

425	GTF2H3	0.303	425	ARRDC1	-0.172
426	NUS1	0.303	426	SLC12A4	-0.172
427	MND1	0.303	427	DBNL	-0.172
428	PAPOLA	0.303	428	PPP1R12B	-0.172
429	PRDX3	0.303	429	HLA.E	-0.172
430	STIP1	0.302	430	LCAT	-0.172
431	MCM6	0.302	431	AC093818.1	-0.172
432	RNGTT	0.302	432	RP11.274B21.2	-0.172
433	NUP205	0.302	433	INO80B	-0.172
434	DEPDC4	0.302	434	AC004057.1	-0.172
435	NOL10	0.302	435	AC068580.6	-0.172
436	ATG4C	0.302	436	CTC.471F3.4	-0.172
437	EFTUD2	0.302	437	GOLGA6L10.1	-0.173
438	DEPDC1B	0.301	438	EHD1	-0.173
439	PAIP1	0.301	439	AC018738.2	-0.173
440	KLHDC3	0.301	440	CLCN7	-0.173
441	DNAJC8	0.301	441	TRIM41	-0.173
442	KIF18A	0.301	442	AC083873.4	-0.173
443	METAP2	0.301	443	C1S	-0.173
444	MKI67IP	0.301	444	MAPK13	-0.174
445	SRPK1	0.301	445	NPIPA1	-0.174
446	PFDN4	0.300	446	MAVS	-0.174
447	KATNBL1	0.300	447	ATG2A	-0.174
448	HNRNPA2B1	0.300	448	RP11.783K16.1	-0.174
449	LRPPRC	0.300	449	RP11.244H3.1	-0.174
450	CSNK2A1	0.300	450	GOLGA3	-0.174
451	LRRC58	0.300	451	SNRPEP4	-0.174
452	ZNF451	0.300	452	AC027612.1	-0.174
453	USP33	0.299	453	ZC3H11B	-0.174
454	ZWINT	0.299	454	CFLAR	-0.174
455	PRRC2A	0.299	455	CCDC159	-0.175
456	RIOK1	0.299	456	TPCN1	-0.175
457	TBC1D31	0.299	457	AC015849.19	-0.175
458	CENPF	0.299	458	CCDC88B	-0.175
459	BCLAF1	0.299	459	CCDC130	-0.175
460	OIP5	0.299	460	TRIM73	-0.175
461	CENPK	0.299	461	ADCK5	-0.175
462	XXbac.BPG252	0.299	462	STOML1	-0.175
463	EXOSC2	0.299	463	PIGG	-0.175
464	MZT1	0.298	464	SCAMP2	-0.175
465	TTC26	0.298	465	SLC1A5	-0.175
466	ATP5O	0.298	466	GHDC	-0.176
467	ITGB3BP	0.298	467	SZT2.AS1	-0.176
468	PHF6	0.298	468	CD27.AS1	-0.176
469	NCAPH	0.298	469	PDPK2	-0.176
470	HELLS	0.297	470	SEC61A1	-0.176
471	UQCR11	0.297	471	LRRC37A2	-0.176
472	STXBP4	0.297	472	VMAC	-0.176

473	LMNB1	0.297	473	RP11.473M20.'	-0.176
474	GBAS	0.297	474	GOLGA8I	-0.176
475	FASTKD3	0.296	475	AC138035.2	-0.176
476	CDC25A	0.296	476	ZZEF1	-0.176
477	ACAT2	0.296	477	MR1	-0.176
478	ATG5	0.296	478	MPRIP	-0.176
479	WDR3	0.296	479	ABCA7	-0.177
480	SYNCRIP	0.296	480	CCDC57	-0.177
481	NELFE	0.296	481	RP11.408P14.1	-0.177
482	SEH1L	0.296	482	KIAA0556	-0.177
483	E2F3	0.295	483	TSC2	-0.177
484	WDHD1	0.295	484	MGLL	-0.177
485	FKBP3	0.295	485	ARFGAP1	-0.177
486	TARDBP	0.295	486	C1orf63	-0.177
487	SMS	0.295	487	POU2F2	-0.177
488	EIF3E	0.295	488	FAM73B	-0.177
489	RP11.51O6.1	0.294	489	KLF13	-0.177
490	PUS7	0.294	490	SPG7	-0.177
491	INTS8	0.294	491	MKLN1.AS1	-0.177
492	NCBP1	0.294	492	NONOP2	-0.178
493	TRMT61B	0.294	493	CAPN15	-0.178
494	PSMC6	0.294	494	LMO7	-0.178
495	CENPE	0.294	495	LTB4R	-0.178
496	WDR76	0.294	496	SNX29	-0.178
497	HNRNPR	0.294	497	ZCCHC6	-0.178
498	RP11.552M11.4	0.294	498	CIITA	-0.178
499	PCMT1	0.294	499	TRIM21	-0.178
500	KIF20B	0.294	500	GLUD1P3	-0.178
501	HRSP12	0.294	501	MST1	-0.179
502	RP3.337H4.10	0.294	502	C11orf35	-0.179
503	WDR77	0.294	503	LINC01001	-0.179
504	RPS18	0.293	504	EIF2AK3	-0.179
505	ATP5J	0.293	505	NUDT18	-0.179
506	CENPA	0.293	506	CAPN3	-0.179
507	SMC2	0.293	507	ESRRA	-0.179
508	MRPL35	0.293	508	SIDT2	-0.179
509	ORC6	0.292	509	PPIAP22	-0.179
510	AIDA	0.292	510	GLUD2	-0.179
511	RP11.156E6.1	0.292	511	RP11.228B15.4	-0.179
512	CAND1	0.292	512	AP1G2	-0.180
513	CKAP2	0.292	513	C4A	-0.180
514	MRPL15	0.291	514	YWHAZP3	-0.180
515	ARHGAP11A	0.291	515	AC098614.2	-0.180
516	EXOSC9	0.291	516	HAGHL	-0.180
517	TRA2B	0.291	517	RP11.66N24.4	-0.180
518	SNHG16	0.291	518	MST1R	-0.180
519	KIAA1429	0.291	519	RP4.717I23.3	-0.180
520	C2orf76	0.291	520	AC135048.13	-0.180

521	DNAJA1	0.291	521	RP3.437C15.1	-0.180
522	KDM1A	0.291	522	C20orf24	-0.181
523	EXOSC3	0.290	523	MC1R	-0.181
524	RWDD4	0.290	524	CREBRF	-0.181
525	HSPB11	0.290	525	KHNYN	-0.181
526	PPID	0.290	526	HDAC7	-0.182
527	NUPL1	0.290	527	LINC00174	-0.182
528	ECT2	0.290	528	CCNDBP1	-0.182
529	RUVBL1	0.290	529	PIEZO1	-0.182
530	MORF4L1	0.290	530	WASH4P	-0.182
531	RHNO1	0.289	531	DNASE1L1	-0.182
532	VRK1	0.289	532	PKD1P6	-0.182
533	CDC42	0.289	533	CHKB	-0.182
534	PIP5K1A	0.289	534	RP11.361L15.4	-0.182
535	PLK4	0.289	535	DENND3	-0.183
536	RAE1	0.289	536	MXD1	-0.183
537	CCNC	0.289	537	AC004878.3	-0.183
538	CNOT10	0.289	538	MAN2B2	-0.183
539	PPP4R2	0.288	539	ORAI2	-0.183
540	SNRPF	0.288	540	RP11.512F24.1	-0.183
541	SELRC1	0.288	541	SBF1	-0.183
542	MEA1	0.288	542	PMS2P2	-0.184
543	NKIRAS1	0.288	543	MMP19	-0.184
544	BUB1	0.288	544	CTD.3185P2.1	-0.184
545	LACE1	0.288	545	ARHGAP27	-0.184
546	ARMC8	0.287	546	SMARCA2	-0.184
547	FAM161A	0.287	547	FAM160A2	-0.184
548	UBE2V1	0.287	548	APOL2	-0.185
549	PRIMPOL	0.287	549	DDRGK1	-0.185
550	HAUS2	0.287	550	PRELID1P1	-0.185
551	ECD	0.286	551	TMEM214	-0.185
552	HMGXB4	0.286	552	GS1.44D20.1	-0.185
553	PPP6C	0.286	553	ANKRD11	-0.186
554	TOMM20	0.286	554	MYD88	-0.186
555	TEFM	0.286	555	TFEB	-0.186
556	PHF5A	0.286	556	ATF4P3	-0.186
557	WRNIP1	0.286	557	RP3.508I15.14	-0.186
558	HACE1	0.286	558	NPIPB3	-0.186
559	ORC1	0.286	559	EPS8L2	-0.186
560	CENPO	0.286	560	AC010468.1	-0.186
561	LYRM2	0.286	561	CHPF	-0.186
562	RP11.118M9.3	0.286	562	RP11.274B21.3	-0.186
563	YTHDF2	0.286	563	IRF3	-0.186
564	KATNA1	0.285	564	SPDYE2B	-0.187
565	NDUFB5	0.285	565	SLC16A3	-0.187
566	TSN	0.285	566	GLRX	-0.187
567	PPIL4	0.284	567	GRN	-0.187
568	SLIRP	0.284	568	PKD1P1	-0.187

569	DHX57	0.284	569	NPM1P27	-0.187
570	PRPF40A	0.284	570	PPARA	-0.187
571	RP11.10N23.4	0.284	571	C1DP1	-0.187
572	GMFB	0.284	572	AC002310.7	-0.188
573	ERI3	0.284	573	ZNF598	-0.188
574	TRIM37	0.283	574	GS1.124K5.11	-0.188
575	EMG1	0.283	575	COMTD1	-0.188
576	PTPLAD1	0.283	576	NUDT22	-0.188
577	PSMA1	0.283	577	RP11.894P9.1	-0.188
578	RPS3A	0.283	578	CNPPD1	-0.189
579	COPS4	0.283	579	ORAI3	-0.189
580	TIPRL	0.283	580	PPP1R15A	-0.189
581	MAP6D1	0.283	581	ERAP2	-0.189
582	LZIC	0.283	582	RAB4B	-0.189
583	L2HGDH	0.283	583	STK10	-0.189
584	BANF1	0.283	584	ARFRP1	-0.190
585	DIMT1	0.283	585	LENG8.AS1	-0.190
586	KIF14	0.283	586	RP13.996F3.5	-0.190
587	ZNF473	0.283	587	AKAP13	-0.190
588	SKIV2L2	0.282	588	BET1L	-0.190
589	ZFP1	0.282	589	GMIP	-0.190
590	DCTN4	0.282	590	TMEM127	-0.190
591	CDK4	0.282	591	EIF1AXP1	-0.191
592	IPO9	0.282	592	FTLP3	-0.191
593	TMEM183A	0.282	593	TMEM8A	-0.191
594	GEMIN2	0.282	594	AC005822.1	-0.191
595	ZNF143	0.282	595	NABP1	-0.191
596	CNOT7	0.282	596	FAM214A	-0.191
597	NAA50	0.282	597	TGFBR2	-0.191
598	RNF5	0.281	598	ARAP1.AS1	-0.191
599	RNASEH2A	0.281	599	SDF4	-0.191
600	CHCHD4	0.281	600	AC011737.2	-0.191
601	AC104655.2	0.281	601	RPL4P5	-0.191
602	YWHAE	0.281	602	TRIM66	-0.191
603	AC000068.10	0.280	603	RP11.641D5.1	-0.191
604	GXYLT1	0.280	604	CD74	-0.192
605	ZMYM4	0.280	605	WDR24	-0.192
606	PRR11	0.280	606	TPGS1	-0.192
607	LYRM4	0.280	607	GET4	-0.192
608	MTFR2	0.280	608	HLA.B	-0.192
609	GTF2H2B	0.280	609	RP11.84G21.1	-0.192
610	RP11.677I18.3	0.280	610	RHBDL1	-0.192
611	AC007390.5	0.280	611	SQRDL	-0.192
612	FKBP4	0.280	612	ENTHD2	-0.192
613	RPL23A	0.280	613	ABHD17AP1	-0.192
614	YEATS4	0.280	614	RELB	-0.192
615	LARP4	0.279	615	PCNXL2	-0.193
616	PSMD11	0.279	616	CBX4	-0.193

617	ORC5	0.279	617	RP5.1050D4.3	-0.193
618	POLR3F	0.279	618	EIF2S2P4	-0.193
619	NUDT21	0.279	619	CAMTA2	-0.193
620	NUP93	0.279	620	ACAP3	-0.194
621	PSMB4	0.279	621	CTC.250I14.6	-0.194
622	MED17	0.279	622	MT.CO2	-0.194
623	NUP133	0.279	623	RP11.489E7.4	-0.194
624	LRR1	0.278	624	TRAF1	-0.194
625	DPM1	0.278	625	KLC1	-0.194
626	ZCCHC17	0.278	626	SMG1P1	-0.194
627	CDC26	0.278	627	DNHD1	-0.195
628	CCDC117	0.278	628	RP11.20O24.4	-0.195
629	ZYG11B	0.278	629	STX4	-0.195
630	CPSF3	0.278	630	TNFAIP3	-0.195
631	GNL3	0.278	631	DHRS1	-0.195
632	DDX50	0.278	632	LINC00893	-0.196
633	DBR1	0.278	633	HEXDC	-0.196
634	YWHAQ	0.278	634	TBC1D17	-0.196
635	PRC1	0.278	635	LINC00672	-0.196
636	SAAL1	0.278	636	NOL3	-0.196
637	MORN2	0.278	637	CDK10	-0.197
638	MEX3A	0.278	638	SLX1B.SULT1	-0.197
639	ENY2	0.278	639	SERINC3	-0.197
640	CCDC59	0.278	640	LA16c.390E6.5	-0.197
641	ZNHIT3	0.278	641	DTX2	-0.197
642	RP11.312J18.5	0.278	642	CES2	-0.197
643	CDKN3	0.278	643	PEX16	-0.198
644	SRSF7	0.277	644	FBRS	-0.198
645	PWP1	0.277	645	RP11.3P17.3	-0.198
646	WARS2	0.277	646	SULT1A3	-0.198
647	RPS7	0.277	647	RABAC1	-0.198
648	TBCA	0.276	648	GSDMB	-0.198
649	FANCI	0.276	649	CTB.31O20.4	-0.198
650	TAF5	0.276	650	ISG20	-0.199
651	USP14	0.276	651	RPL14P1	-0.199
652	RARS2	0.276	652	MPG	-0.199
653	PAIP2	0.276	653	ARHGEF1	-0.199
654	GTF2H5	0.276	654	RP11.587D21.1	-0.199
655	ADSL	0.276	655	TMEM198B	-0.200
656	G3BP1	0.276	656	CSNK2A3	-0.200
657	CLSPN	0.276	657	RRN3P3	-0.200
658	WBP11	0.276	658	ENSAP2	-0.200
659	RAD54L	0.276	659	ADAM8	-0.200
660	RP11.360L9.4	0.275	660	NR1H2	-0.201
661	POLB	0.275	661	MT.CO1	-0.201
662	THUMPD3	0.275	662	GAK	-0.201
663	NDUFC1	0.275	663	MIR1282	-0.201
664	CDCA7	0.275	664	RPL41P1	-0.201

665	RP4.785G19.5	0.275	665	CPEB2	-0.202
666	PSMB2	0.275	666	PMS2P3	-0.202
667	HNRNPAB	0.275	667	CTGLF11P	-0.202
668	CAPN7	0.275	668	EIF4BP6	-0.202
669	LARS	0.275	669	HNRNPA1P48	-0.203
670	SMC4	0.274	670	OGFOD2	-0.203
671	SSBP1	0.274	671	SH2B1	-0.203
672	NFU1	0.274	672	RP11.203F10.6	-0.203
673	SPRTN	0.274	673	PLK3	-0.203
674	CDC40	0.274	674	RP11.632C17_	-0.204
675	STRAP	0.274	675	RPL7AP30	-0.204
676	CDC45	0.274	676	RP11.47A8.5	-0.204
677	CAMTA1	0.274	677	RP11.108K14.4	-0.204
678	POLR2D	0.274	678	YBX1P1	-0.204
679	CHCHD1	0.274	679	SIRT7	-0.204
680	CDCA3	0.274	680	LPXN	-0.205
681	SMARCC1	0.274	681	NIPAL3	-0.205
682	MTCH2	0.274	682	ANKMY1	-0.205
683	SAE1	0.274	683	TNIP1	-0.205
684	RAD21	0.274	684	PDLIM1	-0.205
685	UTP15	0.274	685	PHYKPL	-0.205
686	ERCC8	0.274	686	SLX1A.SULT1	-0.206
687	DENND6A	0.273	687	AC138783.12	-0.206
688	MRPL11	0.273	688	FTH1P10	-0.206
689	THOC1	0.273	689	SPRYD3	-0.206
690	FANCC	0.273	690	RP11.574K11.2	-0.206
691	MRPS28	0.273	691	UBALD1	-0.206
692	RPP30	0.272	692	MINK1	-0.206
693	RP4.635E18.6	0.272	693	CTD.2139B15.[illegible]	-0.206
694	MRPS27	0.272	694	RP11.395P17.3	-0.206
695	HSPA14	0.272	695	APOL6	-0.206
696	ERI1	0.272	696	STAT6	-0.207
697	RP11.269F19.2	0.272	697	SERBP1P5	-0.207
698	GNG5	0.272	698	CDC42P6	-0.207
699	CUL3	0.272	699	TXNIP	-0.207
700	ZNF367	0.272	700	TCP11L2	-0.207
701	KBTBD6	0.272	701	BLCAP	-0.208
702	AHCY	0.271	702	VPS9D1	-0.208
703	NDUFA8	0.271	703	TICAM1	-0.208
704	XPO7	0.271	704	CYP3A5	-0.208
705	BIRC5	0.271	705	C19orf10	-0.208
706	PCNA	0.271	706	ST13P4	-0.209
707	DDX18	0.271	707	RAD51.AS1	-0.209
708	BOLA3	0.270	708	RP5.857K21.7	-0.209
709	GTF2H4	0.270	709	JUNB	-0.210
710	RAN	0.270	710	RP11.324H6.5	-0.210
711	RNF138	0.270	711	ALDH3B1	-0.210
712	SNX3	0.270	712	ZNF276	-0.210

713	RPL24	0.270	713	RPS7P11	-0.211
714	NUDT15	0.270	714	FAUP1	-0.211
715	RAD1	0.270	715	TBC1D22A	-0.211
716	PSMA5	0.270	716	TPI1P1	-0.211
717	NCAPG2	0.269	717	MAN2C1	-0.211
718	C5orf22	0.269	718	RPL21P28	-0.212
719	C17orf58	0.269	719	AMPD3	-0.212
720	POLR1E	0.269	720	RP11.9L18.2	-0.212
721	NEK4	0.269	721	NPIPA2	-0.212
722	CTB.131K11.1	0.269	722	CYB561A3	-0.212
723	PDRG1	0.269	723	GTF2IRD2P1	-0.212
724	SGOL1.AS1	0.269	724	RPL12P4	-0.212
725	TUBA1B	0.269	725	MT.ND5	-0.212
726	THAP1	0.269	726	DYNC1I2P1	-0.212
727	FANCE	0.269	727	AC016734.2	-0.213
728	PSIP1	0.268	728	ARHGAP1	-0.213
729	FAM136A	0.268	729	PSD4	-0.214
730	ANKRD18EP	0.268	730	ERN1	-0.214
731	XXbac.BPG300	0.268	731	CYBA	-0.214
732	RIF1	0.268	732	TRIM8	-0.214
733	MCM8	0.268	733	FBXW5	-0.214
734	MRPS18B	0.268	734	RP11.958N24.1	-0.215
735	CCDC121	0.268	735	ZDHHC24	-0.216
736	BRMS1L	0.268	736	RP11.819M15.[illegible]	-0.216
737	NDUFA6	0.268	737	RP4.592A1.2	-0.216
738	SMN2	0.268	738	RP11.23N2.4	-0.217
739	SPAST	0.267	739	RP11.1212A22	-0.217
740	CSDE1	0.267	740	SEL1L	-0.217
741	QTRTD1	0.267	741	MYO18A	-0.217
742	ZC3HC1	0.267	742	SZT2	-0.218
743	SNRPA1	0.267	743	MGAT1	-0.218
744	TMEM126A	0.267	744	RP11.466H18.1	-0.218
745	PSMA3	0.267	745	NDUFV2P1	-0.218
746	ALG6	0.267	746	IL20RB	-0.218
747	ANAPC7	0.267	747	RP11.64B16.2	-0.218
748	DAXX	0.266	748	EEF1A1P12	-0.218
749	PSMB7	0.266	749	HOOK2	-0.218
750	HMGN1	0.266	750	RP11.46D6.1	-0.218
751	NDUFAF4	0.266	751	EIF4BP3	-0.219
752	ADAM11	0.266	752	AHSA2	-0.219
753	SDHC	0.266	753	POLR2J3	-0.219
754	ISOC1	0.266	754	SLC35C1	-0.219
755	RPS6KB1	0.266	755	IKBKGP1	-0.219
756	HNRNPU	0.266	756	RP11.730G20.[illegible]	-0.219
757	MTHFD1	0.266	757	TRAPPC12	-0.220
758	LYPLA1	0.265	758	RP11.1212A22	-0.220
759	METTL10	0.265	759	DGKZP1	-0.220
760	ATP5G1	0.265	760	MKNK2	-0.220

761	GOLGA7	0.265	761	PPIAP29	-0.220
762	IMMT	0.265	762	SUN2	-0.221
763	MCM4	0.265	763	CTD.2047H16.[illegible]	-0.221
764	ARL5A	0.265	764	ZBTB7A	-0.221
765	BARD1	0.265	765	RP11.567I13.1	-0.221
766	SRFBP1	0.265	766	RPL9P8	-0.221
767	RCN2	0.265	767	RP13.996F3.4	-0.221
768	IPO5	0.265	768	EIF4HP1	-0.222
769	DNM1L	0.265	769	D2HGDH	-0.222
770	CSTF3	0.264	770	AC027601.1	-0.222
771	ZNF670	0.264	771	MON1B	-0.222
772	CTPS1	0.264	772	RP11.571M6.7	-0.223
773	SNRPD3	0.264	773	RPL21P39	-0.223
774	FANCG	0.264	774	NR1D1	-0.223
775	GTPBP8	0.264	775	WASH2P	-0.223
776	UTP20	0.264	776	RPS26P47	-0.223
777	COA6	0.264	777	HERPUD1	-0.224
778	CDK2	0.264	778	TOM1	-0.224
779	PRC1.AS1	0.264	779	AGAP9	-0.224
780	COMMD2	0.263	780	PRKD2	-0.224
781	ZNF2	0.263	781	PPP1R14BP3	-0.224
782	GINS3	0.263	782	DGKA	-0.225
783	RP11.32B11.2	0.263	783	RPL7AP66	-0.225
784	MOB1A	0.263	784	DNAJC4	-0.225
785	CENPN	0.263	785	RP3.508I15.19	-0.225
786	XRCC2	0.263	786	RP11.449P15.2	-0.225
787	ANAPC10	0.263	787	PDCL3P4	-0.225
788	TMEM106C	0.263	788	ERAP1	-0.226
789	RMND1	0.263	789	SBNO2	-0.226
790	SENP6	0.263	790	TBC1D3F	-0.226
791	KPNA3	0.263	791	NEAT1	-0.226
792	SMU1	0.263	792	LA16c.366D1.[illegible]	-0.227
793	GTF2A2	0.263	793	RP4.706A16.3	-0.227
794	CDC20	0.262	794	RP5.1142A6.2	-0.227
795	DHFRP1	0.262	795	AQP3	-0.227
796	RP4.816N1.7	0.262	796	UNKL	-0.229
797	NDRG3	0.262	797	TCIRG1	-0.229
798	RP11.804A23.2	0.262	798	MIR611	-0.229
799	ZFYVE20	0.262	799	C16orf58	-0.230
800	PNPT1	0.262	800	MZF1	-0.230
801	PAFAH1B3	0.262	801	RP11.452L6.5	-0.230
802	METTL2B	0.262	802	EEF1A1P19	-0.231
803	SMN1	0.261	803	SLX1A	-0.231
804	PMS1	0.261	804	RP11.122K13.1	-0.231
805	UMPS	0.261	805	ZC3H12A	-0.231
806	UHRF1BP1	0.261	806	BRD7P2	-0.232
807	MSH6	0.261	807	AC093724.2	-0.232
808	HNRNPH3	0.261	808	SPPL2B	-0.232

809	ATG10	0.261	809	EEF1A1P11	-0.232
810	RWDD1	0.261	810	AC017116.11	-0.232
811	MUT	0.261	811	AGAP5	-0.232
812	JPX	0.261	812	STIM1	-0.232
813	TLK2	0.261	813	CTB.54D4.1	-0.232
814	DNTTIP2	0.261	814	EIF3FP3	-0.232
815	PRDX6	0.261	815	ZNF865	-0.232
816	RPS27	0.261	816	AC093106.7	-0.233
817	ENOPH1	0.261	817	AC079250.1	-0.233
818	HMMR	0.261	818	ADPGK.AS1	-0.233
819	RP11.386G11.1	0.261	819	MEF2D	-0.233
820	CHEK2	0.261	820	CTC.575D19.1	-0.234
821	RP11.265N6.2	0.260	821	FTH1P8	-0.234
822	RBBP4	0.260	822	NFKBIZ	-0.234
823	SMNDC1	0.260	823	OS9	-0.234
824	ZNF280C	0.260	824	PKD1	-0.235
825	RPA1	0.260	825	RP11.3P17.4	-0.235
826	RIOK2	0.260	826	C7orf43	-0.235
827	UBE3D	0.260	827	STAT2	-0.236
828	ARL6IP6	0.260	828	ARSA	-0.236
829	DGUOK	0.260	829	ERCC5	-0.237
830	EXOSC8	0.259	830	RP11.448G15.3	-0.237
831	URI1	0.259	831	GOLGA6L3	-0.238
832	DPH5	0.259	832	PSMB8	-0.238
833	TRMT5	0.259	833	B2M	-0.238
834	PEX3	0.259	834	RP11.75L1.2	-0.238
835	TOMM70A	0.259	835	SDCBP2	-0.238
836	CTD.2410N18.4	0.259	836	RP11.395B7.7	-0.238
837	KIAA1586	0.259	837	hsa.mir.6723	-0.238
838	ESF1	0.259	838	RP11.796E2.4	-0.238
839	SUPT16H	0.259	839	FAM3C2	-0.239
840	NAA25	0.259	840	ABTB1	-0.239
841	CHCHD3	0.259	841	UBXN6	-0.239
842	RBBP7	0.259	842	FLYWCH1	-0.239
843	C12orf4	0.259	843	NPIPA8	-0.240
844	SUZ12	0.259	844	LPCAT4	-0.240
845	WDR12	0.258	845	RP11.517A5.4	-0.241
846	ABT1	0.258	846	TUBGCP6	-0.242
847	MRPS18A	0.258	847	CALCOCO1	-0.242
848	DLEU2	0.258	848	LENG8	-0.242
849	SNRNP27	0.258	849	RPL18AP3	-0.242
850	TAF9	0.258	850	CTD.2015B23.[illegible]	-0.242
851	FAM49B	0.258	851	TMSB4XP8	-0.242
852	ACAT1	0.258	852	RP11.158M2.3	-0.243
853	SPATA5	0.257	853	RP11.393I23.2	-0.243
854	KIAA1191	0.257	854	RPL13AP20	-0.245
855	COPS3	0.257	855	C19orf60	-0.245
856	COX11	0.257	856	JMJD8	-0.245

857	C6orf211	0.257	857	WHAMM	-0.245
858	RRP1B	0.257	858	AC137934.1	-0.246
859	RTN4IP1	0.257	859	SLC9A8	-0.246
860	SMIM11	0.257	860	COX6A1P2	-0.246
861	PIGW	0.257	861	ZNF688	-0.247
862	U2SURP	0.257	862	FKBP11	-0.248
863	TRUB1	0.257	863	RPLP0P6	-0.248
864	ZNF639	0.257	864	CTD.2260A17.	-0.248
865	TRAIP	0.256	865	RPS26P3	-0.248
866	SLBP	0.256	866	YPEL3	-0.248
867	C1QBP	0.256	867	GTF2IRD2	-0.248
868	TMEM194A	0.256	868	TOLLIP	-0.249
869	SMARCA5	0.256	869	IKBKG	-0.249
870	CS	0.256	870	MIB2	-0.249
871	DTYMK	0.256	871	GDI2P2	-0.249
872	RP11.203B9.4	0.256	872	RP11.66N24.3	-0.249
873	HNRNPD	0.256	873	TEP1	-0.251
874	NUP188	0.256	874	TMEM175	-0.251
875	CTD.2555O16.2	0.256	875	CTD.2287O16.	-0.251
876	MAPRE1	0.255	876	CTD.2192J16.1	-0.251
877	RARS	0.255	877	RP11.592N21.1	-0.251
878	BRCA1	0.255	878	RPS11P5	-0.251
879	TATDN1	0.255	879	PDIA3P	-0.252
880		0.255	880	TECRP1	-0.252
881	FEN1	0.255	881	RN7SL4P	-0.252
882	CUL5	0.255	882	DNAH1	-0.253
883	ZNF184	0.255	883	TYMP	-0.253
884	TFB2M	0.255	884	EME2	-0.253
885	DNAJC7	0.255	885	PML	-0.253
886	RPL7	0.255	886	CHPF2	-0.254
887	GABPA	0.255	887	ST13P15	-0.255
888	DDX46	0.255	888	AP003419.11	-0.256
889	C2orf47	0.254	889	RAC1P2	-0.256
890	RNF219	0.254	890	RABEP2	-0.256
891	NKAP	0.254	891	UBA7	-0.256
892	CTB.96E2.7	0.254	892	RP11.543P15.1	-0.256
893	RPSA	0.254	893	RP11.304L19.4	-0.257
894	C12orf29	0.254	894	SPSB3	-0.257
895	NOP58	0.254	895	CDIPT	-0.258
896	NONO	0.254	896	RNPEPL1	-0.258
897	GNL2	0.254	897	NADSYN1	-0.258
898	ELP6	0.253	898	FBXL15	-0.258
899	MAPK9	0.253	899	RP11.386M24.4	-0.259
900	UBE2C	0.253	900	POLD4	-0.259
901	TIMM17A	0.253	901	EHBP1L1	-0.259
902	ZNF627	0.253	902	MVP	-0.259
903	CEP76	0.253	903	RP11.175B9.3	-0.259
904	PSMB6	0.253	904	PTK2B	-0.261

905	NUP88	0.253	905	ST13P6	-0.261
906	SIRT1	0.253	906	RP11.3P17.5	-0.261
907	RP4.694A7.2	0.252	907	RP11.286H14.4	-0.261
908	RPL10AP6	0.252	908	IRF1	-0.263
909	KRR1	0.252	909	PIK3IP1	-0.263
910	MRPL45	0.252	910	AC007238.1	-0.263
911	GTPBP4	0.252	911	UPF3AP1	-0.264
912	SNRPB2	0.252	912	ST13P19	-0.264
913	CHAC2	0.252	913	NPIPA3	-0.264
914	CASC5	0.252	914	ARMC5	-0.264
915	SPG21	0.252	915	TXNDC11	-0.265
916	RHOT1	0.252	916	AP006621.8	-0.267
917	DCUN1D1	0.252	917	HIST2H4A	-0.267
918	API5	0.252	918	AP5Z1	-0.267
919	TBC1D22B	0.252	919	MROH1	-0.267
920	UTP6	0.252	920	RBCK1	-0.267
921	GDI2	0.251	921	PPP1R12C	-0.267
922	GRPEL1	0.251	922	RPL3P4	-0.267
923	C8orf59	0.251	923	RP11.742N3.1	-0.268
924	ZC3H15	0.251	924	NOMO2	-0.269
925	TTC21B	0.251	925	PNPLA2	-0.269
926	RP11.345J4.5	0.251	926	RPS26P8	-0.269
927	PHB	0.251	927	FTH1P23	-0.270
928	SERF1B	0.251	928	RP11.166B2.1	-0.270
929	TOPBP1	0.251	929	RP11.50D9.1	-0.271
930	TBCC	0.251	930	RPL7AP11	-0.271
931	FAM35A	0.251	931	RP3.417G15.1	-0.272
932	CDCA8	0.251	932	RP11.168J18.6	-0.272
933	CCBL2	0.251	933	RP11.16F15.2	-0.272
934	GGCT	0.250	934	KIAA0247	-0.272
935	BAG6	0.250	935	AC092155.1	-0.273
936	CWF19L1	0.250	936	GS1.184P14.2	-0.273
937	CYCS	0.250	937	NLRC5	-0.273
938	GPSM2	0.250	938	RPL21P119	-0.274
939	VPS26A	0.250	939	RP11.475C16.1	-0.274
940	SKA1	0.250	940	ARAP1	-0.274
941	CSTF2	0.250	941	AC022431.1	-0.276
942	RP5.867C24.5	0.250	942	OSBPL7	-0.277
943	KPNA4	0.250	943	UPF3AP2	-0.277
944	TYMS	0.250	944	YIPF2	-0.277
945	FAM210A	0.250	945	C5orf56	-0.279
946	DHX29	0.250	946	RPL13AP5	-0.280
947	MRPL1	0.250	947	CTD.2031P19.4	-0.280
948	OPA1	0.250	948	EEF1B2P3	-0.281
949	DHX36	0.250	949	RP11.761N21.2	-0.281
950	ACAD9	0.250	950	AC093734.1	-0.281
951	ALMS1	0.250	951	SYVN1	-0.283
952	ZNF271	0.250	952	TMEM259	-0.283

953	RBM18	0.250
954	KPNA5	0.249
955	SRBD1	0.249
956	DDX31	0.249
957	HMGN2	0.249
958	ZNF326	0.249
959	RP11.697E22.2	0.249
960	UBE2L3	0.249
961	UTP23	0.249
962	TBPL1	0.249
963	CHEK1	0.249
964	CRYZL1	0.249
965	FANCL	0.249
966	HMGN4	0.249
967	NCL	0.248
968	RPL15	0.248
969	RP11.650L12.2	0.248
970	GINS2	0.248
971	GAPVD1	0.248
972	RPL22	0.248
973	EIF2B3	0.248
974	AZIN1	0.248
975	POLA2	0.248
976	C1orf109	0.248
977	EIF3I	0.248
978	ZNRD1	0.248
979	HILPDA	0.247
980	XRN2	0.247
981	RBBP5	0.247
982	WRB	0.247
983	EXOSC10	0.247
984	MRPS30	0.247
985	TRMT11	0.247
986	CKAP2L	0.247
987	ATP5H	0.247
988	DNAJC9.AS1	0.247
989	NAF1	0.246
990	HSPA8	0.246
991	MTFR1	0.246
992	TFDP2	0.246
993	STOML2	0.246
994	IDI1	0.246
995	UBTD2	0.246
996	NARG2	0.246
997	COX7B	0.246
998	ZFYVE16	0.246
999	ARHGAP19	0.246
1000	ATAD5	0.245

953	LINC00957	-0.284
954	PLEKHM1P	-0.286
955	SLC38A10	-0.288
956	MAFK	-0.290
957	AC116366.6	-0.290
958	MAP1LC3B2	-0.291
959	ZFP36	-0.292
960	RASA4B	-0.293
961	LRP10	-0.293
962	MT.ND6	-0.294
963	FTH1P2	-0.296
964	AP000936.1	-0.298
965	PCSK7	-0.299
966	TBC1D3C	-0.301
967	ARAP1.AS2	-0.303
968	C1orf132	-0.303
969	AC107983.4	-0.303
970	TTC7A	-0.303
971	PARP10	-0.304
972	RPL13AP7	-0.304
973	RP11.514P8.8	-0.305
974	CTD.3232M19.	-0.305
975	GTF2IRD2B	-0.307
976	RP11.680H20.1	-0.308
977	PLEKHM1	-0.309
978	PSMB10	-0.309
979	MAPK8IP3	-0.309
980	RPL13AP25	-0.315
981	RP11.680G24.4	-0.316
982	RASA4CP	-0.319
983	KB.1027C11.4	-0.321
984	CTB.134H23.2	-0.333
985	TAPBPL	-0.335
986	GNPTG	-0.336
987	RP11.109L13.1	-0.348
988	RP11.490O6.2	-0.351
989	TRADD	-0.353

1001	UBE2W	0.245
1002	TRMT1L	0.245
1003	FBXO9	0.245
1004	DCLRE1A	0.245
1005	HAUS3	0.245
1006	LSM14A	0.245
1007	RP11.423P10.2	0.245
1008	MRPL44	0.245
1009	CEP55	0.245
1010	HEATR1	0.245
1011	TUBG1	0.245
1012	CUL2	0.244
1013	ZBTB6	0.244
1014	FAM72B	0.244
1015	FANCD2	0.244
1016	CAPRIN1	0.244
1017	DHX40	0.244
1018	CKAP5	0.244
1019	POMP	0.244
1020	UQCR10	0.244
1021	HDAC8	0.244
1022	POLR2H	0.243
1023	POLR1B	0.243
1024	LANCL1	0.243
1025	FH	0.243
1026	PPP1R8	0.243
1027	MYL6B	0.243
1028	GPBP1	0.243
1029	SDHB	0.243
1030	PPT1	0.243
1031	SEC22C	0.243
1032	AAGAB	0.243
1033	NR2C2AP	0.242
1034	POLR2B	0.242
1035	CHRNA5	0.242
1036	ANP32E	0.242
1037	SPC24	0.242
1038	MED27	0.242
1039	SFXN1	0.242
1040	RP5.935K16.1	0.242
1041	MAPK14	0.242
1042	RBL1	0.242
1043	HDAC3	0.242
1044	PSMD8	0.242
1045	CLNS1A	0.241
1046	TMEM38B	0.241
1047	IFT81	0.241
1048	NANP	0.241

1049	TSFM	0.241
1050	FAM203A	0.241
1051	FASTKD2	0.241
1052	RPL5	0.240
1053	CDKAL1	0.240
1054	RNF219.AS1	0.240
1055	ALKBH1	0.240
1056	ZNF684	0.240
1057	PDCD2	0.240
1058	RQCD1	0.240
1059	SSRP1	0.240
1060	PHF3	0.240
1061	CCDC167	0.240
1062	RP11.101E13.5	0.240
1063	MAK16	0.240
1064	TADA1	0.240
1065	CDC123	0.240
1066	PIGX	0.240
1067	COPRS	0.240
1068	PRR3	0.239
1069	FAM20B	0.239
1070	FAM173B	0.239
1071	DDHD2	0.239
1072	AK2	0.239
1073	GSTCD	0.239
1074	KAT7	0.239
1075	POLR2G	0.239
1076	TAF8	0.239
1077	MORF4L2	0.239
1078	USP28	0.239
1079	FBXO28	0.238
1080	OAZ2	0.238
1081	MRPL40	0.238
1082	NCBP2.AS1	0.238
1083	TCOF1	0.238
1084	UHRF1BP1L	0.238
1085	TULP3	0.238
1086	RLIM	0.238
1087	CNIH4	0.238
1088	ANKRD40	0.238
1089	RPL29	0.238
1090	THAP5	0.238
1091	DIAPH3	0.238
1092	SNRNP48	0.238
1093	TTC27	0.237
1094	EBNA1BP2	0.237
1095	PRMT3	0.237
1096	ANP32A	0.237

1097	SMG8	0.237
1098	GLRX3	0.237
1099	GFM2	0.237
1100	URB2	0.237
1101	ZFR	0.237
1102	NCBP2	0.237
1103	RBX1	0.237
1104	TBC1D15	0.237
1105	RP11.294J22.6	0.237
1106	PPP3CB	0.237
1107	NUDCD2	0.237
1108	C17orf53	0.237
1109	RPL9	0.237
1110	RPL36	0.236
1111	TMEM201	0.236
1112	TNPO1	0.236
1113	MRPS7	0.236
1114	KCTD6	0.236
1115	MTF2	0.236
1116	SIKE1	0.236
1117	NIT2	0.236
1118	HMGCR	0.236
1119	TCEB1	0.235
1120	AGPAT5	0.235
1121	TIMM21	0.235
1122	PDSS1	0.235
1123	OSBPL11	0.235
1124	USMG5	0.235
1125	PHAX	0.235
1126	TTL	0.235
1127	C3orf17	0.235
1128	TRIAP1	0.235
1129	TRAM2.AS1	0.235
1130	ZNF273	0.235
1131	SS18L2	0.235
1132	TROAP	0.235
1133	RPL17	0.235
1134	DCTPP1	0.235
1135	PSMD6	0.235
1136	ZBTB2	0.235
1137	TCF19	0.234
1138	ZNF200	0.234
1139	PPIA	0.234
1140	IARS2	0.234
1141	SF3B5	0.234
1142	GTSE1	0.234
1143	PDS5A	0.234
1144	NVL	0.234

1145	MAP3K7	0.234
1146	TAF5L	0.234
1147	RP11.1079K10.	0.234
1148	VDAC1	0.234
1149	RWDD4P2	0.234
1150	MRFAP1	0.234
1151	ZZZ3	0.234
1152	MIR4426	0.233
1153	KB.1608C10.2	0.233
1154	TICRR	0.233
1155	EHMT2	0.233
1156	KIAA0895	0.233
1157	TMEM237	0.233
1158	RP11.603J24.17	0.233
1159	RP1.40E16.11	0.233
1160	CCDC41	0.233
1161	EIF2S2	0.233
1162	ZRANB3	0.233
1163	ABCD3	0.233
1164	CNBP	0.233
1165	WDR61	0.233
1166	HEATR6	0.233
1167	NOLC1	0.233
1168	GTF3C6	0.233
1169	SMYD5	0.232
1170	DDX10	0.232
1171	TCEB3	0.232
1172	MPHOSPH10	0.232
1173	MTCH1	0.232
1174	RPL35	0.232
1175	APEX2	0.232
1176	ZNF92	0.232
1177	MRPL24	0.232
1178	UQCRFS1	0.232
1179	KNTC1	0.232
1180	ARL6IP1	0.232
1181	WRAP53	0.232
1182	CCDC104	0.232
1183	RNF20	0.231
1184	RP11.631M6.2	0.231
1185	POLD3	0.231
1186	CCP110	0.231
1187	ADSS	0.231
1188	SENP1	0.231
1189	NCAPD2	0.231
1190	HDDC2	0.231
1191	HSBP1	0.231
1192	RYK	0.231

1193	EIF5B	0.230
1194	MRPL27	0.230
1195	RRP15	0.230
1196	VKORC1L1	0.230
1197	KIAA0196.AS1	0.230
1198	C1D	0.230
1199	PARP2	0.230
1200	TAF2	0.230
1201	WDR70	0.230
1202	ARHGEF39	0.230
1203	TGS1	0.230
1204	STK3	0.230
1205	H2AFV	0.229
1206	KLHL8	0.229
1207	DHX33	0.229
1208	PIN4	0.229
1209	DBT	0.229
1210	C14orf166	0.229
1211	RPL7A	0.229
1212	NOP16	0.229
1213	SEPHS1	0.229
1214	TIMELESS	0.229
1215	AURKB	0.229
1216	VPS72	0.229
1217	CWC22	0.229
1218	KIF22	0.228
1219	SUPV3L1	0.228
1220	YARS2	0.228
1221	C5orf51	0.228
1222	PTPN11	0.228
1223	DLD	0.228
1224	RPL35A	0.228
1225	COA3	0.228
1226	AARS2	0.228
1227	MDC1	0.228
1228	GEMIN4	0.228
1229	PSMD3	0.228
1230	PAK2	0.228
1231	THAP3	0.228
1232	DDX21	0.228
1233	OARD1	0.228
1234	NDUFA5	0.228
1235	ANGEL2	0.228
1236	SLC29A1	0.227
1237	UBA3	0.227
1238	CCSAP	0.227
1239	CAPZA1	0.227
1240	RSBN1	0.227

1241	RP11.161H23.9	0.227
1242	PIK3R4	0.227
1243	RP11.973D8.4	0.227
1244	RABEP1	0.227
1245	C17orf104	0.227
1246	DPH3	0.227
1247	SLC25A15	0.227
1248	PRPF38A	0.227
1249	MED4	0.226
1250	MPHOSPH6	0.226
1251	APPBP2	0.226
1252	FUBP3	0.226
1253	CISD2	0.226
1254	TGIF2	0.226
1255	HIBCH	0.226
1256	BOD1	0.226
1257	RP11.95D17.1	0.226
1258	ACOT13	0.226
1259	SMC6	0.226
1260	TTC33	0.226
1261	HN1	0.226
1262	RAD23B	0.226
1263	YWHAZ	0.226
1264	TACC3	0.225
1265	ABCF1	0.225
1266	NRAS	0.225
1267	UPF3B	0.225
1268	PDZD11	0.225
1269	FAM72D	0.225
1270	DDX52	0.225
1271	HAUS8	0.225
1272	TSNAX	0.225
1273	SLC25A32	0.225
1274	TOPORS	0.225
1275	COMMD1	0.225
1276	TPI1	0.225
1277	ZNF138	0.224
1278	POLR3G	0.224
1279	SLC25A3	0.224
1280	PRMT5.AS1	0.224
1281	PSMB5	0.224
1282	HSPH1	0.224
1283	MCM2	0.224
1284	PANK3	0.224
1285	RP3.337H4.9	0.224
1286	FANCM	0.224
1287	METTL14	0.224
1288	SHMT1	0.224

1289	TTI1	0.224
1290	RWDD4P1	0.224
1291	PSME3	0.224
1292	USP13	0.224
1293	C1orf52	0.223
1294	RP11.802O23.3	0.223
1295	ATP6V1C1	0.223
1296	GRPEL2	0.223
1297	ITGB1BP1	0.223
1298	RP11.256L6.2	0.223
1299	PTCD3	0.223
1300	ARPP19	0.223
1301	WDR43	0.223
1302	COX6A1	0.223
1303	AC091133.1	0.223
1304	POLR2K	0.223
1305	BMPR1A	0.223
1306	PITPNB	0.223
1307	RP1.30M3.5	0.223
1308	TMEM206	0.223
1309	PTBP2	0.223
1310	GTF2E1	0.223
1311	GIN1	0.223
1312	PARP1	0.222
1313	LAS1L	0.222
1314	RBBP9	0.222
1315	MDC1.AS1	0.222
1316	LYAR	0.222
1317	ESPL1	0.222
1318	AC002550.5	0.222
1319	ACER3	0.222
1320	HTATSF1	0.222
1321	UBE3A	0.222
1322	FGD5.AS1	0.222
1323	C18orf54	0.222
1324	ATPAF1	0.222
1325	TROVE2	0.222
1326	BPHL	0.222
1327	CTDSPL2	0.222
1328	FARS2	0.222
1329	ZNF638	0.222
1330	DNAJC9	0.222
1331	MNAT1	0.222
1332	PPIH	0.221
1333	ACP1	0.221
1334	ESD	0.221
1335	ILKAP	0.221
1336	CTC.534A2.2	0.221

1337	NLE1	0.221
1338	CCNE1	0.221
1339	GSPT1	0.221
1340	LYRM7	0.221
1341	NMD3	0.221
1342	BOLA2B	0.221
1343	ABCB7	0.221
1344	RP5.886K2.3	0.221
1345	DPH6	0.221
1346	SUB1	0.221
1347	PRKAR2B	0.221
1348	FBL	0.221
1349	RP5.874C20.3	0.221
1350	TMEM199	0.221
1351	UFSP2	0.221
1352	C11orf73	0.220
1353	ANLN	0.220
1354	SRSF9	0.220
1355	STAU1	0.220
1356	HS2ST1	0.220
1357	SNF8	0.220
1358	ATL2	0.220
1359	NBPF14	0.220
1360	RPL12	0.220
1361	SPAG5.AS1	0.220
1362	NDUFB2.AS1	0.220
1363	PPP2R5E	0.220
1364	EI24	0.220
1365	ARL13B	0.220
1366	ATIC	0.219
1367	AP3M1	0.219
1368	RPAP2	0.219
1369	CCNI	0.219
1370	ADNP2	0.219
1371	CXorf56	0.219
1372	ATP5J2	0.219
1373	MEAF6	0.219
1374	DAP3	0.218
1375	GTF2A1	0.218
1376	PSMD1	0.218
1377	GKAP1	0.218
1378	NAP1L1	0.218
1379	UBE2K	0.218
1380	HNRNPA0	0.218
1381	LINC00094	0.218
1382	INTS7	0.218
1383	NUP160	0.218
1384	DCUN1D5	0.218

1385	RP11.98D18.15	0.217
1386	BMS1	0.217
1387	CENPM	0.217
1388	BEND3	0.217
1389	ASUN	0.217
1390	LIN52	0.217
1391	RABEPK	0.217
1392	APPL1	0.217
1393	RFC1	0.217
1394	TBP	0.217
1395	PRKDC	0.217
1396	ACVR2B	0.217
1397	RP11.145M9.4	0.217
1398	CBX2	0.217
1399	PPA1	0.217
1400	NDUFV2	0.217
1401	TAF1A	0.217
1402	CMC2	0.217
1403	MGME1	0.216
1404	USP45	0.216
1405	MICU2	0.216
1406	UBE2S	0.216
1407	CWC27	0.216
1408	MFN1	0.216
1409	KIAA1841	0.216
1410	RLF	0.216
1411	RAD51	0.216
1412	RP11.21J18.1	0.216
1413	HIGD1A	0.216
1414	HSD17B10	0.216
1415	IFT52	0.216
1416	GTF3C3	0.216
1417	TXNDC9	0.216
1418	KCTD20	0.216
1419	CDCA5	0.216
1420	FAF1	0.216
1421	NENF	0.215
1422	ADIPOR2	0.215
1423	CHD1	0.215
1424	TRIM45	0.215
1425	PPP2R2A	0.215
1426	KIF18B	0.215
1427	MIR302B	0.215
1428	NRD1	0.215
1429	RABL3	0.215
1430	AC073641.2	0.215
1431	SAR1A	0.215
1432	FAM178A	0.214

1433	STRADB	0.214
1434	FOXM1	0.214
1435	DIABLO	0.214
1436	CPSF2	0.214
1437	RPL14	0.214
1438	RP5.1172N10.4	0.214
1439	RP11.186N15.3	0.214
1440	DKC1	0.214
1441	GPR137C	0.214
1442	RBM14	0.214
1443	RPUSD3	0.214
1444	HSDL2	0.213
1445	TIMMDC1	0.213
1446	RP11.155G14.6	0.213
1447	POLE3	0.213
1448	RFWD3	0.213
1449	SNX27	0.213
1450	NOP10	0.213
1451	FAM118B	0.213
1452	RAC3	0.213
1453	PPM1D	0.213
1454	RBMX2	0.213
1455	SAMD1	0.213
1456	FAM172A	0.212
1457	AC099850.1	0.212
1458	KIF2A	0.212
1459	ARL3	0.212
1460	PRPF4B	0.212
1461	UHRF1	0.212
1462	EEF1A1	0.212
1463	FUS	0.212
1464	ELP4	0.212
1465	PIGA	0.212
1466	NEIL2	0.212
1467	IFT88	0.212
1468	SNAPIN	0.212
1469	RPP14	0.212
1470	RCC1	0.211
1471	FGFR1OP	0.211
1472	TMA16	0.211
1473	ATRIP	0.211
1474	RN7SL2	0.211
1475	IPO9.AS1	0.211
1476	FBXO22	0.211
1477	PHF20	0.211
1478	REPS1	0.211
1479	SLC30A6	0.211
1480	CTD.2008A1.2	0.211

1481	HINT3	0.211
1482	MRPL2	0.211
1483	RNPS1	0.211
1484	DNAJC19	0.211
1485	C12orf45	0.211
1486	HLTF	0.211
1487	SERF1A	0.211
1488	NOL9	0.211
1489	PANK1	0.210
1490	ZNF131	0.210
1491	MED21	0.210
1492	GNPAT	0.210
1493	EIF4A3	0.210
1494	AZI2	0.210
1495	NME7	0.210
1496	ASPM	0.210
1497	RBM8A	0.210
1498	PPIG	0.210
1499	PA2G4	0.210
1500	NHP2	0.210
1501	CHORDC1	0.210
1502	GDAP2	0.210
1503	MMADHC	0.210
1504	RANBP9	0.210
1505	MTPAP	0.210
1506	PCCB	0.210
1507	ASF1A	0.209
1508	RPL32	0.209
1509	AC006011.4	0.209
1510	STYX	0.209
1511	GNPNAT1	0.209
1512	TRAPPC13	0.209
1513	FAM133B	0.209
1514	RPIA	0.209
1515	SEC22A	0.209
1516	FOPNL	0.209
1517	RP5.930J4.4	0.209
1518	TTF2	0.209
1519	CCDC23	0.209
1520	KPNA6	0.209
1521	GLRX2	0.209
1522	RSL1D1	0.209
1523	G3BP2	0.209
1524	NKRF	0.209
1525	VPS54	0.208
1526	METTL18	0.208
1527	PRPSAP2	0.208
1528	CCHCR1	0.208

1529	CCDC43	0.208
1530	PKN2	0.208
1531	RP11.266K22.2	0.208
1532	HMGB2	0.208
1533	ZNF318	0.208
1534	PDE12	0.208
1535	RPL10A	0.207
1536	ZCCHC10	0.207
1537	GTF2F2	0.207
1538	RP11.90P5.2	0.207
1539	AC007620.3	0.207
1540	FAM96A	0.207
1541	PWP2	0.207
1542	APTX	0.207
1543	INTS2	0.207
1544	POC1B	0.207
1545	METTL4	0.207
1546	PRKAR2A	0.207
1547	CRIPT	0.207
1548	ACACA	0.207
1549	C12orf73	0.207
1550	SEC61A2	0.206
1551	PARG	0.206
1552	ST7L	0.206
1553	RCHY1	0.206
1554	DDX1	0.206
1555	POGK	0.206
1556	GNPDA1	0.206
1557	UQCRQ	0.206
1558	FPGT	0.206
1559	CUL1	0.206
1560	SHQ1	0.206
1561	PARK7	0.206
1562	DPY30	0.206
1563	RPL23	0.206
1564	PAFAH1B2	0.206
1565	PRUNE	0.206
1566	POP1	0.206
1567	COPS8	0.206
1568	WDR82	0.205
1569	PSMD13	0.205
1570	TRUB2	0.205
1571	MIR4721	0.205
1572	PTRH2	0.205
1573	KIF1B	0.205
1574	AC005740.5	0.205
1575	NANOS1	0.205
1576	CDK19	0.205

1577	MAML1	0.205
1578	PIP4K2B	0.205
1579	MED31	0.205
1580	ZNF678	0.205
1581	ELK1	0.205
1582	TMEM14A	0.205
1583	MPHOSPH9	0.205
1584	COA1	0.205
1585	SRF	0.205
1586	SUGT1	0.204
1587	DYNC2H1	0.204
1588	C16orf80	0.204
1589	PSMC3IP	0.204
1590	SNAP47	0.204
1591	KB.1043D8.6	0.204
1592	MTMR12	0.204
1593	C6orf57	0.204
1594	UBAC2.AS1	0.204
1595	YRDC	0.204
1596	CDC73	0.204
1597	UBQLN1	0.204
1598	VPS29	0.204
1599	PLK1	0.204
1600	NCAPD3	0.204
1601	UBE2D1	0.204
1602	MAD2L2	0.204
1603	HN1L	0.204
1604	MOCS2	0.204
1605	BORA	0.204
1606	COX6C	0.204
1607	AKAP5	0.204
1608	PBX2	0.203
1609	SLMO2	0.203
1610	NBPF16	0.203
1611	CTD.2636A23.[illegible]	0.203
1612	KIF5B	0.203
1613	TCAIM	0.203
1614	RP11.303E16.2	0.203
1615	CSTF1	0.203
1616	AQR	0.203
1617	DCK	0.203
1618	USP37	0.203
1619	C19orf40	0.203
1620	C14orf142	0.203
1621	TIMM9	0.203
1622	C14orf2	0.203
1623	C3orf38	0.202
1624	NTMT1	0.202

1625	RP5.1136G13.2	0.202
1626	NFYB	0.202
1627	NAA35	0.202
1628	TPR	0.202
1629	TERF1	0.202
1630	NUTF2	0.202
1631	SNRPD2	0.202
1632	TSR1	0.202
1633	PTCD2	0.202
1634	SNX2	0.202
1635	DDX3X	0.202
1636	SMARCE1	0.201
1637	CRNKL1	0.201
1638	RP11.299J3.8	0.201
1639	KIAA1958	0.201
1640	CRBN	0.201
1641	AL132780.1	0.201
1642	TRIM33	0.201
1643	NELFCD	0.201
1644	RFK	0.201
1645	DOCK7	0.201
1646	EIF4A1	0.201
1647	LRRC42	0.201
1648	FAM162A	0.201
1649	USP16	0.201
1650	NSMCE2	0.201
1651	MED28	0.201
1652	RP11.97O12.7	0.200
1653	FAM45A	0.200
1654	MTIF2	0.200
1655	NUCKS1	0.200
1656	E2F1	0.200
1657	NDUFB11	0.200
1658	SPIN1	0.200
1659	RTF1	0.200
1660	CETN2	0.200
1661	FGD1	0.200
1662	YES1	0.200
1663	ALDH9A1	0.200
1664	OGFOD1	0.200
1665	SNX12	0.200
1666	TXLNG	0.200
1667	PHTF2	0.200
1668	FAM229B	0.200
1669	MRPS36	0.199
1670	NARS2	0.199
1671	DYNLL1	0.199
1672	DNAL1	0.199

1673	AC004257.3	0.199
1674	TMEM69	0.199
1675	ADH5	0.199
1676	TCFL5	0.199
1677	RP11.592B15.4	0.199
1678	TDP2	0.199
1679	TTF1	0.199
1680	ZUFSP	0.199
1681	EXOC6	0.199
1682	ABI2	0.198
1683	EIF1B	0.198
1684	QSER1	0.198
1685	RP4.657D16.3	0.198
1686	GPD1L	0.198
1687	NIP7	0.198
1688	RPE	0.198
1689	ADO	0.198
1690	USP32	0.198
1691	AMZ2	0.198
1692	RP11.804A23.4	0.198
1693	ATP5C1	0.198
1694	NEIL3	0.198
1695	PRPSAP1	0.197
1696	PDHB	0.197
1697	ARL2	0.197
1698	DUS4L	0.197
1699	MRPL9	0.197
1700	ZNF510	0.197
1701	EID2	0.197
1702	EZH2	0.197
1703	NUP50	0.197
1704	FAM103A1	0.197
1705	AC083799.1	0.197
1706	PSMD4	0.197
1707	TBK1	0.197
1708	HJURP	0.196
1709	LSM1	0.196
1710	THRAP3	0.196
1711	TUBA1C	0.196
1712	ANAPC1	0.196
1713	SUCLA2	0.196
1714	KIAA1731	0.196
1715	VWA9	0.196
1716	MAPK8	0.196
1717	PHF10	0.196
1718	EXOC5	0.196
1719	BTRC	0.196
1720	RPS6KC1	0.196

1721	B3GALTL	0.196
1722	SMAD5	0.196
1723	DARS	0.196
1724	ZNF507	0.196
1725	TCEANC2	0.196
1726	ARL5B	0.196
1727	TMA7	0.196
1728	VPS45	0.196
1729	MMGT1	0.196
1730	RP1.228H13.5	0.196
1731	RP11.589M4.1	0.196
1732	ZMYM3	0.195
1733	CEP97	0.195
1734	MED14	0.195
1735	CMC1	0.195
1736	OTUD6B	0.195
1737	UBP1	0.195
1738	UFD1L	0.195
1739	AP3M2	0.195
1740	ABCA11P	0.195
1741	PDS5B	0.195
1742	VPS35	0.195
1743	WDR47	0.195
1744	SRP72	0.195
1745	EIF3M	0.195
1746	ALKBH2	0.195
1747	NUDT5	0.195
1748	C17orf96	0.195
1749	EFCAB11	0.195
1750	CFL2	0.194
1751	MIR4519	0.194
1752	ZNF124	0.194
1753	IMMP1L	0.194
1754	ASF1B	0.194
1755	TRNT1	0.194
1756	RPRD1A	0.194
1757	NDUFA4	0.194
1758	AF196970.3	0.194
1759	SORD	0.194
1760	DPY19L4	0.194
1761	ZNF286B	0.194
1762	ENO1P4	0.194
1763	TRAPPC3	0.194
1764	EIF3H	0.194
1765	WDR33	0.193
1766	FAM217B	0.193
1767	PACRGL	0.193
1768	TMEM209	0.193

1769	PEBP1	0.193
1770	AHSA1	0.193
1771	CDK5RAP2	0.193
1772	CTNNAL1	0.193
1773	NDUFAF7	0.193
1774	RPL35P5	0.193
1775	RPL18A	0.193
1776	PROSC	0.193
1777	BTF3	0.193
1778	STAMBP	0.193
1779	RRP9	0.193
1780	SPOP	0.193
1781	ACADSB	0.193
1782	ZNF16	0.193
1783	UBE2F	0.193
1784	C9orf40	0.193
1785	ZNF691	0.193
1786	GGPS1	0.193
1787	TTC7B	0.193
1788	CLASP2	0.192
1789	C18orf21	0.192
1790	UBR7	0.192
1791	FAM169A	0.192
1792	RB1CC1	0.192
1793	RPL27	0.192
1794	FDPS	0.192
1795	EED	0.192
1796	ZNF721	0.192
1797	NACA	0.192
1798	MAPK1IP1L	0.192
1799	ZBTB33	0.192
1800	FAM60A	0.192
1801	TRMT6	0.192
1802	FMR1	0.192
1803	SYNJ2BP.COX	0.192
1804	CCNG1	0.191
1805	TP53RK	0.191
1806	CRLF3	0.191
1807	GPR89A	0.191
1808	YME1L1	0.191
1809	PRKRIR	0.191
1810	C6orf203	0.191
1811	ABHD10	0.191
1812	TYW3	0.191
1813	AGTPBP1	0.191
1814	CCDC86	0.191
1815	RP11.343N15.5	0.191
1816	MCM7	0.191

1817	CENPP	0.191
1818	METAP1	0.191
1819	USP5	0.191
1820	RP11.872D17.4	0.191
1821	HNRNPL	0.191
1822	UBAP2L	0.191
1823	PAXBP1	0.191
1824	EIF2S3	0.190
1825	KLHL15	0.190
1826	RP11.545I5.3	0.190
1827	RBM28	0.190
1828	SDCCAG3	0.190
1829	BUB3	0.190
1830	PHOSPHO2	0.190
1831	KIAA1467	0.190
1832	OLA1P1	0.190
1833	GTF2H2C	0.190
1834	FAM72A	0.190
1835	ICT1	0.190
1836	TIMM10	0.190
1837	ALYREF	0.189
1838	ZFP69B	0.189
1839	ACLY	0.189
1840	BTBD9	0.189
1841	GMEB1	0.189
1842	LAMTOR5	0.189
1843	RP11.216B9.6	0.189
1844	TDRKH	0.189
1845	NBPF1	0.189
1846	PRPF19	0.189
1847	DEGS1	0.189
1848	ZFAND3	0.189
1849	XPO4	0.189
1850	BCKDHB	0.189
1851	PSMG2	0.189
1852	CNIH1	0.189
1853	EXTL2	0.189
1854	QRSL1	0.189
1855	MFF	0.189
1856	RP3.469D22.1	0.189
1857	PRMT5	0.189
1858	BTG3	0.189
1859	BRCC3	0.189
1860	MED6	0.189
1861	RAB28	0.189
1862	SDHD	0.189
1863	ABCF2	0.189
1864	BBS7	0.189

1865	MANEA	0.189
1866	TRIM23	0.189
1867	C8orf33	0.188
1868	CHRAC1	0.188
1869	RPS17L	0.188
1870	FAM8A1	0.188
1871	UCK2	0.188
1872	SFR1	0.188
1873	RP13.131K19.1	0.188
1874	CREM	0.188
1875	RPL26L1	0.188
1876	POLQ	0.188
1877	GCAT	0.188
1878	RP11.452F19.3	0.188
1879	IDI2.AS1	0.188
1880	RAD17	0.188
1881	NDUFAB1	0.188
1882	39142.00	0.188
1883	ROCK1P1	0.188
1884	DPH3P1	0.188
1885	TADA2A	0.188
1886	TXNDC16	0.188
1887	ZNF664	0.188
1888	MED1	0.188
1889	ECI2	0.188
1890	AMZ2P1	0.188
1891	FXN	0.188
1892	CHML	0.188
1893	PDCD5	0.188
1894	RP11.613M10.6	0.187
1895	MRPS2	0.187
1896	CACUL1	0.187
1897	GALNT11	0.187
1898	METTL5	0.187
1899	ZC3H14	0.187
1900	ACTR3B	0.187
1901	HSPA9	0.187
1902	IKBKAP	0.187
1903	C16orf59	0.187
1904	SAP130	0.187
1905	NDUFS3	0.187
1906	ZNF189	0.187
1907	WDSUB1	0.187
1908	STRN	0.187
1909	RP11.388M20.6	0.187
1910	USP46	0.187
1911	SRRD	0.187
1912	RPL11	0.187

1913	IREB2	0.187
1914	SP3	0.187
1915	YAE1D1	0.187
1916	CPNE3	0.187
1917	MAST1	0.186
1918	MYNN	0.186
1919	U2AF1	0.186
1920	PSMC2	0.186
1921	TAB2	0.186
1922	TWISTNB	0.186
1923	SMARCAD1	0.186
1924	QRICH1	0.186
1925	ASTE1	0.186
1926	PARS2	0.186
1927	NPAT	0.186
1928	SLC30A9	0.186
1929	PXMP2	0.186
1930	PAXIP1	0.186
1931	PHIP	0.186
1932	POLR2F	0.186
1933	PDHA1	0.186
1934	FLVCR1	0.186
1935	MRPS31	0.186
1936	SMC1A	0.186
1937	GABPB1	0.186
1938	RAB11A	0.185
1939	TMEM68	0.185
1940	TAF13	0.185
1941	EIF2A	0.185
1942	EXOC2	0.185
1943	EME1	0.185
1944	ATP5B	0.185
1945	SPDL1	0.185
1946	PPHLN1	0.185
1947	ENSA	0.185
1948	GDPD1	0.185
1949	KIAA1430	0.185
1950	SUV39H1	0.185
1951	LRRC8B	0.185
1952	TMEM70	0.185
1953	KTN1.AS1	0.185
1954	DCTN6	0.185
1955	COPG2	0.185
1956	AIFM1	0.185
1957	GNRHR2P1	0.185
1958	SEPN1	0.184
1959	PPP2R1B	0.184
1960	DTD2	0.184

1961	CASC3	0.184
1962	EFCAB7	0.184
1963	DCP2	0.184
1964	HMGB1P5	0.184
1965	THOC7	0.184
1966	DHX15	0.184
1967	CISD1	0.184
1968	CMTR1	0.184
1969	RP11.20B24.4	0.184
1970	DLAT	0.183
1971	KIAA0020	0.183
1972	CLTC	0.183
1973	SOD1	0.183
1974	CSNK1G3	0.183
1975	C12orf65	0.183
1976	PRKRA	0.183
1977	AIMP1	0.183
1978	CEP70	0.183
1979	RP11.452H21.1	0.183
1980	UBE2D2	0.183
1981	RAF1	0.183
1982	RP11.365O16.6	0.183
1983	METTL8	0.183
1984	TRDMT1	0.183
1985	RNF144A	0.183
1986	LATS1	0.183
1987	LSM4	0.183
1988	CIT	0.183
1989	CDK12	0.183
1990	MRPS16	0.183
1991	NFIB	0.183
1992	PUF60	0.183
1993	AC144449.1	0.182
1994	PREP	0.182
1995	CGRRF1	0.182
1996	FNBP1L	0.182
1997	VAMP7	0.182
1998	KARS	0.182
1999	ACTR2	0.182
2000	DBF4B	0.182
2001	PPIAP11	0.182
2002	RNF11	0.182
2003	FBXW11	0.182
2004	STRA13	0.182
2005	SP4	0.182
2006	AGBL5	0.182
2007	YTHDC2	0.182
2008	MIS18BP1	0.182

2009	SCYL2	0.182
2010	MRE11A	0.182
2011	AP000320.7	0.182
2012	ABCB10	0.181
2013	C10orf88	0.181
2014	METTL9	0.181
2015	FAM216A	0.181
2016	POLDIP2	0.181
2017	RP11.1094M14.	0.181
2018	SLC25A19	0.181
2019	FAM200A	0.181
2020	CRK	0.181
2021	TDG	0.181
2022	CBY1	0.181
2023	KIAA1009	0.181
2024	ZBTB26	0.181
2025	NBPF20	0.181
2026	SF3B3	0.181
2027	OSBPL8	0.181
2028	LSM6	0.181
2029	MSL1	0.181
2030	C3orf33	0.181
2031	SLC25A11	0.181
2032	RP11.773H22.4	0.181
2033	MTX1	0.181
2034	TK1	0.181
2035	LTA4H	0.181
2036	SLC5A6	0.181
2037	YBX1	0.181
2038	SERAC1	0.181
2039	MED30	0.181
2040	PLCG1	0.180
2041	NUP98	0.180
2042	UBLCP1	0.180
2043	KLHL12	0.180
2044	RP11.315I20.1	0.180
2045	KIAA0947	0.180
2046	PSMA6	0.180
2047	CTD.2026K11.2	0.180
2048	RCL1	0.180
2049	NPM3	0.180
2050	NDUFA12	0.180
2051	THAP9	0.180
2052	MRPL36	0.180
2053	THOC3	0.180
2054	NDUFA9	0.180
2055	HNRNPF	0.180
2056	RALA	0.180

2057	INTS9	0.180
2058	RP11.316M1.12	0.180
2059	SFPQ	0.180
2060	Sep-07	0.179
2061	TCERG1	0.179
2062	XRCC6BP1	0.179
2063	LLPH	0.179
2064	DUSP12	0.179
2065	PSMD10	0.179
2066	FRS2	0.179
2067	RAC1	0.179
2068	SF3B4	0.179
2069	LMBR1	0.179
2070	PAPOLG	0.179
2071	PHF16	0.179
2072	UBXN7	0.179
2073	MIS12	0.179
2074	FUT10	0.179
2075	TOMM40	0.179
2076	SLC25A30	0.179
2077	ACOT7	0.178
2078	FAM53C	0.178
2079	UBL7.AS1	0.178
2080	TMEM67	0.178
2081	RTCA	0.178
2082	TIMM50	0.178
2083	NT5C3B	0.178
2084	RPS28	0.178
2085	TTC1	0.178
2086	GRSF1	0.178
2087	GPALPP1	0.178
2088	ERCC6L2	0.178
2089	SNW1	0.178
2090	RNFT1	0.177
2091	IPO8	0.177
2092	AFG3L2	0.177
2093	AC109828.1	0.177
2094	HCCS	0.177
2095	DTNBP1	0.177
2096	LARS2	0.177
2097	FIGNL1	0.177
2098	PSMA7	0.177
2099	UBL7	0.177
2100	ERCC3	0.177
2101	CCNF	0.177
2102	RWDD3	0.177
2103	TPMT	0.177
2104	DTNB	0.177

2105	LMNB2	0.177
2106	SLC25A5.AS1	0.177
2107	PCM1	0.177
2108	LPHN1	0.177
2109	BRK1	0.176
2110	RPS2	0.176
2111	DNAJC2	0.176
2112	MERTK	0.176
2113	SUPT3H	0.176
2114	UBQLN4	0.176
2115	STAU2	0.176
2116	AP2B1	0.176
2117	HMGCS1	0.176
2118	UBA1	0.176
2119	CNOT11	0.176
2120	PIK3R3	0.176
2121	WDR36	0.176
2122	RP13.638C3.4	0.176
2123	DR1	0.176
2124	C1orf174	0.176
2125	NHP2L1	0.176
2126	BNIP1	0.176
2127	KBTBD7	0.176
2128	FAM175A	0.176
2129	ERLIN1	0.176
2130	AKIRIN1	0.176
2131	NDUFAF2	0.176
2132	ATP5G2	0.176
2133	NRM	0.176
2134	RP11.1033A18.	0.175
2135	DTWD2	0.175
2136	C5orf54	0.175
2137	KIAA1279	0.175
2138	MRPL17	0.175
2139	TRIM52.AS1	0.175
2140	ACTR10	0.175
2141	C1orf216	0.175
2142	HNRNPDL	0.175
2143	WRN	0.175
2144	PLEKHA8	0.175
2145	VARS	0.175
2146	CLP1	0.175
2147	HEATR2	0.175
2148	PMPCB	0.175
2149	CWC15	0.175
2150	MYBL2	0.175
2151	CHD1L	0.175
2152	CUTA	0.175

2153	SUCLG1	0.174
2154	NSDHL	0.174
2155	FAM200B	0.174
2156	RMDN1	0.174
2157	ODF2	0.174
2158	VPS52	0.174
2159	MTFMT	0.174
2160	TAOK1	0.174
2161	COQ7	0.174
2162	POP7	0.174
2163	WEE1	0.174
2164	PFDN1	0.174
2165	SMCHD1	0.174
2166	RP11.61A14.3	0.174
2167	KM.PA.2	0.174
2168	ADNP	0.174
2169	NOP56	0.174
2170	SCLT1	0.174
2171	BRD3	0.174
2172	MAPT	0.173
2173	ANP32B	0.173
2174	CNIH2	0.173
2175	TACO1	0.173
2176	XXYLT1	0.173
2177	AC010127.5	0.173
2178	RP11.231C14.3	0.173
2179	LONRF1	0.173
2180	HMGB3	0.173
2181	ILF3	0.173
2182	GPANK1	0.173
2183	GDAP1	0.173
2184	TSEN34	0.173
2185	PPP2R3C	0.173
2186	ALG10	0.173
2187	CEP192	0.173
2188	EMC2	0.173
2189	FAM204A	0.172
2190	FAM92A1	0.172
2191	VPS25	0.172
2192	RP11.282O18.3	0.172
2193	AASDH	0.172
2194	BUD31	0.172
2195	PLRG1	0.172
2196	CDK5	0.172
2197	BRCA2	0.172
2198	VAPB	0.172
2199	NUDC	0.172
2200	ARPC3	0.172

2201	MKS1	0.172
2202	DBI	0.172
2203	RNFT2	0.172
2204	MRPL21	0.172
2205	RP11.253M7.1	0.172
2206	PGBD1	0.172
2207	PSMC4	0.172
2208	MKLN1	0.172
2209	PHB2	0.172
2210	TOP2B	0.171
2211	RBM12B	0.171
2212	PARL	0.171
2213	BCL7C	0.171
2214	CEP85	0.171
2215	ARPC1A	0.171
2216	DHODH	0.171
2217	TOE1	0.171
2218	DYNC1I2	0.171
2219	POLE	0.171
2220	RPS4X	0.171
2221	TTI2	0.171
2222	NDUFB9	0.171
2223	NBN	0.171
2224	CEP135	0.171
2225	R3HDM1	0.171
2226	MBLAC2	0.171
2227	ITSN1	0.171
2228	RP11.889L3.1	0.171
2229	Sep-02	0.171
2230	PALB2	0.170
2231	E2F5	0.170
2232	ZNF322	0.170
2233	COX16	0.170
2234	MIER3	0.170
2235	PBRM1	0.170
2236	NQO2	0.170
2237	STRBP	0.170
2238	STAG2	0.170
2239	PMPCA	0.170
2240	GTF3C2	0.170
2241	BTBD1	0.170
2242	HACL1	0.170
2243	MYEOV2	0.170
2244	EIF3L	0.170
2245	SYAP1	0.170
2246	TARS	0.170
2247	AL358781.1	0.169
2248	SETP14	0.169

2249	NXT1	0.169
2250	FAM175B	0.169
2251	AL139819.1	0.169
2252	ASIC1	0.169
2253	CCAR1	0.169
2254	NDUFB3	0.169
2255	C11orf58	0.169
2256	TMTC3	0.169
2257	PDCD2L	0.169
2258	MCM5	0.169
2259	ENTPD1.AS1	0.169
2260	ATP5L	0.169
2261	WASF1	0.169
2262	MRPL48	0.169
2263	PGGT1B	0.169
2264	TMEM126B	0.169
2265	RP11.815I9.4	0.169
2266	GFM1	0.169
2267	TCEA1	0.168
2268	RNMTL1	0.168
2269	LRRC20	0.168
2270	FOXK2	0.168
2271	AC004381.6	0.168
2272	SEC23IP	0.168
2273	NDUFB2	0.168
2274	CIAPIN1	0.168
2275	MRPL33	0.168
2276	ING2	0.168
2277	CHAF1A	0.168
2278	LSM14B	0.168
2279	PPA2	0.168
2280	RP11.391M1.4	0.168
2281	POC5	0.168
2282	CCDC25	0.168
2283	AGO1	0.168
2284	HIPK1	0.168
2285	MPLKIP	0.168
2286	LBR	0.168
2287	SLC39A10	0.167
2288	CHCHD6	0.167
2289	TRMT112	0.167
2290	BDP1	0.167
2291	HCG25	0.167
2292	SNX14	0.167
2293	PIGP	0.167
2294	C6orf106	0.167
2295	MRPS35	0.167
2296	PINX1	0.167

2297	MDM1	0.167
2298	SRRM1	0.167
2299	FOXRED2	0.167
2300	RFWD2	0.167
2301	RSRC1	0.167
2302	IQCB1	0.167
2303	USP10	0.167
2304	APOA1BP	0.167
2305	IMPDH2	0.167
2306	WASH5P	0.167
2307	FAM189B	0.167
2308	RMI2	0.167
2309	RWDD2A	0.166
2310	TRIM24	0.166
2311	SLC25A14	0.166
2312	TSEN2	0.166
2313	TMEM242	0.166
2314	RP11.603J24.14	0.166
2315	THOC5	0.166
2316	NDUFAF6	0.166
2317	NDUFS5	0.166
2318	DLEU1	0.166
2319	FAM210B	0.166
2320	PRKCI	0.166
2321	ZNF107	0.166
2322	FOXP4	0.166
2323	AC002117.1	0.166
2324	ISCA2	0.166
2325	COMMD4	0.166
2326	WBP4	0.166
2327	GLOD4	0.166
2328	PUS3	0.165
2329	DENND5A	0.165
2330	MTA3	0.165
2331	C7orf73	0.165
2332	FASTKD5	0.165
2333	ERGIC2	0.165
2334	ZMYND19	0.165
2335	UBXN2B	0.165
2336	HSPA4L	0.165
2337	EIF3J	0.165
2338	SDAD1	0.165
2339	NDUFS1	0.165
2340	SUZ12P	0.165
2341	COMMD10	0.165
2342	TWF1	0.165
2343	KPNA1	0.165
2344	MAGI3	0.165

2345	GPATCH2	0.165
2346	SMEK2	0.164
2347	METTL21A	0.164
2348	HOMER1	0.164
2349	NFYA	0.164
2350	WRAP73	0.164
2351	RP11.180C16.1	0.164
2352	DCUN1D4	0.164
2353	TOP1	0.164
2354	JAGN1	0.164
2355	PGK1	0.164
2356	C2CD5	0.164
2357	VAPA	0.164
2358	BUD13	0.164
2359	DHX35	0.164
2360	ASH2L	0.164
2361	RC3H2	0.164
2362	FAHD2B	0.164
2363	FKTN	0.164
2364	BAZ1B	0.164
2365	UBIAD1	0.164
2366	ALKBH8	0.164
2367	KB.431C1.4	0.163
2368	C11orf84	0.163
2369	LYSMD1	0.163
2370	TNPO3	0.163
2371	TXNL1	0.163
2372	SREK1IP1	0.163
2373	NCAPH2	0.163
2374	UBXN8	0.163
2375	AHCYL1	0.163
2376	C4orf21	0.163
2377	XPR1	0.163
2378	CCDC47	0.163
2379	GTF2B	0.163
2380	RABIF	0.163
2381	TMEM167A	0.163
2382	TSEN15	0.163
2383	PGRMC1	0.163
2384	CCDC15	0.163
2385	ZNF584	0.163
2386	EARS2	0.163
2387	ATF1	0.163
2388	BBS4	0.163
2389	CTNNB1	0.163
2390	TOR1AIP1	0.162
2391	NDUFAF5	0.162
2392	RPS24	0.162

2393	STXBP3	0.162
2394	HEATR3	0.162
2395	SNX16	0.162
2396	CRKL	0.162
2397	NGDN	0.162
2398	ACADM	0.162
2399	NTAN1	0.162
2400	ANKRD46	0.162
2401	RNF14	0.162
2402	EPB41L5	0.162
2403	DHX30	0.162
2404	NSA2	0.162
2405	C11orf30	0.162
2406	ZNF37A	0.162
2407	CPSF6	0.162
2408	ARL1	0.161
2409	KDM1B	0.161
2410	RP3.508I15.9	0.161
2411	PET117	0.161
2412	TTLL4	0.161
2413	KIAA1715	0.161
2414	AC016773.1	0.161
2415	CBWD1	0.161
2416	SAP18	0.161
2417	RPL12P1	0.161
2418	LCLAT1	0.161
2419	APIP	0.161
2420	CDK5R1	0.161
2421	WAPAL	0.161
2422	SOAT1	0.161
2423	RP11.423H2.1	0.161
2424	SFXN4	0.161
2425	CFDP1	0.161
2426	TUBGCP4	0.161
2427	FIG4	0.161
2428	RBM17	0.160
2429	INIP	0.160
2430	RPL23AP79	0.160
2431	FAM208B	0.160
2432	GLE1	0.160
2433	RFXAP	0.160
2434	CDCA4	0.160
2435	PTBP1	0.160
2436	C17orf75	0.160
2437	FAM199X	0.160
2438	PFAS	0.160
2439	THAP9.AS1	0.160
2440	OSGIN2	0.160

2441	TMEM50B	0.160
2442	IBTK	0.160
2443	UTP14A	0.160
2444	BCORL1	0.160
2445	UBE2E1	0.160
2446	CBWD3	0.159
2447	MIB1	0.159
2448	ZNHIT6	0.159
2449	MARCKSL1	0.159
2450	ADK	0.159
2451	DROSHA	0.159
2452	AL357673.1	0.159
2453	ANKRD26	0.159
2454	RPS18P9	0.159
2455	EPT1	0.159
2456	CRLS1	0.159
2457	MCPH1	0.159
2458	COX15	0.159
2459	GPM6B	0.159
2460	ZNF436	0.159
2461	ZBTB24	0.159
2462	SNRNP25	0.159
2463	RAP1A	0.159
2464	XXbac.BPG181	0.159
2465	TRNAU1AP	0.159
2466	CAD	0.159
2467	SNX6	0.159
2468	RP11.316M21.6	0.158
2469	MRPL46	0.158
2470	COA4	0.158
2471	TMX1	0.158
2472	NT5DC1	0.158
2473	ANKRD39	0.158
2474	SEMA4F	0.158
2475	MEX3D	0.158
2476	PGM2	0.158
2477	PSMD2	0.158
2478	LIG3	0.158
2479	GPATCH11	0.158
2480	C12orf60	0.158
2481	MCTS1	0.158
2482	CYB5B	0.158
2483	CHUK	0.158
2484	HADHB	0.158
2485	TOMM34	0.158
2486	MKKS	0.158
2487	OTUD3	0.158
2488	LSM11	0.158

2489	ATXN10	0.158
2490	CDK16	0.158
2491	EIF4H	0.157
2492	SMPD4	0.157
2493	STX6	0.157
2494	KREMEN1	0.157
2495	RPL41	0.157
2496	ELMOD2	0.157
2497	NDUFB8	0.157
2498	C19orf12	0.157
2499	AC009005.2	0.157
2500	RBM3	0.157
2501	UAP1	0.157
2502	SWT1	0.157
2503	PFN2	0.157
2504	GAPDHP1	0.157
2505	TRAF3IP2.AS1	0.157
2506	KCNIP4	0.157
2507	THUMPD1	0.157
2508	RPS6KA5	0.157
2509	AAR2	0.157
2510	RP11.27I1.4	0.157
2511	SENP2	0.157
2512	AC010976.2	0.157
2513	GID4	0.157
2514	PRKAG1	0.157
2515	PPP6R3	0.157
2516	RPS15A	0.156
2517	PIK3CB	0.156
2518	BLOC1S2	0.156
2519	CEP170	0.156
2520	RP11.977G19.1	0.156
2521	TMEM170A	0.156
2522	ZHX1	0.156
2523	DCP1A	0.156
2524	POMGNT2	0.156
2525	IDE	0.156
2526	N6AMT1	0.156
2527	COPS6	0.156
2528	SLC25A40	0.156
2529	RAB18	0.156
2530	PPM1G	0.156
2531	SLC25A5	0.156
2532	FARP2	0.156
2533	KIAA0586	0.156
2534	SRP14	0.156
2535	WDFY1	0.156
2536	RINT1	0.156

2537	CEP44	0.156
2538	ATP7B	0.155
2539	DHTKD1	0.155
2540	RP1.152L7.5	0.155
2541	ALDH1B1	0.155
2542	ZNF250	0.155
2543	IQCC	0.155
2544	MTMR4	0.155
2545	DIXDC1	0.155
2546	KHSRP	0.155
2547	CTC.524C5.2	0.155
2548	CTC.366B18.2	0.155
2549	CCDC18	0.155
2550	FBXO30	0.155
2551	UBR2	0.155
2552	ZNF565	0.155
2553	PES1	0.155
2554	NABP2	0.154
2555	PPWD1	0.154
2556	GPAM	0.154
2557	CEP19	0.154
2558	ARIH2	0.154
2559	LRRC57	0.154
2560	BCAP29	0.154
2561	PSMC5	0.154
2562	VCP	0.154
2563	EPRS	0.154
2564	PHACTR4	0.154
2565	TPGS2	0.154
2566	PRDX1	0.154
2567	CLPX	0.154
2568	PDHX	0.154
2569	XRCC4	0.154
2570	CIRH1A	0.154
2571	EIF4G2	0.154
2572	TXNL4A	0.154
2573	RABL5	0.154
2574	PSPH	0.154
2575	PPIP5K2	0.154
2576	RPS27A	0.153
2577	INCENP	0.153
2578	PGAM1	0.153
2579	MTR	0.153
2580	ZNF45	0.153
2581	GTF2IP1	0.153
2582	KLHL7	0.153
2583	HNRNPU.AS1	0.153
2584	RHEB	0.153

2585	FAM222A	0.153
2586	EAF1.AS1	0.153
2587	CTB.96E2.3	0.153
2588	TATDN3	0.153
2589	IQCH	0.153
2590	RBBP8	0.153
2591	RHEBL1	0.153
2592	ESCO1	0.153
2593	ELOF1	0.153
2594	CMPK1	0.153
2595	TDP1	0.153
2596	NSMCE4A	0.153
2597	RRP7A	0.153
2598	ZNF207	0.153
2599	SMARCD1	0.153
2600	TRAPPC11	0.153
2601	RHOA	0.152
2602	ZNF714	0.152
2603	COX20	0.152
2604	KDM3B	0.152
2605	RTTN	0.152
2606	FAM206A	0.152
2607	MIER1	0.152
2608	SGTA	0.152
2609	DDB1	0.152
2610	RXRB	0.152
2611	SFMBT1	0.152
2612	RP11.517B11.7	0.152
2613	LEO1	0.152
2614	SMC5	0.152
2615	MRP63	0.152
2616	CRY1	0.152
2617	AKR7A2	0.152
2618	GPX4	0.152
2619	TDRD3	0.152
2620	CDON	0.152
2621	FTSJ2	0.152
2622	DNAAF2	0.152
2623	MTMR2	0.152
2624	YY1	0.152
2625	RAB7L1	0.152
2626	EXOSC5	0.152
2627	FECH	0.151
2628	AC034193.5	0.151
2629	PHYH	0.151
2630	COX5B	0.151
2631	CHTOP	0.151
2632	IARS	0.151

2633	KIF3A	0.151
2634	ZNF148	0.151
2635	AC006547.8	0.151
2636	COMMD5	0.151
2637	COX7C	0.151
2638	ICK	0.151
2639	SLC35B4	0.151
2640	HIAT1	0.151
2641	RANBP2	0.151
2642	ACTR1A	0.151
2643	IP6K1	0.151
2644	PTS	0.150
2645	TAMM41	0.150
2646	IPP	0.150
2647	TOP3A	0.150
2648	DCAF16	0.150
2649	RPL38	0.150
2650	PSMC3	0.150
2651	TP53BP2	0.150
2652	MIOS	0.150
2653	RAD51B	0.150
2654	TUBA1A	0.150
2655	TBC1D7	0.150
2656	EEF1A1P4	0.150
2657	LARP7	0.150
2658	HAX1	0.150
2659	GTPBP10	0.150
2660	RPS25	0.150
2661	RSL24D1	0.150
2662	RP11.864J10.4	0.150
2663	ANKHD1	0.150
2664	ZFX	0.150
2665	FKBP5	0.150
2666	RBM22	0.150
2667	RPL9P25	0.149
2668	HNRNPH1	0.149
2669	SAP30BP	0.149
2670	NOC2L	0.149
2671	GPN1	0.149
2672	DUSP11	0.149
2673	COQ2	0.149
2674	TMEM14C	0.149
2675	SLC30A5	0.149
2676	TMEM60	0.149
2677	HADHA	0.149
2678	AEBP2	0.149
2679	FUNDC1	0.149
2680	RP11.126K1.8	0.149

2681	MRPS14	0.149
2682	COPS5	0.149
2683	FOXN2	0.149
2684	CSRNP2	0.149
2685	RMND5A	0.149
2686	RP3.467N11.1	0.149
2687	DHRS13	0.149
2688	SACS	0.149
2689	SHPRH	0.149
2690	RP11.580I16.2	0.149
2691	PHF23	0.149
2692	COPZ1	0.149
2693	CTC.453G23.5	0.149
2694	TRMU	0.149
2695	PEF1	0.149
2696	FAM115A	0.149
2697	C19orf48	0.149
2698	RNF216P1	0.149
2699	SC5D	0.149
2700	RP11.575L7.8	0.149
2701	XXbac.BPGBP(	0.149
2702	MFAP3	0.149
2703	MED4.AS1	0.149
2704	HIP1	0.148
2705	RP11.24C3.2	0.148
2706	C6orf47	0.148
2707	RGS10	0.148
2708	CDKL3	0.148
2709	CCDC66	0.148
2710	RP5.1024G6.2	0.148
2711	SFXN2	0.148
2712	LIN54	0.148
2713	ZCCHC4	0.148
2714	E2F2	0.148
2715	FAM102B	0.148
2716	PTK2	0.148
2717	TIMM13	0.148
2718	PTP4A2	0.148
2719	DBF4P1	0.148
2720	SIM2	0.148
2721	TBCD	0.148
2722	EVI5	0.148
2723	STARD7	0.148
2724	CUL4B	0.147
2725	USP21	0.147
2726	PLGRKT	0.147
2727	RP11.649A18.1	0.147
2728	ASNA1	0.147

2729	PRMT10	0.147
2730	NDUFS4	0.147
2731	HADH	0.147
2732	RAB14	0.147
2733	PHF14	0.147
2734	LEPROTL1	0.147
2735	CHPT1	0.147
2736	ZSCAN21	0.147
2737	EIF3CL	0.147
2738	TUBB6	0.147
2739	CYB5R4	0.147
2740	RP11.229P13.2[illegible]	0.147
2741	KPTN	0.147
2742	APLP1	0.147
2743	PUM2	0.147
2744	AAMP	0.147
2745	RSBN1L	0.147
2746	IL17D	0.147
2747	RP11.362K14.5	0.147
2748	TCTN3	0.146
2749	RP5.837J1.2	0.146
2750	ALG8	0.146
2751	SCD	0.146
2752	AC108488.3	0.146
2753	BZW2	0.146
2754	ST6GALNAC6	0.146
2755	DHRS11	0.146
2756	MTMR9	0.146
2757	EWSR1	0.146
2758	CEP57	0.146
2759	POLR3K	0.146
2760	SHCBP1	0.146
2761	MRPS11	0.146
2762	FUNDC2	0.146
2763	PKN3	0.146
2764	EIF4EBP1	0.146
2765	DAG1	0.145
2766	THYN1	0.145
2767	C22orf39	0.145
2768	GMCL1	0.145
2769	ZNF689	0.145
2770	FUBP1	0.145
2771	RP11.697E2.7	0.145
2772	PIGC	0.145
2773	DSTYK	0.145
2774	WHSC1	0.145
2775	RNMT	0.145
2776	DVL2	0.145

2777	STAM	0.145
2778	GUF1	0.145
2779	ANAPC4	0.145
2780	FAM69B	0.145
2781	C1orf43	0.145
2782	PDCL	0.145
2783	EIF2B4	0.145
2784	RPL19	0.145
2785	FAM117B	0.145
2786	NRF1	0.145
2787	ZNF12	0.145
2788	FYTTD1	0.145
2789	CDK5RAP1	0.145
2790	UBAC1	0.144
2791	SMAD9	0.144
2792	COG2	0.144
2793	INPP5F	0.144
2794	ATP6V0E2	0.144
2795	WDR26	0.144
2796	CECR5	0.144
2797	CASC7	0.144
2798	MRPS33	0.144
2799	VPRBP	0.144
2800	POLR3B	0.144
2801	RBFOX2	0.144
2802	POLR1A	0.144
2803	FAM114A2	0.144
2804	MYH10	0.144
2805	RP4.622L5.2	0.144
2806	TRAK2	0.144
2807	TMEM81	0.144
2808	TYRO3	0.144
2809	POLR2C	0.144
2810	GNA13	0.143
2811	ZNF281	0.143
2812	Sep-10	0.143
2813	PPP2R4	0.143
2814	DYNLT1	0.143
2815	CSRP2BP	0.143
2816	LTN1	0.143
2817	TRIT1	0.143
2818	USP48	0.143
2819	THOC2	0.143
2820	CHAMP1	0.143
2821	APEX1	0.143
2822	PSME4	0.143
2823	FAM86A	0.143
2824	DNAJB5	0.143

2825	BPNT1	0.143
2826	RPL37A	0.143
2827	SCAI	0.143
2828	PTP4A1	0.143
2829	TTC28.AS1	0.143
2830	ZNF614	0.143
2831	ZNF555	0.143
2832	LIN7C	0.142
2833	UBE2A	0.142
2834	SQLE	0.142
2835	SNX17	0.142
2836	SNUPN	0.142
2837	KLHL18	0.142
2838	C14orf119	0.142
2839	LIAS	0.142
2840	PRKACA	0.142
2841	TBCCD1	0.142
2842	HINT1	0.142
2843	CRHR1	0.142
2844	MAPKAP1	0.142
2845	NBPF24	0.142
2846	NADK2	0.142
2847	TRAPPC2	0.142
2848	FNTA	0.142
2849	CEP72	0.142
2850	SAP30	0.142
2851	CTC.462L7.1	0.142
2852	CCNT1	0.142
2853	MRPL54	0.142
2854	SMIM15	0.142
2855	CDK8	0.141
2856	SNRPA	0.141
2857	OXSM	0.141
2858	BRI3BP	0.141
2859	ZNF24	0.141
2860	MRPS6	0.141
2861	GSK3A	0.141
2862	H3F3AP4	0.141
2863	USP11	0.141
2864	PHTF1	0.141
2865	PMF1	0.141
2866	ZC3H10	0.141
2867	SUFU	0.141
2868	TUBB2A	0.141
2869	YTHDF3	0.141
2870	USP9X	0.141
2871	DUT	0.141
2872	ZNF749	0.141

2873	ARL8B	0.141
2874	OIP5.AS1	0.141
2875	NME6	0.141
2876	ICA1L	0.141
2877	CTC.498J12.3	0.140
2878	QKI	0.140
2879	RP1.317E23.7	0.140
2880	CAPZA2	0.140
2881	LINC00493	0.140
2882	ZNF106	0.140
2883	CCNB1IP1	0.140
2884	THADA	0.140
2885	DYNC2LI1	0.140
2886	EIF3D	0.140
2887	ZMAT5	0.140
2888	CTD.2555O16.4	0.140
2889	TRAF3IP1	0.140
2890	DDX55	0.140
2891	MSANTD3	0.140
2892	CLCC1	0.140
2893	NUDT2	0.140
2894	IMPA1	0.140
2895	CDT1	0.140
2896	ELF2	0.140
2897	SLC6A8	0.140
2898	ENTPD1	0.140
2899	CAMSAP1	0.140
2900	PPP2R1A	0.140
2901	ASAP1	0.140
2902	RP11.359B12.2	0.139
2903	TMED5	0.139
2904	PARD6B	0.139
2905	SCAMP1	0.139
2906	CCDC112	0.139
2907	RUVBL2	0.139
2908	MTX2	0.139
2909	MAGEF1	0.139
2910	FN3K	0.139
2911	CCZ1	0.139
2912	SCCPDH	0.139
2913	NSUN2	0.139
2914	TMEM241	0.139
2915	RPS8	0.139
2916	CXADR	0.139
2917	COX6B1	0.139
2918	IWS1	0.139
2919	RBPJ	0.139
2920	CYC1	0.139

2921	PPP2R3A	0.139
2922	CCDC126	0.139
2923	RP13.131K19.2	0.139
2924	C12orf52	0.139
2925	CCDC109B	0.139
2926	CCDC6	0.138
2927	MPP5	0.138
2928	FBXO21	0.138
2929	RPL26	0.138
2930	RPRD1B	0.138
2931	FAM120B	0.138
2932	HDGFRP3	0.138
2933	NPEPPS	0.138
2934	BCL2L12	0.138
2935	RPAIN	0.138
2936	CC2D2A	0.138
2937	TFB1M	0.138
2938	Sep-05	0.138
2939	NFYC	0.138
2940	POLR3A	0.138
2941	PDCD10	0.138
2942	FAM222B	0.138
2943	PPT2	0.138
2944	RGPD5	0.138
2945	GNB2L1	0.138
2946	RSAD1	0.138
2947	RTCB	0.138
2948	UBQLN2	0.138
2949	CGGBP1	0.138
2950	ZNF445	0.138
2951	FASN	0.138
2952	RFT1	0.138
2953	NLGN2	0.138
2954	INTS10	0.138
2955	TEX2	0.138
2956	C10orf2	0.138
2957	TM2D2	0.138
2958	C2orf49	0.137
2959	CAMLG	0.137
2960	PIGU	0.137
2961	PPP1R11	0.137
2962	CNP	0.137
2963	PDCD6IP	0.137
2964	AHI1	0.137
2965	SLAIN2	0.137
2966	CTD.2517M22.	0.137
2967	FAM208A	0.137
2968	CTD.2323K18.1	0.137

2969	SCP2	0.137
2970	CIAO1	0.137
2971	BCL9	0.137
2972	C7orf60	0.137
2973	MED22	0.137
2974	TMED8	0.137
2975	PRPS2	0.137
2976	SENP3	0.137
2977	UQCRB	0.137
2978	ARV1	0.137
2979	EIF4EBP2	0.137
2980	MPI	0.137
2981	ACYP1	0.137
2982	SF3A1	0.137
2983	STX8	0.137
2984	FAM35DP	0.137
2985	HSDL1	0.136
2986	IVNS1ABP	0.136
2987	DNMT1	0.136
2988	PEX1	0.136
2989	SKI	0.136
2990	ACPL2	0.136
2991	ZCCHC9	0.136
2992	FAM168A	0.136
2993	TMEM181	0.136
2994	FGFR4	0.136
2995	RPS6	0.136
2996	TTC5	0.136
2997	SETD9	0.136
2998	RPS20	0.136
2999	MCM9	0.136
3000	PTMA	0.136
3001	DDX6	0.136
3002	MMAB	0.136
3003	MAPK8IP1	0.136
3004	TIMM22	0.136
3005	TMTC4	0.136
3006	RIMS3	0.136
3007	AC040173.1	0.136
3008	NFS1	0.136
3009	ZFP90	0.136
3010	CINP	0.135
3011	RECQL4	0.135
3012	NBPF3	0.135
3013	CMTM4	0.135
3014	GTF2H1	0.135
3015	SCAF8	0.135
3016	ANKRD13C	0.135

3017	RAB12	0.135
3018	PRDX4	0.135
3019	ATG12	0.135
3020	MBTPS2	0.135
3021	MIEN1	0.135
3022	TAF15	0.135
3023	TBRG4	0.135
3024	LCORL	0.135
3025	RPS3	0.135
3026	RP11.2E11.9	0.135
3027	DZIP3	0.135
3028	NUDT1	0.134
3029	KATNAL1	0.134
3030	RECQL	0.134
3031	POLD2	0.134
3032	MAGED2	0.134
